# Baihe Gujin decoction ameliorates sepsis-induced acute lung injury through Nrf2/GPX4-mediated antioxidant defense and PPARα-driven metabolic reprogramming: a multi-omics investigation

**DOI:** 10.3389/fimmu.2026.1767881

**Published:** 2026-06-29

**Authors:** Kaiyuan Zhang, Yuqing Huang, Yuan Wu, Zhitao Yang, Fangyu Luo, Yu Han, Lan Zheng, Lingling Lv

**Affiliations:** 1 Department of Traditional Chinese Medicine, Shanghai Jiao Tong University School of Medicine Affiliated Ruijin Hospital, Shanghai, China; 2 Department of Traditional Chinese Medicine, Loujiang New City Hospital of Taicang (Taicang Branch of Ruijin Hospital Affiliated with Shanghai Jiao Tong University School of Medicine), Suzhou, China; 3 Department of Emergency, Ruijin Hospital, Shanghai Jiao Tong University School of Medicine, Shanghai, China

**Keywords:** Baihe Gujin decoction, MTOR signaling, multi-omics, Nrf2/GPX4 axis, PPARα, sepsis-induced acute lung injury

## Abstract

**Background:**

Sepsis-induced acute lung injury (ALI) represents the earliest and most severe complication of sepsis, with extraordinarily high mortality rates. Baihe Gujin decoction (BHGJD), a classical traditional Chinese medicine formula, has demonstrated protective effects against various pulmonary diseases, yet its therapeutic potential in sepsis-induced ALI remains unexplored.

**Methods:**

The chemical constituents of BHGJD were characterized by mass spectrometry, and potential protective mechanisms were predicted using network pharmacology. *In vivo*, a cecal ligation and puncture (CLP) mouse model was established to evaluate BHGJD’s protective effects through histopathological assessment and serum inflammatory cytokine analysis. Lung tissue RNA sequencing and untargeted metabolomics were performed to explore underlying molecular mechanisms, followed by validation of key proteins (HO-1, GPX4, PPARα, CPT1A, mTOR, and p-mTOR) by Western blot, immunohistochemistry, and immunofluorescence. Lung-deposited bioactive components were subsequently identified by mass spectrometry. *In vitro*, (+)-catechin, a representative lung-exposed constituent detected in lung tissue, was used to treat lipopolysaccharide-induced MLE-12 cells. Cellular levels of adenosine triphosphate, reactive oxygen species, and ferrous iron were measured. Mitochondrial ultrastructure was assessed by transmission electron microscopy, and fatty acid oxidation capacity was evaluated using the Seahorse palmitate oxidation assay, followed by validation of key proteins by Western blot.

**Results:**

BHGJD significantly ameliorated CLP-induced ALI, as evidenced by improved histological architecture and reduced inflammatory cytokine levels. Integrated multi-omics analysis and experimental validation revealed three interconnected protective mechanisms: 1) activation of an Nrf2/GPX4-associated antioxidant response, initially suggested by glutathione pathway enrichment, reduced lipid peroxidation-related metabolites, and restoration of redox and mitochondrial homeostasis; 2) PPARα/CPT1A-associated metabolic remodeling toward fatty acid utilization, supported by fatty acid transport/metabolism enrichment, coordinated changes in multiple acylcarnitine species, increased PPARα/CPT1A expression, and improved palmitate-supported mitochondrial respiration *in vitro*; and 3) reduction of inflammatory lipid mediators. Altered mTOR signaling accompanied these changes and may represent a convergent regulatory pathway.

**Conclusion:**

BHGJD alleviates sepsis-induced ALI through coordinated enhancement of Nrf2/GPX4-associated antioxidant defense, PPARα/CPT1A-linked metabolic reprogramming toward fatty acid utilization, and suppression of inflammatory lipid mediators. These findings provide an integrated mechanistic framework for the protective actions of BHGJD.

## 1 Introduction

Sepsis is a life-threatening organ dysfunction resulting from dysregulated host responses to infection (1) characterized by systemic dissemination disease and capable of causing multi-organ impairment (2). Sepsis-induced acute lung injury (ALI) and its most severe form, acute respiratory distress syndrome (ARDS), are devastating clinical conditions marked by refractory hypoxemia, respiratory distress, and non-cardiogenic pulmonary edema (3) that carry a high mortality rate. Currently, there are no proven treatments that can effectively arrest the pathological cascade encompassing sepsis, ALI/ARDS, and ultimately respiratory failure.

Oxidative stress-driven by excessive reactive oxygen species (ROS) generation in neutrophils, macrophages, and damaged lung parenchyma-has been identified as a key contributor to alveolar damage, capillary leak, and cell death in ALI (4). The thiol antioxidant system-particularly its principle component, glutathione (GSH)-plays a pivotal role in protecting lung tissue from ROS-mediated injury: depletion of alveolar GSH is observed in patients with ARDS, implicating a failure of endogenous antioxidant defenses in the progression of lung injury (5). On the other hand, patients with ALI/ARDS experience a variety of metabolic disturbances, such as mitochondrial dysfunction, amino acid metabolism, and lipid metabolism abnormalities (6). Impaired fatty acid metabolism and mitochondrial β-oxidation have emerged as important metabolic hallmarks in sepsis and ALI. In sepsis, the suppression of peroxisome proliferator-activated receptor α (PPARα) and related fatty acid oxidation (FAO) pathways leads to the accumulation of lipotoxic species and worsened organ dysfunction (7). In the lungs, defective FAO is linked to alveolar epithelial dysfunction and poor resolution of lung injury (8). These data suggest that enhancing endogenous antioxidant defenses (such as GSH concentrations) while promoting efficient FAO might ameliorate ROS-mediated lung damage and metabolic failure in sepsis-induced ALI.

Traditional Chinese medicine (TCM) has gained attention as a potential adjunctive approach for sepsis-induced ALI (9). Many single compounds or formulations derived from TCM have demonstrated protective effects through antioxidative actions and metabolic remodeling. For example, it was discovered that Qingfei Litan decoction increased GSH levels and decreased lipopolysaccharide (LPS)-activated ROS in lung epithelial A549 cells (10). Elsewhere, Chen et al. proved that esculetin can regulate macrophage polarization through metabolic reprogramming (inhibiting M1 glycolysis while enhancing M2 fatty acid β-oxidation), thereby inhibiting the inflammatory response in sepsis-induced ALI (11).

Baihe Gujin decoction (BHGJD) is a classic Chinese formula developed to protect lung function and treat lung discomfort that is composed of 10 kinds of herbs, including *Lilii Bulbus* (Baihe), *Rehmanniae Radix Praeparata* (Shudihuang), *Rehmanniae Radix* (Shengdihuang), *Ophiopogonis Radix* (Maidong), *Scrophulariae Radix* (Xuanshen), *Angelicae Sinensis Radix* (Danggui), *Paeoniae Radix Alba* (Baishao), *Latycodonis Radix* (Jiegeng), *Fritillariae Cirrhosae Bulbus* (Chuanbeimu), and *Glycyrrhizae Radix et Rhizoma* (Gancao). It is widely given to patients with lung cancer and has shown its effect in reducing the mortality hazard ratio as an adjunctive therapy (12). Moreover, one study revealed that changes in oxidative stress indexes as well as inflammatory factors in a cisplatin-induced acute kidney injury model were recovered to a normal state with BHGJD treatment, which worked by activating the phosphatidylinositol 3-kinase/protein kinase B pathway and suppressing the nuclear factor (NF)-κB/mitogen-activated protein kinase pathway (13).

In the present study, we aimed to systematically elucidate the protective effects and underlying mechanisms of BHGJD against sepsis-induced ALI. By integrating multi-omics approaches with *in vivo* and *in vitro* validation, we investigated whether BHGJD alleviates lung injury through modulation of oxidative stress responses and lipid metabolic reprogramming.

## 2 Materials and methods

### 2.1 UPLC-Q-TOF-MS/MS analysis of BHGJD

The chemical constituents of BHGJD were analyzed using an ultra-performance liquid chromatography coupled with quadrupole time-of-flight tandem mass spectrometry (UPLC-Q-TOF-MS/MS) system. Briefly, 10 mg of each sample was mixed with 500 μL of 50% methanol, vortexed for 1 min, and ultrasonicated for 40 min to ensure complete dissolution. After centrifugation at 12,000 rpm for 5 min at 4 °C, the supernatant was filtered through a 0.1-μm membrane to obtain the test solution.

Chromatographic separation was performed on a Waters ACQUITY UPLC T3 column (2.1 × 100 mm, 1.8 μm; Waters Corporation, Milford, MA, USA) with an online filter. The column temperature was maintained at 40 °C, and the flow rate was 0.2 mL/min. Also, the detection wavelengths were 254, 280, 327, and 350 nm, and the injection volume was 5 μL. The mobile phases consisted of (A) 0.1% formic acid in water and (B) 0.1% formic acid in acetonitrile for the positive ion mode and (A) water containing 2 mM of ammonium acetate and (B) acetonitrile for the negative ion mode, respectively. The gradient conditions were as follows: 0 min, 95% A and 5% B; 3 min, 95% A and 5% B; 5 min, 90% A and 10% B; 28 min, 60% A and 40% B; 50 min, 5% A and 95% B; 54 min, 5% A and 95% B; 54.2 min, 95% A and 5% B; and 60 min, 95% A and 5% B.

Mass spectrometry was conducted in both positive and negative electrospray ionization modes. The MS1 and MS2 acquisition rates were 6 and 12 spectra/s, respectively. The collision energies were set to 10 and 40 eV. The scan ranges were m/z 100–1200 for MS1 and m/z 50–1300 for MS2. Finally, source parameters were as follows: gas temperature, 320 °C; gas flow, 8 L/min; sheath gas flow, 12 L/min; sheath gas temperature, 350 °C; and capillary voltage, 4000 V (positive) or 3500 V (negative).

Raw data files were converted to abf format using the Analysis Base File Converter and processed with the Xcalibur 4.1 software (version 4.1; Thermo Fisher Scientific, Waltham, MA, USA) for peak alignment and deconvolution. Compound identification was performed by matching MS/MS spectra with online databases, including the Human Metabolome Database (HMDB), PubChem, TCMBank, the Encyclopedia of Traditional Chinese Medicine (ETCM), and the High-throughput Experiment- and Reference-guided Database of Traditional Chinese Medicine (HERB). Data alignment and compound identification were further refined using the MS-DIAL software (version 4.9; available at https://systemsomicslab.github.io/compms/msdial/main.html) with the following parameters: MS1 tolerance, 0.01 Da; retention time tolerance, 0.2 min; MS1 tolerance for identification, 0.005 Da; MS2 tolerance, 0.025 Da; and identification score cutoff, 80%. The identified compounds were cross-referenced with scholarly literature and database information for confirmation.

### 2.2 Network pharmacology

#### 2.2.1 Identification of common target genes between BHGJD and sepsis-induced ALI

The chemical constituents identified by UPLC-Q-TOF-MS/MS were first screened in the Traditional Chinese Medicine Systems Pharmacology Database (TCMSP) (https://tcmsp-e.com/). Compounds with an oral bioavailability of ≥30% and a drug-likeness of ≥0.18 were selected as active ingredients, and their corresponding target genes were obtained from the database. For compounds not available in TCMSP, chemical information was retrieved from PubChem (https://pubchem.ncbi.nlm.nih.gov/), and molecular structures were generated using the SwissADME database (https://www.swissadme.ch/) based on Simplified Molecular Input Line Entry System (SMILES) codes. Compounds with high gastrointestinal absorption and a bioavailability score of ≥0.55 points were retained as potential active components, and their target genes were subsequently obtained from PubChem. Because these parameters reflect general oral absorption- and drug-likeness-related properties rather than lung-specific exposure, the screened compounds were used only for hypothesis generation and target prioritization, not as direct evidence of pulmonary bioavailability or pulmonary targeting (14). Disease-related target genes were collected by searching DrugBank (https://go.drugbank.com/), GeneCards (https://www.genecards.org/), Online Mendelian Inheritance in Man (OMIM) (https://omim.org/), and the Therapeutic Target Database (TTD) (https://ttd.idrblab.cn/) using the keywords “sepsis acute lung injury,” “septic acute lung injury,” “sepsis-induced acute lung injury,” “acute lung injury,” “ALI,” “acute respiratory distress syndrome” and “ARDS.” Genes retrieved from GeneCards were filtered using a relevance score threshold (>5) to reduce noise and improve disease specificity. All gene symbols were combined using a union strategy, and duplicates were removed. Then, they were standardized to official UniProt IDs using the UniProt database (https://www.uniprot.org/). Finally, the intersection of drug-related and disease-related target genes was identified as the common targets for subsequent network pharmacology analysis.

#### 2.2.2 Construction of an herb-compound-target network

The herb-compound-target network was constructed to illustrate relationships among the constituent herbs, their putative active compounds, and the corresponding molecular targets. Briefly, lists of herbs, their screened active compounds, and the associated target genes were integrated into a three-layer interaction table (herb-compound; compound-target). The interaction table was imported into Cytoscape (version 3.10.3; Institute of Systems Biology, Seattle, WA, USA) to generate the bipartite/tri-partite network, where nodes represent herbs, compounds, or targets and edges represent herb–compound or compound–target associations. Network topology was analyzed using the NetworkAnalyzer tool (ManageEngine, Pleasanton, CA, USA) to obtain topological parameters (degree, betweenness centrality, closeness, etc.).

#### 2.2.3 Gene ontology and Kyoto encyclopedia of genes and genomes enrichment

GO and KEGG pathway enrichment analyses were performed in R (version 4.4.2; R Foundation for Statistical Computing, Vienna, Austria) using the clusterProfiler R package with the org.Hs.eg.db annotation. Input gene IDs were converted to Entrez IDs prior to analysis. Significant terms/pathways were defined as those with adjusted *P* values of <0.05. Results were summarized and visualized using the clusterProfiler and enrichplot R packages (with dotplots and barplots, respectively). According to the enrichment outcomes, metabolism-related genes were identified.

#### 2.2.4 Protein-protein interaction network

Metabolism-related genes were uploaded to the STRING database (version 12.0) for PPI retrieval using the species *Homo sapiens*. Interactions with a combined score of ≥0.4 points (medium confidence) were retained. The resulting network was downloaded in Tab-separated Values format and imported into Cytoscape for visualization and further analysis. Nodes were ranked by degree values, with higher degree values represented by darker colors and larger node sizes.

### 2.3 Preparation of BHGJD

BHGJD granules (15 g; Tianjiang Pharmaceutical Co., Ltd., Tianjin, China) were dissolved in distilled water to prepare stock solutions of 0.2250 and 0.1125 g/mL for the high- and low-dose groups, respectively. The prepared solutions were stored at 4 °C and protected from light until use.

### 2.4 Animals and models

Male C57BL/6J mice aged 8–10 weeks old and weighing between 20 and 24 g (purchased from Shanghai SLAC Laboratory Animal Co, Ltd., Shanghai, China) were fed for 7 days before experiments. The mice were kept under a 12-/12-h light–dark cycle with free access to food and water, under a temperature of 20-26°C and relative humidity of 40-70%.

A total of 30 mice were randomly assigned equally (n = 6) to the Control group, Model group, BHGJ-L group, BHGJ-H group, or Dex group. Mice in the Control and Model groups received normal saline by gavage once daily for 7 days, while mice in the BHGJ-L and BHGJ-H groups were given BHGJD granule solutions at 1.125 and 2.25 g/kg/d, respectively, for 7 consecutive days.

After pretreatment, sepsis-induced ALI was induced by cecal ligation and puncture (CLP) under isoflurane anesthesia (induction 3-4% and maintenance 1.5-2% by inhalation). Briefly, the cecum was exposed through a midline abdominal incision and ligated below the ileocecal valve while leaving the distal one-third intact, followed by double puncture with an 18-gauge needle. A small amount of fecal material was gently extruded to ensure patency. After surgery, all mice received 1 mL sterile saline subcutaneously for fluid resuscitation.

To maintain effective plasma drug levels, mice in the gavage groups were administered an additional dose on the day of modeling and the day of sacrifice. And mice in the Dex group received an intraperitoneal injection of dexamethasone (1 mg/kg) 1 hour after CLP. All mice were euthanized 24 h after CLP for sample collection by CO_2_ inhalation using a gradual-fill method at a flow rate of 20-30% of the chamber volume per minute. The 24-hour post-CLP time point was selected to capture the early inflammatory injury phase, as reflected by prominent pulmonary edema, inflammatory infiltration, and elevated circulating cytokines at sacrifice (15).

### 2.5 Histological analysis

Lung tissues were collected and fixed in 4% paraformaldehyde for 24 h, dehydrated, embedded in paraffin, and sectioned at a thickness of 5 μm. Sections were stained with hematoxylin and eosin (H&E) according to standard procedures, then dehydrated, cleared, and sealed with neutral resin. Histopathological changes, including alveolar structure disruption, inflammatory cell infiltration, and interstitial edema, were observed under a light microscope (E100; Nikon, Tokyo Metropolis, Japan).

### 2.6 Immunohistochemistry

Immunohistochemistry was performed to detect HO-1 and CPT1A expression in lung tissues. Paraffin-embedded lung sections (4 μm) were deparaffinized, rehydrated, and subjected to antigen retrieval. After blocking, the sections were incubated with a primary antibody against HO-1 and CPT1A overnight at 4 °C, followed by incubation with horseradish peroxidase–conjugated secondary antibodies. Immunoreactivity was visualized with 3,3’-diaminobenzidine, counterstained with hematoxylin, and imaged under a light microscope (Nikon).

### 2.7 Immunofluorescence staining

Immunofluorescence staining was used to evaluate Nrf2 and GPX4 expression. Lung tissue sections were deparaffinized, rehydrated, subjected to antigen retrieval, and blocked with 5% bovine serum albumin. Sections were then incubated with primary antibodies against Nrf2 and GPX4 overnight at 4 °C, followed by with fluorescently labeled secondary antibodies. Nuclei were counterstained with 4’,6-diamidino-2-phenylindole. Fluorescence images were captured using a fluorescence microscope (Axio Scope.A1; Zeiss, Oberkochen, Germany), and signal distribution and intensity were analyzed.

### 2.8 Lung wet/dry weight ratio

The middle lobe of each right lung was excised, blotted gently with filter paper to remove surface blood, and weighed immediately to obtain the wet weight (g). The samples were then dried at 60 °C for 48 h and reweighed to obtain the dry weight (g). The lung wet-to-dry weight ratio was calculated as: W/D = (wet weight/dry weight) × 100%.

### 2.9 Enzyme-linked immunosorbent assay

The levels of interleukin (IL)-6, tumor necrosis factor (TNF)-α, and IL-1β in serum were measured using commercial enzyme-linked immunosorbent assay kits (cat. nos. ABN-KA7082, A1010A0203, and A1010A0201; BioTNT, Shanghai, China), according to the manufacturer’s instructions. Briefly, serum was added to pre-coated 96-well plates. After incubation with detection antibodies and substrate solution, absorbance was measured at 450 nm using a microplate reader (Synergy Neo2; BioTek Instruments, Inc., Agilent, Santa Clara, CA, USA), and cytokine concentrations were calculated based on standard curves.

### 2.10 RNA sequencing

Lung tissues from mice in the Control and BHGJ-H groups were collected for RNA sequencing (n=3). Total RNA was extracted using TRIzol reagent (Invitrogen, Carlsbad, CA, USA). RNA concentration and purity were determined with a NanoDrop 2000 spectrophotometer (Thermo Fisher Scientific, Waltham, MA, USA), and RNA integrity was evaluated using an Agilent 2100 Bioanalyzer (Agilent Technologies, Santa Clara, CA, USA). RNA-sequencing libraries were constructed using the VAHTS Universal V6 RNA-sequencing Library Prep Kit according to the manufacturer’s instructions. Sequencing and preliminary data processing were performed by OE Biotech Co., Ltd. (Shanghai, China).

The libraries were sequenced on the NovaSeq 6000 platform (Illumina, San Diego, CA, USA) to generate 150-bp paired-end reads. Raw reads were filtered using fastp to obtain high-quality clean reads, which were subsequently aligned to the reference genome using HISAT2. Gene-expression levels were quantified as fragments per kilobase of transcript per million mapped reads, and read counts were obtained using HTSeq-count. Principal component analysis was performed using R (version 3.2.0) to evaluate sample consistency.

Differential expression analysis was conducted using DESeq2, with *P* < 0.05 and |fold change| > 1.5 defined as the criteria for identifying differentially expressed genes (DEGs). Hierarchical clustering analysis of DEGs was carried out using R. GO and KEGG pathway enrichment analyses were performed based on the hypergeometric distribution, and enrichment results were visualized using bar plots, bubble plots, and chord diagrams generated in R.

### 2.11 Liquid chromatography-tandem mass spectrometry–based untargeted metabolomics analysis

Lung tissues from mice in the Control and BHGJ-H groups were subjected to LC-MS/MS–based untargeted metabolomics analysis (n=6). Frozen samples were thawed at 4 °C, accurately weighed (10 mg), and extracted with pre-cooled methanol/water (4:1, v/v) containing L-2-chlorophenylalanine as an internal standard. Samples were homogenized with steel beads, sonicated in an ice-water bath, and extracted at −40 °C overnight. After centrifugation, the supernatants were collected for LC-MS analysis. A pooled quality control sample was prepared by mixing equal aliquots of all samples.

Metabolomic profiling was performed using an ACQUITY UPLC I-Class system coupled to a Q-Exactive mass spectrometer (Thermo Fisher Scientific). Chromatographic separation was achieved on an HSS T3 column (2.1 × 100 mm, 1.8 µm) using a gradient of 0.1% formic acid in water (A) and acetonitrile (B). The gradient conditions were as follows: 0 min, 95% A and 5% B; 2 min, 95% A and 5% B; 4 min, 70% A and 30% B; 8 min, 50% A and 50% B; 10 min, 20% A and 80% B; 14 min, 100% B; 15 min, 100% B; 15.1 min, 95% A and 5% B; and 16 min, 95% A and 5% B. The flow rate was 0.35 mL/min, the column temperature was set at 45 °C, and the injection volume was 5 µL. Data were acquired in both positive and negative electrospray ionization modes over an m/z range of 100–1000.

Raw LC-MS data were processed using Progenesis QI (Waters Corporation) for peak detection, alignment, and normalization. Metabolite annotation was performed using HMDB, LipidMaps, METLIN, and in-house databases. Multivariate analyses, including principal component analysis and orthogonal partial least squares–discriminant analysis, were conducted using R software. Differential metabolites were identified based on a variable importance in projection of VIP>1, *P* < 0.05, and a fold change of >1.2 or <0.83, followed by KEGG pathway enrichment analysis.

### 2.12 Western blot analysis

After extracting proteins from lung tissues or MLE-12 cells, the protein concentrations were determined using a bicinchoninic acid protein assay kit (cat no. ZJ102; Epizyme, Cambridge, MA, USA). Equal amounts of protein samples were separated on 10% sodium dodecyl sulfate–polyacrylamide gel electrophoresis gels and subsequently transferred onto polyvinylidene fluoride (PVDF) membranes. The membranes were blocked with Tris-buffered Saline with Tween-20 containing 5% non-fat milk for 30 min. Subsequently, the PVDF membranes were incubated with antibodies against HO-1, GPX4, PPARα, CPT1A, mTOR, p-mTOR, and β-actin overnight at 4°C. After incubating, the PVDF membranes with the corresponding secondary antibodies at room temperature for 1 h, chemi-luminescent imaging and analysis were performed using a chemiluminescence imaging system (5200; Tanon Science & Technology Co., Ltd., Shanghai, China), and quantification analysis was carried out using the ImageJ software (US National Institutes of Health, Bethesda, MD, USA). The antibodies used are presented in **Supplementary Table 1**.

### 2.13 UPLC-Q-TOF-MS analysis of lung tissues

The lung tissues of Control group and BHGJ-H group mice were used for UPLC-Q-TOF-MS analysis. Chromatographic separation was performed on a Welch Ultimate UHPLC AQ-C18 column (2.1 × 100 mm, 1.8 µm; Waters Corporation, Milford, MA, USA) maintained at 30 °C. The mobile phase encompassed both acetonitrile (A) and water containing 0.1% formic acid (B), delivered at a flow rate of 0.3 mL/min with an injection volume of 5 µL. The gradient conditions were as follows: 0–3 min, 0-3% A and 100-97% B; 3–7 min, 3-8% A and 97-92% B; 7–15 min, 8-15% A and 92-85% B; 15–26 min, 15-25% A and 85-75% B; 26-32min, 25-45% A and 75-55% B; 32–40 min, 45-65% A and 55-35% B; 40-43min, 65-95% A and 35-5% B; 43-46min, 95% A and 5% B; 46-46.1min, 95-0% A and 5-100% B; and 46.1–49 min, 100% B. Detection wavelengths ranged from 190–400 nm. Mass spectrometric analysis was carried out using electrospray ionization negative/positive ion modes (SCIEX, Framingham, MA, USA). Data acquisition was controlled by Analyst TF 1.7.1 software (AB Sciex, Framingham, MA, USA), and data processing was performed using PeakView 1.2 (AB Sciex). Compound identification was achieved by matching with the Natural Products HR-MS/MS Spectral Library 1.0 database, in combination with previously characterized compounds of BHGJD.

### 2.14 Cell counting kit-8 assay

MLE-12 cells were acquired from the Institute of Stem Cell Research within the Chinese Academy of Sciences (Shanghai, China) and cultured in Dulbecco’s modified Eagle medium with 10% fetal bovine serum. Cell viability was evaluated using a Cell Counting Kit-8 (CCK-8) assay (cat no. C0005; TargetMol Chemicals Inc., Boston, MA, USA). Briefly, cells were seeded into 96-well plates and allowed to adhere overnight. Cells were then treated with (+)-catechin (cat no. MB6626-1; MeilunBio, Dalian, China) (0, 3.75, 7.5, 15, 30, 60, 120, 240, 480, 960 μg/mL) for 4 h. After treatment, 10 µL of CCK-8 reagent was added to each well containing 100 µL of culture medium, followed by incubation at 37°C for 1–2 h. The absorbance was measured at 450 nm using a microplate reader. Cell viability was expressed as a percentage of the control group.

### 2.15 Measurement of intracellular ATP

Intracellular ATP levels were quantified using a luciferase-based ATP assay kit (cat no. SB-AM1001; ShareBio, Shanghai, China). Cells were cultured in 96-well plates and treated by (+)-catechin (0, 7.5, 15, 30, 60 μg/mL) with or without 5 μg/mL of LPS (cat no. IAX-100-006; Adipogen Life Sciences, San Diego, CA, USA) (16)for 4 h. Subsequently, 100 µL of reagent was added to each well, followed by shaking for 2 min. After incubation at room temperature in the dark for 15 min, the luminescence signal was recorded using a luminometer, and ATP levels were calculated based on a standard curve.

### 2.16 Measurement of intracellular ROS

Intracellular ROS levels were detected using 2’,7’-dichlorodihydrofluorescein diacetate (DCFH-DA) staining (cat no. S0033S; Beyotime Biotechnology, Shanghai, China). Briefly, approximately 2×10^5^ cells were treated by 5 μg/mL of LPS with (+)-catechin (0, 15, 30 μg/mL) for 4 h, then washed with phosphate-buffered saline (PBS) and incubated with 10 μM of DCFH-DA at 37 °C for 30 min in the dark. After incubation, cells were washed with PBS to remove excess probe. Fluorescence images were captured using an inverted fluorescence microscope (Axio Vert.A1; Zeiss, Oberkochen, Germany) with excitation/emission wavelengths of 488/525nm.

### 2.17 Cell Fe^2+^ Fluorometric Assay

Approximately 3×10^3^ cells were treated with 5 μg/mL of LPS with (+)-catechin (0, 15, 30 μg/mL) for 4 h. Then, cells were washed with PBS and incubated with 1 μM of Fe²^+^ detection reagent (cat no. MA0647; MeilunBio), ensuring full coverage of the monolayer. After incubation, the intracellular Fe²^+^-bound complex was detected by its characteristic orange-red fluorescence (excitation/emission wavelengths: 543/580 nm) using an inverted fluorescence microscope (Zeiss).

### 2.18 Transmission electron microscopy

Approximately 1 × 10^6 cells were treated with 5 μg/mL of LPS with (+)-catechin (0, 15 μg/mL) for 4 h and subsequently fixed with an electron microscopy–grade fixative at room temperature. After removal of the initial fixative, samples were re-fixed with fresh fixative in the dark for an additional 30 min. The cells were then post-fixed with osmium tetroxide in phosphate-buffered saline (PBS) for 2 h and rinsed three times with buffer. Following graded ethanol dehydration, samples were infiltrated with embedding resin overnight and polymerized to obtain embedded blocks. Ultrathin sections were prepared and subjected to double staining with uranyl acetate and lead citrate. Mitochondrial ultrastructure was examined using transmission electron microscopy.

### 2.19 Seahorse palmitate oxidation assay

Mitochondrial FAO capacity were assessed using the Seahorse XF Palmitate Oxidation Stress Test Kit (cat no. 103693-100; Agilent Technologies, Santa Clara, CA, USA). Briefly, MLE-12 cells were seeded into Seahorse XFe96 microplates at 8–10 × 10^4^ cells/well and treated with LPS (5 μg/mL) with (+)-catechin (0, 15 μg/mL) for 4 h prior to analysis. The untreated cells served as the control group. To assess FAO capacity, medium was replaced with FAO assay medium (Seahorse XF Base Medium supplemented with 0.5 mM L-carnitine, pH 7.4) for 60 min at 37 °C in a non-CO_2_ incubator. Palmitate-BSA conjugate (final palmitate concentration 150 μM) was then added as substrate control. Oxygen consumption rate (OCR) was measured using a Seahorse XFe96 Analyzer. Oligomycin (1.0 μM), FCCP (1.0 μM), and rotenone/antimycin A (0.5 μM each) were sequentially injected to determine mitochondrial respiratory parameters.

### 2.20 Statistics

All data were expressed using mean ± standard deviation values and analyzed using GraphPad Prism 10.0 (GraphPad Software, La Jolla, CA, USA) for statistical analysis and graph plotting. One-way analysis of variance was used for intergroup comparisons, and Dunnett’s test was performed for multiple comparisons between groups, with *P* < 0.05 considered statistically significant. All experiments were repeated three times.

## 3 Result

### 3.1 UPLC-Q-TOF-MS/MS identified 321 chemical constituents in BHGJD

**Figure 1:**
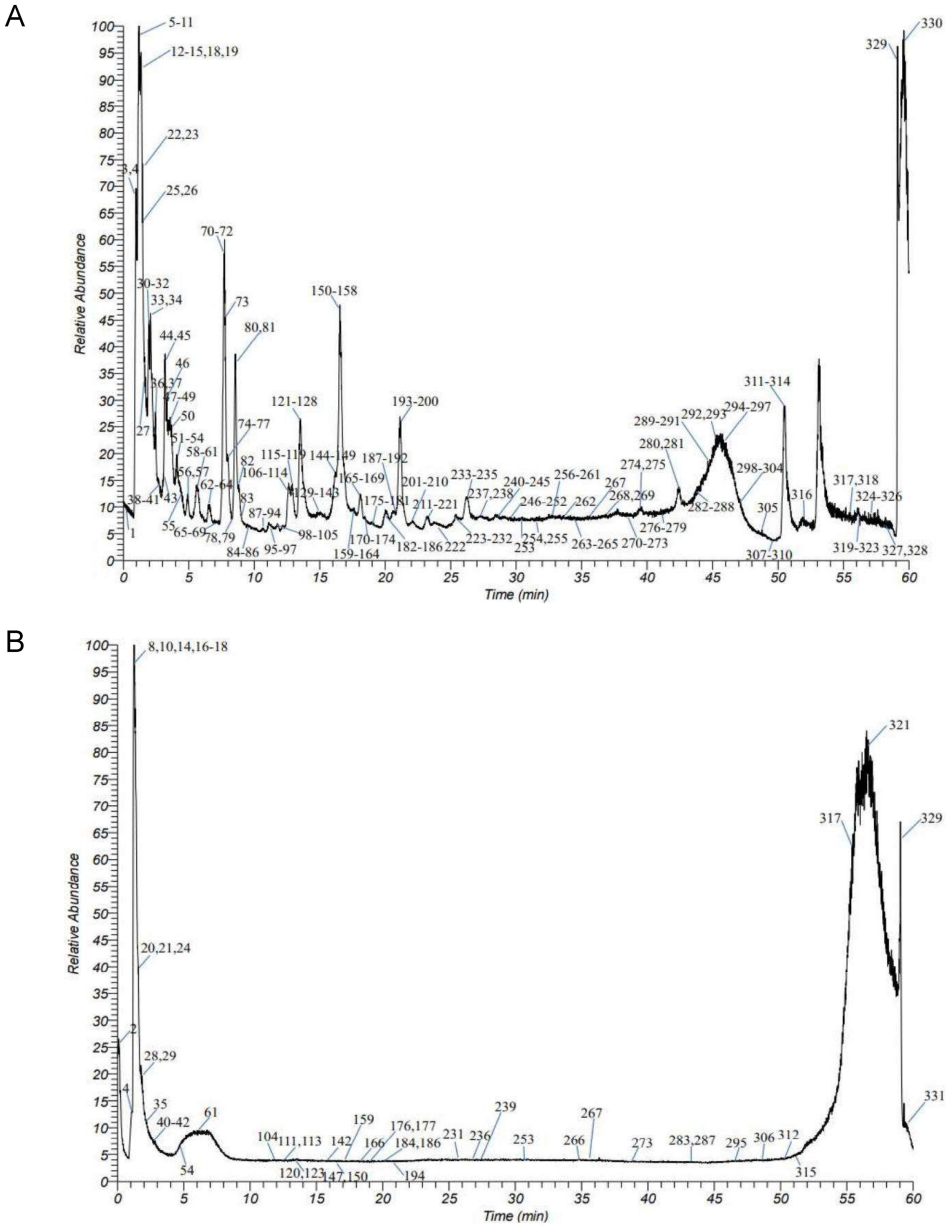
Total ion chromatogram of BHGJD. **(A)** Positive ion mode. **(B)** Negative ion mode.

Based on UPLC-Q-TOF-MS/MS profiling, 334 compounds were detected in Sample 1 and 331 were detected in Sample 2 (**Figure 1**). A total of 321 shared constituents were identified, including 42 flavonoids, 41 terpenoids, 29 phenols, 29 alkaloids, 28 organic acids, 24 coumarins, 20 amino acids, 17 fatty acids, 11 saponins, 9 phthalides, 8 nucleotides, 6 phenylpropanoids, 6 saccharides, 5 vitamins, 4 glycosides, 3 steroids, 2 lignans, and 37 others (**Supplementary Table 2**).

### 3.2 Network pharmacology predicts the potential mechanisms of BHGJD against sepsis-induced ALI

**Figure 2:**
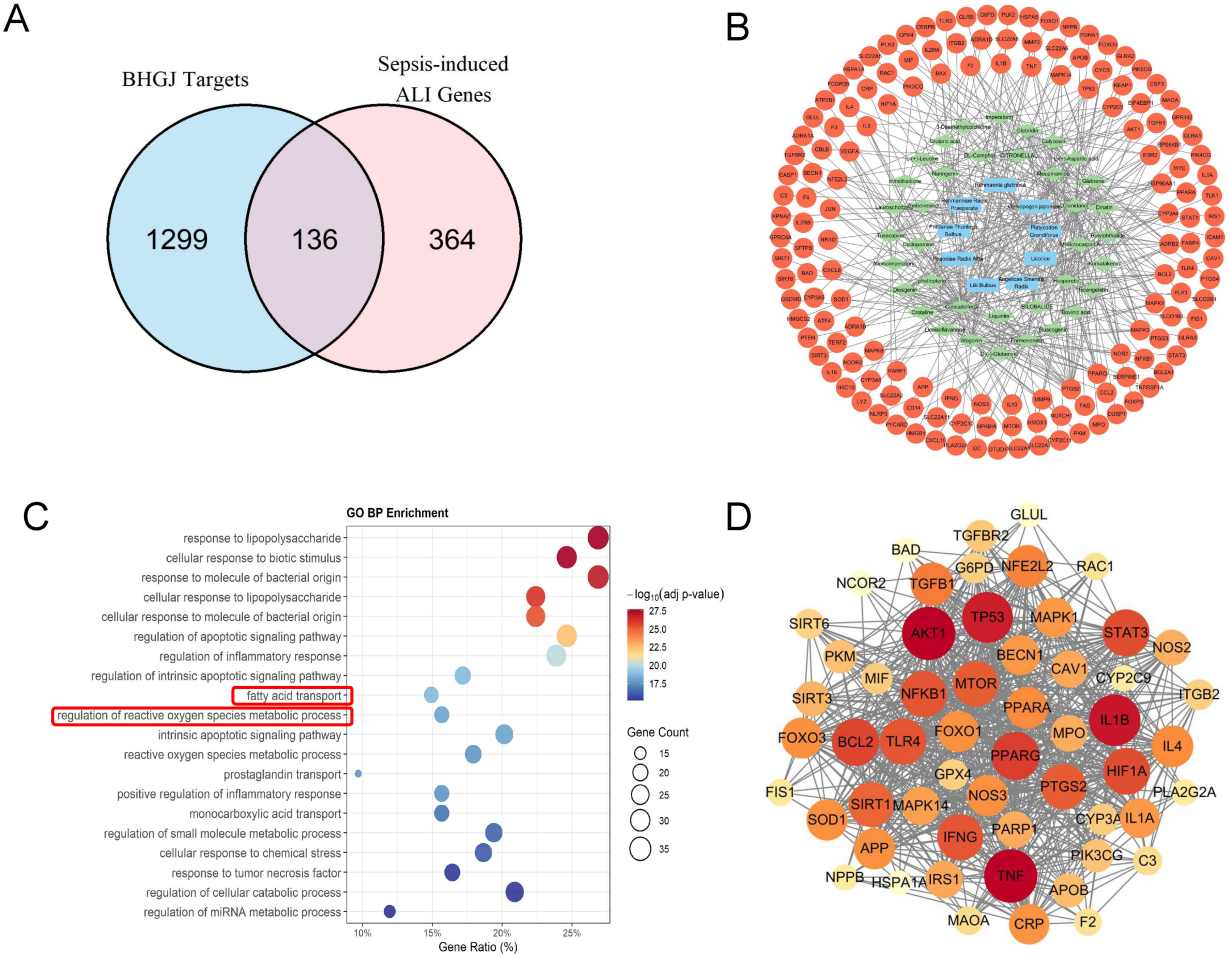
Network pharmacology analysis and prediction of the potential mechanisms and genes. **(A)** 136 overlapping genes were identified between BHGJD's targets and sepsis-induced ALI related genes. **(B)** The herb-compound- target interaction network. **(C)** The top 20 biological processes enriched by GO BP. Several metabolism-related processes were enriched. **(D)** The PPI network of 57 metabolism-related genes.

The aforementioned 321 compounds were subjected to TCMSP and PubChem searches, yielding 1,435 target genes. Using the keywords “sepsis acute lung injury,” “septic acute lung injury,” “sepsis-induced acute lung injury,” “acute lung injury,” “ALI,” “acute respiratory distress syndrome,” and “ARDS,” 500 disease-related genes were collected from DrugBank, GeneCards, OMIM, and TTD. Venn analysis identified 136 overlapping genes (**Figure 2A**).

An herb-compound-target interaction network was then constructed using Cytoscape (**Figure 2B**), where blue nodes represent the constituent herbs of BHGJD (excluding *Scrophulariae Radix* due to absence of available compounds/targets), green nodes represent active compounds, and orange nodes represent target genes.

Then, GO biological process and KEGG enrichment analyses were performed using R. **Figure 2C** presents the top 20 enriched biological processes, which include “response to lipopolysaccharides,” “cellular response to biotic stimulus,” and “response to a molecule of bacterial origin.” Besides inflammation, apoptosis, and host defense responses, several metabolism-related processes were also enriched, among which “fatty acid transport” and “regulation of ROS metabolic process” attracted our attention. To further investigate the metabolic features, 57 metabolism-related genes were extracted from the GO/KEGG results and subjected to STRING analysis to construct a PPI network, followed by visualization in Cytoscape (**Figure 2D**). In the network, darker and larger nodes indicate higher degree values, suggesting closer connectivity with other genes.

### 3.3 BHGJD exhibits protective effects in sepsis-induced ALI mice

**Figure 3:**
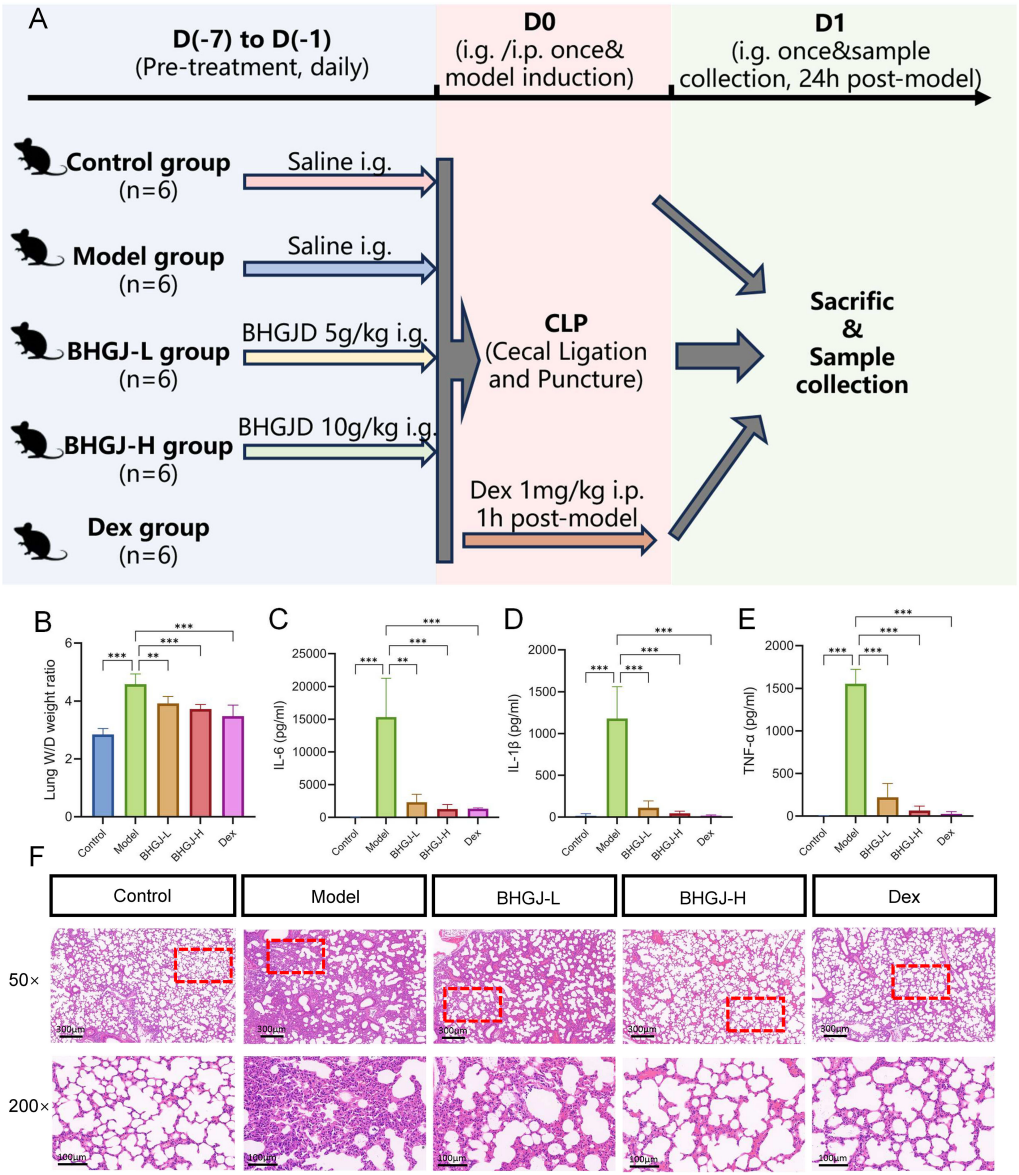
Protective effect of BHGJD in sepsis-induced ALI mice (n=6). **(A)** Diagram of animal experiments. **(B)** Lung wet/dry ratio. **(C–E)** Serum levels of inflammatory cytokines including lL-1, 1L-6, and TNF*-a.* **(F)** H&E staining of the lung tissue (50x, 200x). **p < 0.01, ***p < 0.001.

To determine whether BHGJD exerts protective effects *in vivo*, a CLP mouse model was established (**Figure 3A**). The lung W/D ratio was significantly increased in the model group compared to the control group, whereas BHGJD treatment reduced this ratio (**Figure 3B**). H&E staining revealed pronounced pathological changes in the model group, including pulmonary edema and widespread inflammatory infiltration. These lesions were alleviated in BHGJD-treated mice, with reduced edema and inflammatory cell infiltration (**Figure 3F**).

Serum levels of inflammatory cytokines, including IL-1β, IL-6, and TNF-α, were further assessed. Levels of these cytokines were markedly increased in the model group, whereas BHGJD reversed this trend (**Figures 3C–E**).

Collectively, these findings indicate that BHGJD ameliorates sepsis-induced ALI.

### 3.4 RNA-sequencing analysis

**Figure 4:**
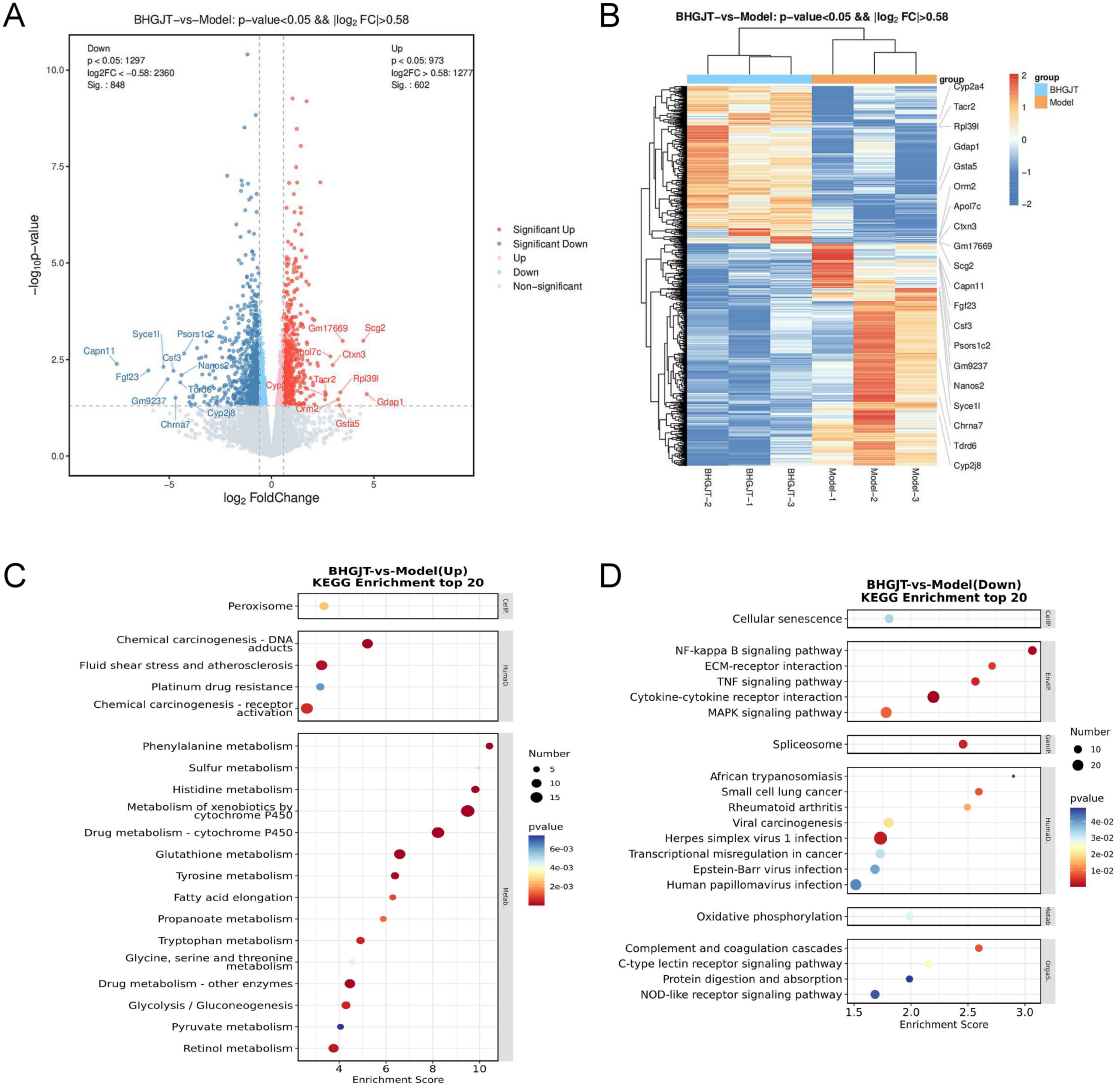
RNA-seq analysis of lung tissues from the model group and BHGJ-H group (n = 3). **(A)** Volcano plot showing 1,450 differentially expressed genes (DEGs) between the two groups. **(B)** Heatmap illustrating distinct clustering patterns of DEGs. **(C)** KEGG pathway enrichment analysis of upregulated genes, highlighting significant enrichment in metabolism­ related pathways, including glutathione metabolism and tatty acid metabolism. **(D)** KEGG pathway enrichment analysis of downregulated genes, showing enrichment in inflammation- and stress-related pathways.

RNA sequencing was performed on samples from the model and BHGJ-H groups. Using *P* < 0.05 and |log2FC| > 0.58 as cutoffs, 1,450 DEGs were identified in the BHGJ-H group relative to the model group, including 602 upregulated and 848 downregulated genes (**Figure 4A**). Hierarchical clustering showed clear separation between groups (**Figure 4B**).

To explore potential pathways involved, KEGG enrichment analysis were conducted separately for upregulated and downregulated genes. Interestingly, the upregulated genes were enriched in multiple metabolism-related pathways, including GSH metabolism, fatty acid metabolism, amino acid metabolism, and drug metabolism, indicating enhanced endogenous detoxification and metabolic reprogramming (**Figure 4C**). Conversely, the downregulated genes were mainly enriched in inflammation- and stress-related pathways, such as the NF-κB, TNF, mitogen-activated protein kinase, and oxidative phosphorylation pathways (**Figure 4D**).

Together, these results suggest that BHGJD alleviates sepsis-induced ALI by suppressing pro-inflammatory signaling and promoting metabolic remodeling, particularly GSH and lipid metabolism, which is consistent with the network pharmacology predictions.

### 3.5 Untargeted metabolomic analysis

**Figure 5:**
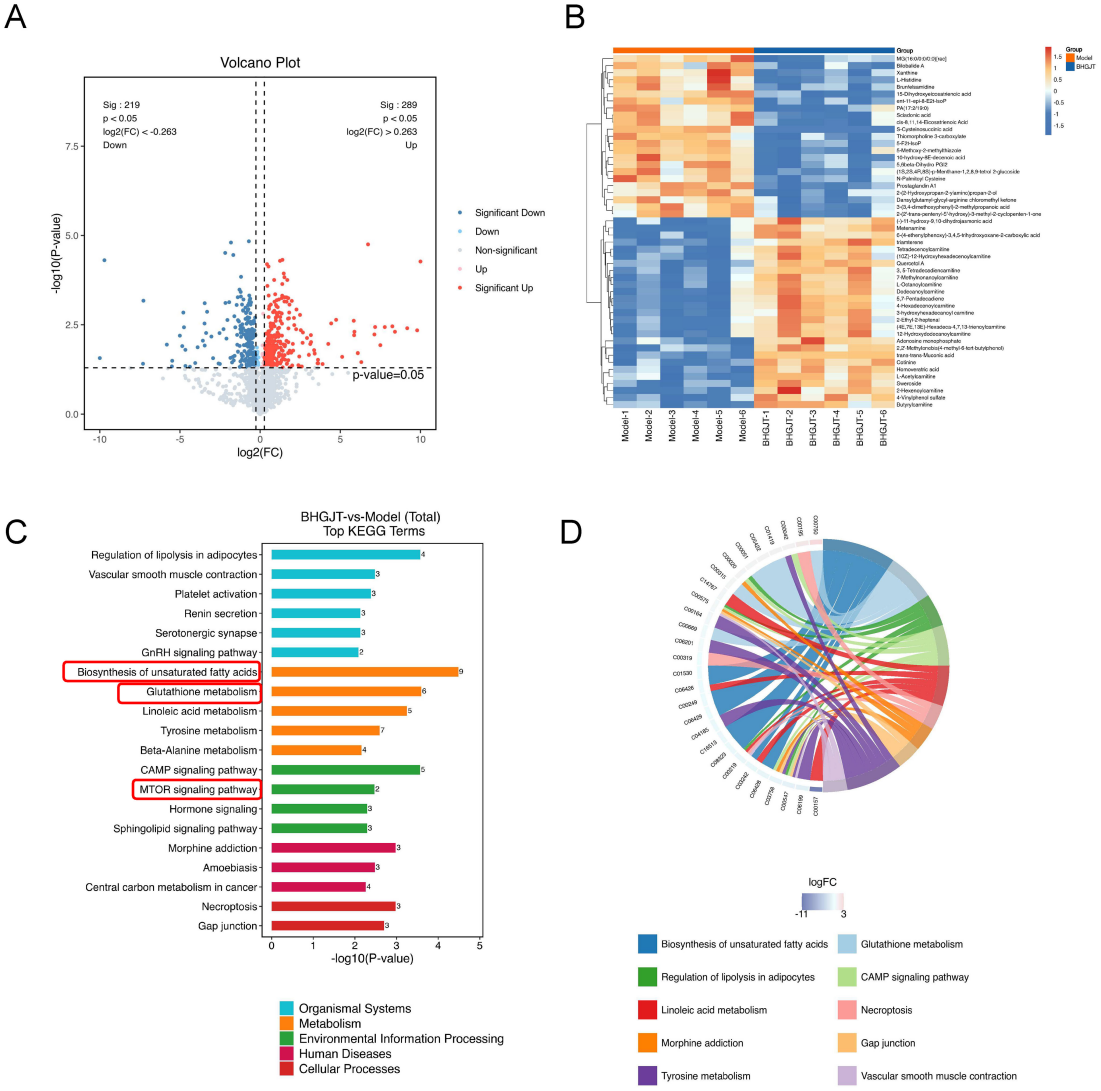
Untargeted metabolomic analysis of lung tissues from the model group and BHGJ-H group (n = 6). **(A)** Volcano plot showing 508 differential metabolites between the two groups. **(B)** Heatmap illustrating the top 50 differential metabolites and distinct metabolic profiles between groups. **(C, D)** KEGG pathway enrichment analysis of differential metabolites, highlighting significant enrichment in glutathione metabolism, biosynthesis of unsaturated fatty acids, and mTOR signaling pathway. BHGJD treatment was associated with reduced levels of lipid peroxidation-related metabolites and pro-inflammatory eicosanoids, accompanied by increased camitine species.

As both network pharmacology and RNA sequencing indicated metabolic involvement, untargeted metabolomic analysis was performed on the model and BHGJ-H groups. Using thresholds of *P* < 0.05 and |log2FC| > 0.263, a total of 508 differential metabolites were detected, including 289 upregulated and 219 downregulated metabolites (**Figure 5A**, **Supplementary Table 3**). Hierarchical clustering confirmed distinct metabolic patterns between groups (**Figure 5B**).

Several lipid peroxidation markers including 5-F2t-IsoP, ent-11-epi-8-E2t-IsoP, and multiple pro-inflammatory eicosanoids such as prostaglandin A1 were significantly decreased, while the concentration of the antioxidant metabolite sweroside was increased in the BHGJ-H group. On the other hand, several carnitine species, including butyrylcarnitine, dodecanoylcarnitine, and L-octanoylcarnitine, were elevated in the BHGJ-H group (**Figure 5B**). KEGG enrichment further supported enrichment of GSH metabolism, biosynthesis of unsaturated fatty acids, regulation of lipolysis in adipocytes, and linoleic acid metabolism (**Figures 5C, D**).

These results indicate that BHGJD may strengthen antioxidant defenses, reduce inflammatory lipid mediators, and promote fatty acid β-oxidation and metabolic remodeling, thereby mitigating oxidative and inflammatory injury in sepsis-induced ALI.

### 3.6 BHGJD regulates the Nrf2/GPX4 and PPARα/CPT1A axes

**Figure 6:**
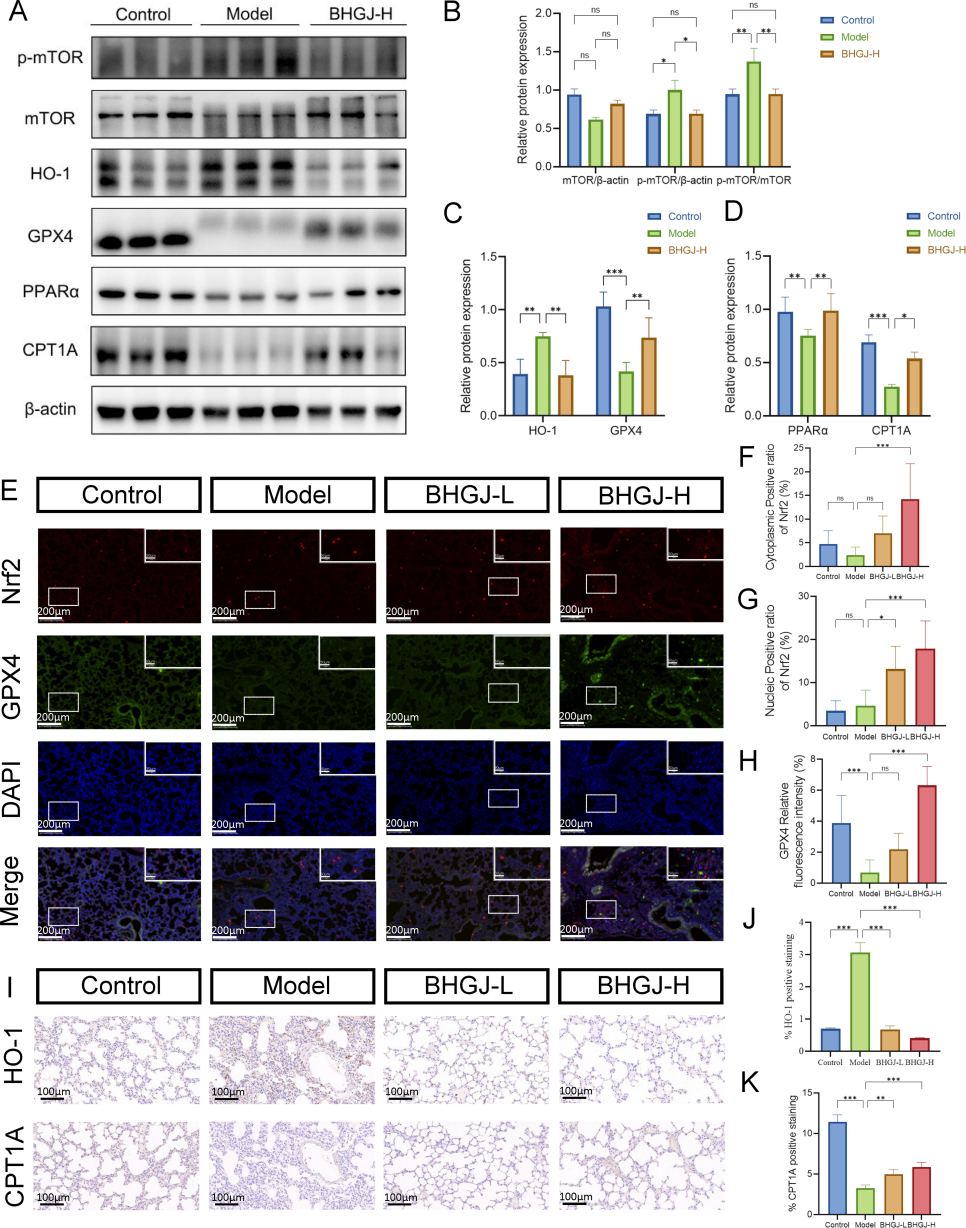
BHGJD regulates the Nrf2/GPX4 and PPARα/CPT1A axes in lung tissues (n = 6). **(A–D)** Representative western blot images and quantitative analysis of p-mTOR, mTOR, HO-1, GPX4, PPARα, and CPT1A expression levels. **(E–H)** Representative immunofluorescence (IF) images and quantitative analysis of Nrf2 and GPX4 expression. **(I–K)** Representative immunohistochemical (IHC) staining images and quantitative analysis of HO-1 and CPT1A expression. Data are presented as mean± SD. *p < 0.05, **p < 0.01, ***p < 0.001.

To validate the regulatory effects of BHGJD on oxidative stress and lipid metabolism pathways, the protein expression levels of HO-1, GPX4, PPARα, CPT1A, mTOR, and p-mTOR in lung tissues were examined. Compared to controls, the model group showed increased HO-1, decreased GPX4, PPARα, and CPT1A. BHGJD treatment did not elevate HO-1 levels but restored GPX4, PPARα, and CPT1A levels (**Figures 6A, C, D**). In addition, the elevated p-mTOR/mTOR ratio in the model group was effectively suppressed by BHGJD (**Figures 6A, B**), suggesting that BHGJD modulates mTOR-related signaling pathways to enhance antioxidant defenses.

Immunofluorescence staining revealed cytoplasmic Nrf2 in the control group, partial nuclear translocation in the model group, and pronounced nuclear accumulation after BHGJD treatment. GPX4 fluorescence was markedly reduced in the model group but significantly increased after BHGJD administration (**Figures 6E–H**).

Immunohistochemistry staining further validated key targets. CPT1A expression was markedly reduced in the model group, consistent with impaired fatty acid metabolism in sepsis-induced ALI. BHGJD treatment restored CPT1A levels, indicating recovery of CPT1A-mediated FAO, while HO-1 expression paralleled western blot results (**Figures 6I–K**).

Taken together, these data support the association of BHGJD protection with an Nrf2/GPX4-related antioxidant response and a PPARα/CPT1A-related metabolic remodeling program, while altered mTOR signaling may represent a convergent pathway accompanying these changes.

### 3.7 Pulmonary exposure profiling identified (+)-catechin as a priority candidate for initial *in vitro* validation

**Figure 7:**
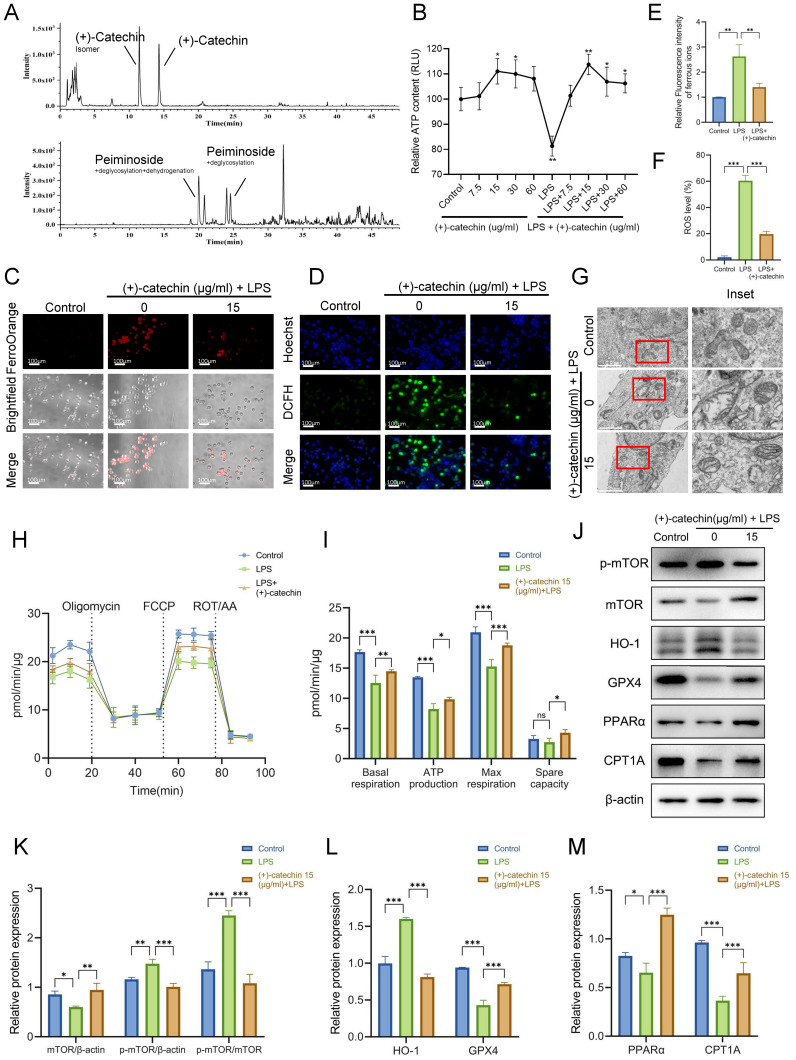
(+)-Catechin restores mitochondrial function and regulates antioxidant and FAO pathways in LPS-stimulated MLE-12 cells (n = 3). **(A)** (+)-Catechin is identified as the major lung-deposited component of BHGJD. **(B)** Cellular ATP levels were determined using a luminescence assay. **(C, E)** Ferrous iron levels were measured using a Fe 2+ detection kit. **(D, F)** Intracellular ROS levels were assessed by DCFH-DA staining. **(G)** Representative transmission electron microscopy (TEM) images showing mitochondrial ultrastructure. **(H, I)** Oxygen consumption rate (OCR) analysis and quantification of basal respiration, ATP production, max respiration, and spare capacity. **(J–M)** Representative western blot images and quantitative analysis of p-mTOR, mTOR, HO-1, GPX4, PPARα, and CPT1A expression levels. LPS stimulation induced mitochondrial structural damage, reduced ATP production, increased ROS and Fe2+ accumulation, and impaired FAO­ associated respiration, accompanied by altered expression of antioxidant- and FAO-related proteins, whereas (+)-catechin treatment markedly ameliorated these alterations. Data are presented as mean± SD. *p < 0.05, **p < 0.01, ***p < 0.001.

Pulmonary exposure profiling of lung tissues from mice in the BHGJ-H group identified five BHGJD-derived constituents (**Figure 7A**), comprising one parent compound, (+)-catechin, and four metabolites: a (+)-catechin isomer, one peiminoside-derived metabolite generated by deglycosylation and dehydrogenation, and two isomeric peiminoside-derived metabolites generated by deglycosylation. Among the constituents detected in lung tissue, (+)-catechin was the only parent compound and exhibited a clear, well-resolved chromatographic peak. It was therefore prioritized as a representative lung-exposed candidate for initial *in vitro* validation. This prioritization was intended to provide exposure-guided mechanistic support for a bioavailable pulmonary constituent, rather than to suggest that (+)-catechin alone accounts for the overall pharmacological effects of BHGJD.

### 3.8 (+)-catechin improves mitochondrial function and attenuates oxidative stress in LPS-stimulated MLE-12 cells

To perform an exposure-guided *in vitro* validation, LPS-stimulated alveolar epithelial MLE-12 cells were treated with (+)-catechin. CCK-8 assays showed that (+)-catechin at 3.75-240 μg/mL did not significantly affect cell viability (**Supplementary Figure 1**). The subsequent ATP assays showed that (+)-catechin increased ATP levels under basal conditions and significantly restored LPS-induced ATP depletion, even exceeding baseline levels (**Figure 7B**), which was most significant at a concentration of 15 μg/mL. Fluorescence assays further demonstrated that LPS markedly elevated intracellular ROS and Fe²^+^ levels, both of which were attenuated by (+)-catechin treatment (**Figures 7C–F**). And the following transmission electron microscopy showed that (+)-catechin alleviated mitochondria damage, evidenced by mitigatory vacuolization and increased mitochondrial cristae number (**Figure 7G**). These findings indicate that (+)-catechin can reproduce part of the protective phenotype of BHGJD *in vitro*, which enhances cellular energy metabolism and alleviates LPS-induced oxidative stress and ferroptosis-associated alterations in MLE-12 cells.

### 3.9 (+)-catechin enhances palmitate-driven mitochondrial respiration in LPS-stimulated MLE-12 cells

To evaluate mitochondrial FAO capacity, OCR was measured using palmitate as the primary exogenous substrate. Under palmitate-supported conditions, LPS stimulation significantly reduced basal respiratory, ATP production, and maximal respiratory capacity compared with control cells, indicating impaired mitochondrial oxidative metabolism. (+)-Catechin treatment significantly increased the three parameters as well as spare capacity in LPS-stimulated cells compared with LPS treatment alone (**Figures 7H, I**). These findings indicate that (+)-catechin restores mitochondrial respiratory function under fatty acid-supported conditions, consistent with an improvement in FAO-associated oxidative capacity.

### 3.10 (+)-catechin regulates Nrf2/GPX4 and PPARα/CPT1A Axes in LPS-stimulated MLE-12 cells

To further determine whether (+)-catechin recapitulates the molecular mechanisms observed *in vivo*, the expression of key proteins involved in the Nrf2/GPX4 and PPARα/CPT1A axes and mTOR pathway were examined in LPS-stimulated MLE-12 cells. Compared to controls, cells treated with LPS showed increased HO-1, decreased GPX4, PPARα, and CPT1A. (+)-Catechin treatment did not elevate HO-1 levels but restored GPX4, PPARα, and CPT1A levels (**Figures 7J, L, M**). In addition, the elevated p-mTOR/mTOR ratio in the LPS group was effectively suppressed by (+)-catechin (**Figures 7J, K**). Given the absence of pharmacokinetic bridging and multi-component interaction testing, these *in vitro* data were interpreted as support for partial mechanistic recapitulation rather than proof that (+)-catechin represents the full pharmacological activity of BHGJD.

## 4 Discussion

Sepsis triggers multi-organ injury, with ALI emerging as the earliest and most severe complication, subsequently progressing to ARDS and respiratory failure, resulting in extraordinarily high mortality rates. BHGJD, a classical TCM formula, possesses the therapeutic properties of nourishing the kidneys and moistening the lungs, as well as resolving phlegm and relieving cough. Contemporary research demonstrates its protective effects against various pulmonary diseases. Beyond its most common application in lung cancer treatment (17), BHGJD ameliorates idiopathic pulmonary fibrosis through proline metabolism modulation (18), serves as adjuvant therapy for pulmonary tuberculosis (19), and mitigates cisplatin-induced acute kidney injury (13). These findings led us to hypothesize that BHGJD might exert protective effects against sepsis-induced ALI. By integrating multi-omics results with functional assays, we reveal that BHGJD exerts its protective effects through three interconnected mechanisms: 1) Nrf2/GPX4-mediated antioxidant defense preserving mitochondrial integrity and function; 2) PPARα/CPT1A-driven metabolic reprogramming favoring FAO; and 3) suppression of inflammatory lipid mediators as a synergistic outcome.

First, network pharmacology predicted enrichment in the regulation of ROS metabolic processes, which was supported by metabolomic findings showing GSH pathway enrichment and reduced lipid-peroxidation products. In parallel, (+)-catechin restored ATP levels and decreased ROS and Fe²^+^ accumulation in LPS-induced MLE-12 cells. Transmission electron microscopy further provided ultrastructural confirmation of mitochondrial protection. Collectively, these findings indicate that BHGJD is associated with activation of antioxidant defense and attenuation of ferroptosis-associated stress.

Given that *NFE2L2* ranked among the top 50 genes and that Nrf2 is recognized as the master regulator of the antioxidant response (20), together with GPX4 being a GSH metabolism–dependent protein (although *GPX4* was not included in the top 50 genes), we selected Nrf2 and its key downstream effector HO-1, as well as GPX4, as validation targets. Under basal conditions, Nrf2 is sequestered in the cytoplasm by Keap1 in an inactive state and rapidly degraded through the proteasomal system (21). In response to oxidative stress, Keap1 undergoes oxidation at reactive cysteine residues, leading to Keap1 inactivation, Nrf2 stabilization, and nuclear translocation, where it binds to antioxidant response elements to initiate transcription of more than 250 cytoprotective genes (22). These genes encode diverse proteins, including drug-metabolizing enzymes, HO-1, and key enzymes for GSH synthesis, thereby exerting protective effects (23). Immunohistochemical analysis revealed marked Nrf2 nuclear translocation in BHGJD-treated CLP lungs. However, Western blot analysis uncovered a critical dissociation: despite obvious HO-1 upregulation in the model group, GPX4 was paradoxically suppressed. BHGJD or (+)-catechin treatment restored GPX4 expression in both lung tissues and MLE-12 cells without elevating HO-1, indicating prioritization of GPX4 preservation through sustained Nrf2 activity rather than non-specific HO-1 induction.

Enrichment of the GSH metabolic pathway serves as the critical nexus linking Nrf2 to GPX4. Reduced GSH is the most abundant intracellular antioxidant and major detoxifying agent (24) and serves as the obligate cofactor for the GSH peroxidase family (25), which plays pivotal roles in combating oxidative stress and maintaining redox homeostasis. GPX4 is the only enzyme in the GPX family capable of directly reducing and detoxifying lipid hydroperoxides (26). Unlike cytosolic GPX1, which primarily detoxifies hydrogen peroxide, GPX4’s unique substrate specificity for membrane-embedded lipid hydroperoxides positions it as a key regulator of resistance to ferroptotic stress (27). The GPX4 upregulation after BHGJD treatment reduced levels of arachidonic acid-derived isoprostanes (5-F2t-IsoP and ent-11-epi-8-E2t-IsoP), which are reliable biomarkers for quantitative analysis of lipid peroxidation (28). *In vitro*, this upregulation was associated with normalization of intracellular Fe²^+^ levels in (+)-catechin-treated cells. This anti-ferroptotic effect preserved mitochondrial ultrastructure, as TEM showed that LPS-induced mitochondrial swelling, cristae disruption, and vacuolization were markedly attenuated by (+)-catechin. Structural preservation translated into functional recovery, reflected by restored cellular ATP. Notably, (+)-catechin also significantly reduced intracellular ROS levels. The attenuation of oxidative stress likely contributed to the stabilization of mitochondrial membranes, while improved mitochondrial integrity in turn limited excessive ROS production, forming a positive feedback loop that reinforced redox homeostasis.

Second, metabolomic detection of coordinated upregulation of multiple acylcarnitine species, encompassing short-chain (acetylcarnitine, butyrylcarnitine), medium-chain (octanoylcarnitine, dodecanoylcarnitine), and long-chain (hexadecenoylcarnitine, 3-hydroxyhexadecanoylcarnitine, etc.) carnitines. Acylcarnitine accumulation is not, by itself, definitive evidence of enhanced fatty acid β-oxidation. Depending on mitochondrial coupling and downstream oxidative capacity, it may also indicate incomplete β-oxidation or transient bottlenecks within the carnitine shuttle and distal oxidative pathways. However, in the context of concurrent pathway enrichment related to fatty acid transport and metabolism, along with restored expression of PPARα and CPT1A, improved ATP levels, and enhanced palmitate-supported oxygen consumption *in vitro*, these coordinated changes are interpreted as increased fatty acid utilization. PPARα is a central transcriptional regulator of lipid catabolism and promotes the expression of genes involved in fatty-acid transport and oxidation (29). CPT1A is the rate-limiting enzyme for mitochondrial import of long-chain fatty acids and catalyzes the formation of acylcarnitines from fatty acyl-CoA (30). Accordingly, increased PPARα and CPT1A expression, suggests that BHGJD facilitates mitochondrial fatty-acid utilization. These findings reveal that BHGJD may rescue the sepsis-induced bioenergetic crisis through activation of the PPARα/CPT1 axis to reshape energy metabolism.

Mitochondrial dysfunction leading to insufficient ATP generation constitutes a major contributor to organ failure in sepsis (31). Under stimulation by pro-inflammatory cytokines and hypoxic microenvironments, cellular metabolism undergoes a “Warburg-like effect” transition: from efficient mitochondrial oxidative phosphorylation (generating approximately 30–32 ATP per glucose molecule) to inefficient glycolysis (producing only 2 ATP per molecule) (32). This transition creates an ATP supply–demand mismatch, particularly detrimental to type II alveolar epithelial cells that depend on continuous ATP supply to maintain their functions through surfactant synthesis and Na^+^-K^+^-ATPase-mediated alveolar fluid clearance (33).

FAO in alveolar epithelial cells plays a crucial role in maintaining pulmonary cellular functional homeostasis (8). PPARα, the master transcriptional regulator of FAO, drives the expression of key β-oxidation enzymes, including carnitine palmitoyltransferase and acyl-coenyzme A (CoA) oxidase, upon activation (34). Network pharmacology predicted *PPARA* as a top 50 target, aligning with enrichment of the “fatty acid transport” pathway, and Western blot analysis confirmed PPARα upregulation at the protein level. The carnitine shuttle system serves as the obligatory pathway for long-chain fatty acid entry into mitochondria, with long-chain fatty acyl-CoA transported into mitochondria for β-oxidation via the carnitine palmitoyltransferase system composed of CPT1, CPT2, and carnitineacylcarnitine translocase (35). Accordingly, PPARα-mediated transcriptional activation of CPT1 accelerates the conversion of fatty acyl-CoA to acylcarnitines, thereby facilitating overall β-oxidation flux. Importantly, functional assessment using the Seahorse palmitate oxidation assay directly confirmed enhanced FAO capacity, providing metabolic evidence that the PPARα/CPT1A axis translated into increased mitochondrial FAO.

One study observed that PPARα expression is reduced in septic shock patients, with the degree of reduction correlating with disease severity; moreover, in sepsis models, *PPARα* gene-knockout mice exhibited lower survival rates than wild-type mice (36). PPARα agonists (such as fenofibrate) have demonstrated organ-protective effects in sepsis animal models. Pei et al. intervened in a CLP sepsis mouse model with fenofibrate, finding it could attenuate renal injury, reduce ROS levels, and increase the ATP/adenosine triphosphate (ADP) ratio (37). However, such protective effects have not been reported in lung injury models. As a multi-component TCM formula, BHGJD’s complex composition may achieve more comprehensive metabolic regulation than single PPARα agonists.

Third, metabolomics revealed widespread downregulation of arachidonic acid metabolites, including prostaglandins such as prostaglandin A1, 5,6β-dihydro prostaglandin I2, and 10-hydroxy-8E-decenoic acid, as well as lipid peroxidation products including 5-F_2_t-IsoP, ent-11-epi-8-E_2_t-IsoP, and 15-DiHETrE. These inflammatory lipid mediators play critical roles in the pathogenesis of sepsis-induced ALI. Their decline likely reflects a coordinated mechanism involving enhanced antioxidant defense and metabolic reprogramming, and may directly participate in anti-inflammatory effects.

Prostaglandins and isoprostanes represent two major classes of arachidonic acid metabolites with fundamentally different generation mechanisms. Prostaglandins are produced via cyclooxygenase (COX)-dependent enzymatic pathways (38), whereas isoprostanes arise from non-enzymatic ROS-driven lipid peroxidation (39). The synchronized downregulation of these two lipid mediator classes suggests that BHGJD may inhibit pathological arachidonic acid metabolism through dual mechanisms: attenuation of oxidative stress via the Nrf2/GPX4 antioxidant axis, and modulation of enzymatic inflammatory signaling pathways. In this study, the observed downregulation of p-mTOR may participate in this regulation. mTOR overactivation promotes HIF-1α accumulation and activity (40), which can transcriptionally induce COX-2 expression, forming a potential “mTOR/HIF-1α/COX-2” pro-inflammatory axis. Previous research showed that the mTOR inhibitor rapamycin can reduce PGE2 production in LPS-stimulated macrophages through COX-2 suppression (41).

mTOR is the core kinase for cellular nutrient–sensing and metabolic regulation (42), and plays a central role in the pathogenesis and development of sepsis-induced ALI by integrating energy metabolism, controlling cell fate, and regulating inflammatory responses (43). mTOR activation promotes glycolysis and inhibits mitochondrial oxidative phosphorylation via HIF-1α stabilization (44), directly suppresses PPARα activity, reducing ketone body generation (45), and promotes pulmonary epithelial cell inflammatory responses by suppressing autophagy and activating NF-κB (46), thereby fostering a vicious cycle of “pro-glycolysis-anti-FAO-pro-inflammation.” Our metabolomic analysis showed the mTOR pathway enrichment, and western blot demonstrated that the p-mTOR/mTOR ratio was significantly reduced in both lung tissues and MLE-12 cells following BHGJD or (+)-catechin treatment, indicating effective suppression of mTOR activation. Previous studies support mTOR as a rational sepsis therapeutic target (47–49). Importantly, the role of mTOR in ALI appears to be highly context-dependent (50). In inflammatory cells, mTOR activation can amplify TLR-driven inflammatory responses and inflammatory cell death, whereas inhibition of PI3K/AKT/mTOR signaling has been reported to promote autophagy and attenuate lung injury in septic models (51). In contrast, studies in epithelial and endothelial compartments indicate that basal mTOR activity may preserve barrier integrity and cell survival, and excessive mTOR inhibition may aggravate apoptosis or lung damage under certain conditions (52). This duality may explain why rapamycin shows protective effects in some ALI/sepsis models but deleterious effects in others. Therefore, in the current study, mTOR is better regarded as a candidate signaling hub potentially linked to redox balance, autophagy, and metabolic remodeling. Moreover, although interactions between mTOR and PPARα-dependent catabolic programs have been described in metabolic tissues, whether this crosstalk directly mediates the PPARα-related changes observed in septic lung remains to be established. BHGJD’s “gentle modulation” achieved through multi-component synergy may surpass single-agent “forceful blockade.” As the convergence point of oxidative stress, metabolism, and inflammation, mTOR orchestrates BHGJD’s multiplex protective mechanisms, and this multi-targeted regulation may represent the core advantage of TCM compound formulas.

This study has the following limitations requiring improvement in future work. First, while the current data link BHGJD-mediated protection to the Nrf2/GPX4 and PPARα/CPT1A pathways, this relationship is correlative rather than causal. We need to conduct loss-of-function experiments, including Nrf2^-^/^-^ models or targeted inhibitors, and incorporate mechanism-matched controls, such as NAC or fenofibrate. This approach will not only verify whether these pathways are indispensable but also better define the specific pharmacological profile of BHGJD. Second, the evaluation of mTOR signaling was preliminary and limited to the p-mTOR/mTOR ratio. Thus, its contribution to the observed effects requires further mechanistic validation. Third, (+)-catechin was used as a representative lung-exposed constituent for *in vitro* verification, and therefore the multi component interactions within BHGJD, as well as the pharmacokinetic relevance of the *in vitro* exposure concentration, remain to be clarified. Finally, research indicates that sepsis patients exhibit pronounced amino acid metabolic disturbances (53). Multiple amino acid metabolic pathways were enriched in our RNA-sequencing and untargeted metabolomics results, suggesting amino acid metabolic remodeling may also be one mechanism through which BHGJD exerts protective effects, pointing the way for future research.

## 5 Conclusion

This study demonstrates that BHGJD significantly alleviated sepsis-induced ALI and was associated with coordinated changes in antioxidant defense, lipid-related metabolism, and inflammatory lipid mediator profiles. The integrated data support a model in which Nrf2/GPX4-related redox regulation, PPARα/CPT1A-related metabolic remodeling, and altered mTOR signaling accompany the protective phenotype of BHGJD. These findings provide a mechanistically informative framework for understanding the actions of this classical formula in sepsis-induced ALI. Future investigations incorporating genetic knockout models, mitochondrial functional assessments, and amino acid detection will further strengthen causal relationships and facilitate clinical translation of this promising therapeutic approach for sepsis-induced ALI.

## Data Availability

The data presented in the study are deposited in the NCBI SRA repository, accession number SRR36514680.
