# Supplementary figures and images for: Baihe Gujin decoction ameliorates sepsis-induced acute lung injury through Nrf2/GPX4-mediated antioxidant defense and PPARα-driven metabolic reprogramming: a multi-omics investigation
Source: Front Immunol. 2026 Jun 29;17:1767881. doi: 10.3389/fimmu.2026.1767881 (PMC13357210; doi:10.3389/fimmu.2026.1767881)

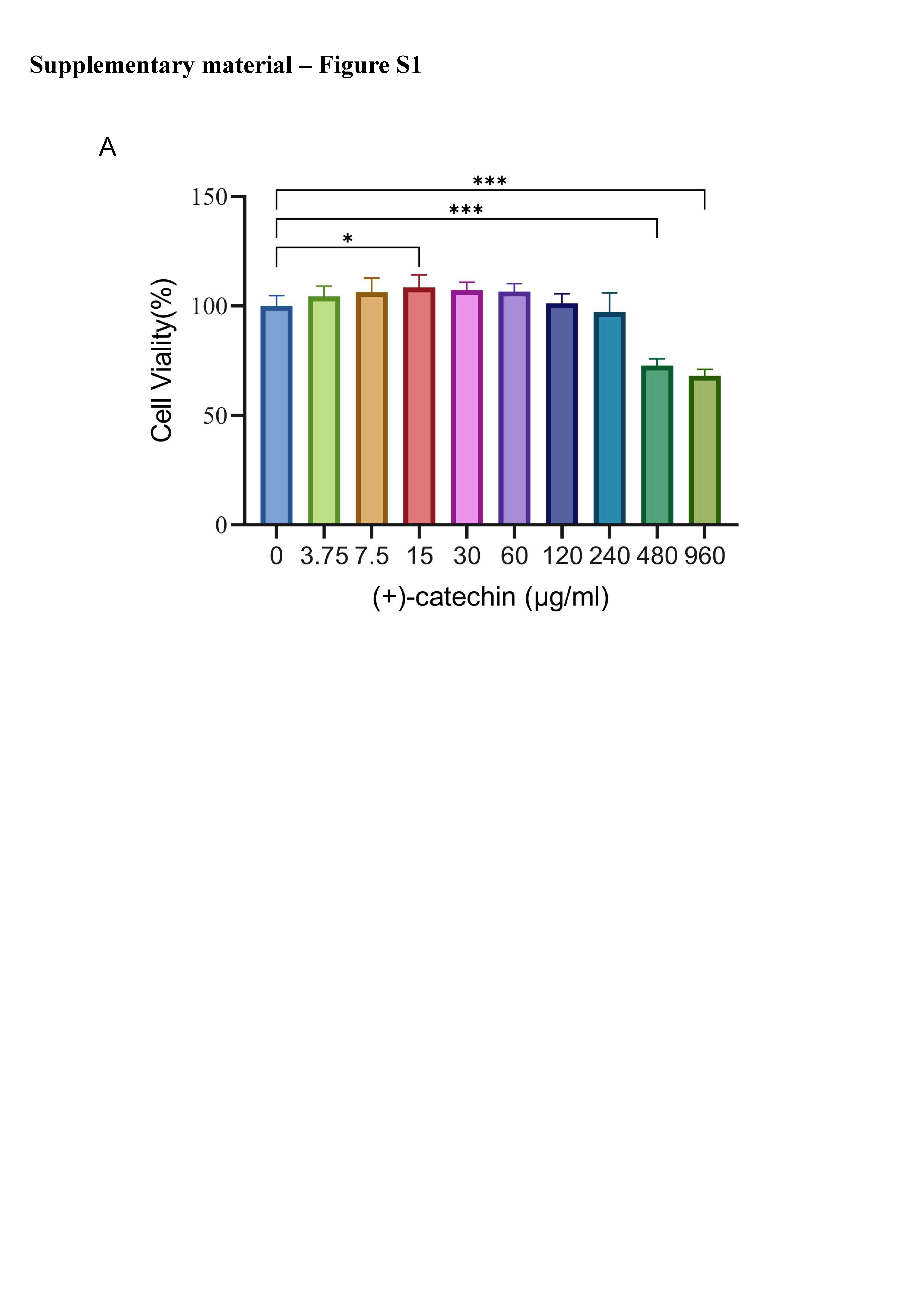

Supplement: Supplementary file 1 [file Image1.tif]
