# Supplementary material for: Baihe Gujin decoction ameliorates sepsis-induced acute lung injury through Nrf2/GPX4-mediated antioxidant defense and PPARα-driven metabolic reprogramming: a multi-omics investigation
Source: Front Immunol. 2026 Jun 29;17:1767881. doi: 10.3389/fimmu.2026.1767881 (PMC13357210; doi:10.3389/fimmu.2026.1767881)
Supplement: Supplementary file 2 [file Table1.pdf]

Table S1 The list of antibody.

Antibody	Company	Catalog Number
CPT1A	Immunoway	YM8449
Nrf2	Immunoway	YM8624
PPARα	Immunoway	YT3835
HO-1	Affinity Biosciences	AF5393
p-mTOR	Proteintech	67778-1-Ig
mTOR	Proteintech	66888-1-Ig
GPX4	Proteintech	67763-1-Ig
β-actin	Proteintech	66009-1-Ig
Goat Anti-Rabbit IgG(H+L), HRP Conjugated	Epizyme	LF102
Goat Anti-Rabbit IgG (H+L), BF488 Conjugated	Epizyme	LF108
Goat Anti-Rabbit IgG (H+L), GF555 Conjugated	Epizyme	LF110
