# Supplementary material for: Baihe Gujin decoction ameliorates sepsis-induced acute lung injury through Nrf2/GPX4-mediated antioxidant defense and PPARα-driven metabolic reprogramming: a multi-omics investigation
Source: Front Immunol. 2026 Jun 29;17:1767881. doi: 10.3389/fimmu.2026.1767881 (PMC13357210; doi:10.3389/fimmu.2026.1767881)
Supplement: Supplementary file 3 [file Table2.pdf]

Table S2 The list of compounds identified in BHGJD.

Code	Formula	Experimental m/z	RT [min]	Compounds	ppm	Reference Ion	classification
1	C8 H16 O2	145.1224	0.149	Octylic acid	0.8	$[M+H]^{+1}$	organic acid
2	C12 H22 O11	387.1148	0.612	D-(+)-Maltose	4.49	$[M-H]^{-1}$	saccharide
3	C6 H14 N2 O2	147.1128	0.971	L-Lysine	0.16	$[M+H]^{+1}$	amino acid
4	C6 H9 N3 O2	156.0768	1.008	L-Histidine	0.48	$[M+H]^{+1}$	amino acid
5	C5 H13 N O	104.1074	1.158	Choline	4.16	$[M+H]^{+1}$	alkaloid
6	C6 H14 N4 O2	175.119	1.174	DL-Arginine	0.11	$[M+H]^{+1}$	amino acid
7	C5 H9 N O2	116.0708	1.186	Proline	2.03	$[M+H]^{+1}$	amino acid
8	C3 H7 N O3	104.0341	1.232	L-Serine	1.91	$[M-H]^{-1}$	amino acid
9	C5 H10 N2 O3	145.0608	1.233	D-(-)-Glutamine	-7.13	$[M-H]^{-1}$	amino acid
10	C4 H8 N2 O3	131.0451	1.234	Asparagine	-8.52	$[M-H]^{-1}$	amino acid
11	C4 H9 N O3	118.0498	1.244	Threonine	-9.95	$[M-H]^{-1}$	amino acid
12	C24 H42 O21	689.2107	1.249	Lupeose	-0.77	$[M+H]^{+1}$	saccharide
13	C4 H9 N O3	120.0657	1.261	L-Threonine	1.64	$[M+H]^{+1}$	amino acid
14	C12 H22 O11	343.1233	1.261	Sucrose	-1.44	$[M+H]^{+1}$	saccharide
15	C7 H7 N O2	138.055	1.313	Trigonelline	-0.06	$[M+H]^{+1}$	alkaloid
16	C9 H11 N O3	182.0811	1.315	DL-TYROSINE	-0.21	$[M+H]^{+1}$	amino acid
17	C18 H32 O16	487.1656	1.32	D-Raffinose	-0.32	$[M+H-H2O]^{+1}$	saccharide
18	C5 H9 N O2	116.0709	1.323	Pterolactam	2.42	$[M+H]^{+1}$	others
19	C7 H13 N O2	144.102	1.371	Stachydrine	0.38	$[M+H]^{+1}$	alkaloid
20	C5 H11 N O2	118.0865	1.402	DL-Isovaline	2.11	$[M+H]^{+1}$	amino acid
21	C15 H18 O8	327.1074	1.446	BILOBALIDE	-0.16	$[M+H]^{+1}$	terpenoid
22	C15 H22 O10	380.1548	1.495	Catalpol	-1.06	$[M+NH4]^{+1}$	terpenoid
23	C9 H12 N2 O6	243.0621	1.508	Uridine	-0.64	$[M-H]^{-1}$	nucleotide
24	C4 H9 N O2	104.071	1.589	4-aminobutyric acid	3.5	$[M+H]^{+1}$	organic acid
25	C4 H7 N O4	134.0449	1.683	L-(+)-Aspartic acid	0.92	$[M+H]^{+1}$	amino acid
26	C4 H4 O4	115.0026	1.835	Fumaric acid	-9.05	$[M-H]^{-1}$	organic acid
27	C5 H7 N O3	130.0499	1.932	D-(+)-Pyroglutamic Acid	0.89	$[M+H]^{+1}$	amino acid
28	C6 H13 N O2	132.102	1.955	L-(+)-Leucine	0.99	$[M+H]^{+1}$	amino acid
29	C6 H5 N O2	124.0395	2.017	Nicotinic acid	1.69	$[M+H]^{+1}$	vitamin
30	C10 H13 N5 O4	268.1039	2.076	Adenosine	-0.33	$[M+H]^{+1}$	nucleotide
31	C6 H13 N O2	132.102	2.092	Isoleucine	0.53	$[M+H]^{+1}$	amino acid
32	C10 H13 N5 O5	284.0989	2.464	Guanosine	-0.13	$[M+H]^{+1}$	nucleotide
33	C9 H10 O2	151.0755	2.565	3，4-DIMETHYLBENZOIC ACID	0.79	$[M+H]^{+1}$	organic acid
34	C4 H6 O5	133.0132	2.603	DL-Malic acid	-7.59	$[M-H]^{-1}$	organic acid
35	C9 H14 O5	185.081	2.607	Rehmaglutin A	0.53	$[M+H-H2O]^{+1}$	terpenoid
36	C11 H12 N2 O2	205.0973	2.711	DL-Tryptophan	0.59	$[M+H]^{+1}$	amino acid
37	C17 H20 N4 O6	415.1004	2.734	(−)-Riboflavin	-3.16	$[M+K]^{+1}$	vitamin
38	C5 H5 N5	136.0619	2.751	Adenine	1.24	$[M+H]^{+1}$	nucleotide
39	C9 H8 O3	165.0547	3.015	p-coumaric acid	0.36	$[M+H]^{+1}$	coumarin
40	C9 H11 N O2	166.0862	3.166	L-Phenylalanine	-0.08	$[M+H]^{+1}$	amino acid
41	C4 H4 N2 O2	113.0347	3.178	Uracil	1.56	$[M+H]^{+1}$	nucleotide
42	C10 H14 N2 O5	284.124	3.264	Thymidine	-0.64	$[M+ACN+H]^{+1}$	nucleotide
43	C9 H8 O3	165.0546	3.473	4-Coumaric acid	-0.25	$[M+H]^{+1}$	coumarin
44	C5 H7 N O3	130.0499	3.58	L-Pyroglutamic acid	0.56	$[M+H]^{+1}$	amino acid
45	C5 H6 N2 O2	127.0503	3.603	5-methyluracil	0.96	$[M+H]^{+1}$	nucleotide
46	C5 H6 N2 O2	127.0503	3.82	Thymine	1.15	$[M+H]^{+1}$	nucleotide
47	C21 H23 N O6	386.1582	4.062	3-Desmethylcolchicine	-4.18	$[M+H]^{+1}$	alkaloid
48	C8 H9 N	120.081	4.142	2-Methyl-5-vinylpyridine	1.96	$[M+H]^{+1}$	others
49	C7 H8 O	109.0651	4.22	benzenemethanol	3.17	$[M+H]^{+1}$	others
50	C5 H9 N O4	148.0604	4.3	L-Glutamic acid	-0.06	$[M+H]^{+1}$	amino acid

51	C14 H18 O4	251.1277	4.745	Dipropyl phthalate	-0.24	$[M+H]^{+1}$	others
52	C15 H24 O10	382.1704	4.932	Dihydrocatalpol	-0.89	$[M+NH4]^{+1}$	terpenoid
53	C7 H8 O2	107.0495	4.94	3-Methoxyphenol	1.3	$[M+H-H2O]^{+1}$	phenol
54	C10 H16 O3	185.1173	5.165	Rehmapicrogenin	0.4	$[M+H]^{+1}$	terpenoid
55	C10 H10 O4	195.0652	5.464	Dimethyl phthalate	0.24	$[M+H]^{+1}$	phenol
56	C12 H12 O3	205.086	5.784	3-Butylidene-7-hydroxyphthalide	0.25	$[M+H]^{+1}$	coumarin
57	C4 H6 O4	119.034	5.982	Succinic acid	0.81	$[M+H]^{+1}$	organic acid
58	C5 H8 O2	101.0601	6.511	Angelic acid	3.83	$[M+H]^{+1}$	organic acid
59	C12 H14 O5	239.0913	6.593	(E)-3，4，5-Trimethoxycinnamic acid	-0.47	$[M+H]^{+1}$	coumarin
60	C8 H10 N2 O	151.0866	6.821	3-METHYLPHENYLUREA	0.03	$[M+H]^{+1}$	others
61	C16 H23 N O6	326.1597	7.107	Crotaline	-0.45	$[M+H]^{+1}$	alkaloid
62	C7 H6 O2	123.0443	7.182	4-Hydroxybenzaldehyde	1.97	$[M+H]^{+1}$	phenol
63	C4 H8 N2 O3	133.0609	7.272	L-(−)-Asparagine	1.21	$[M+H]^{+1}$	amino acid
64	C18 H19 N O4	314.1385	7.274	Moupinamide	-0.63	$[M+H]^{+1}$	alkaloid
65	C7 H14 O	115.1121	7.282	n-heptanal	2.79	$[M+H]^{+1}$	others
66	C28 H32 O16	625.174	7.426	Complanatuside	-3.78	$[M+H]^{+1}$	flavonoid
67	C15 H20 O8	329.1228	7.492	Paeonoside	-0.98	$[M+H]^{+1}$	glycoside
68	C9 H7 N O	146.0599	7.576	7-Hydroxyisoquinoline	-0.72	$[M+H]^{+1}$	others
69	C14 H14 O4	247.096	7.878	Marmesin	-2.09	$[M+H]^{+1}$	coumarin
70	C8 H8 O3	153.0546	7.936	ANISIC ACID	-0.16	$[M+H]^{+1}$	organic acid
71	C10 H14 O4	181.0858	7.963	paeonilactone A	2.09	$[M+H]^{+1}$	terpenoid
72	C16 H24 O10	359.1334	8.019	6-O-methylcatalpol	-0.34	$[M+H-H2O]^{+1}$	terpenoid
73	C9 H8 O2	149.0597	8.083	Cinnamic acid	0.22	$[M+H]^{+1}$	coumarin
74	C22 H23 N O7	414.1552	8.289	Tusscapine	1.1	$[M+H]^{+1}$	alkaloid
75	C13 H16 N2 O	217.1338	8.318	Tetrahydroharmine	1.37	$[M+H]^{+1}$	alkaloid
76	C15 H14 O6	291.0861	8.402	Cianidanol	-0.77	$[M+H]^{+1}$	flavonoid
77	C9 H6 O3	163.0387	8.586	6-Hydroxycoumarin	-1.41	$[M+H]^{+1}$	coumarin
78	C8 H10 O3	155.0703	8.791	4-(Methoxymethoxy)phenol	0.47	$[M+H]^{+1}$	phenol
79	C15 H20 O7	354.1553	9.031	NIVALENOL	1.52	$[M+ACN+H]^{+1}$	terpenoid
80	C12 H12 O3	205.086	9.57	Senkyunolide C	0.61	$[M+H]^{+1}$	phthalide
81	C15 H12 O6	289.0707	9.814	(+/-)-Eriodictyol	0.05	$[M+H]^{+1}$	flavonoid
82	C15 H14 O6	291.0863	9.838	Epicatechin	-0.04	$[M+H]^{+1}$	flavonoid
83	C16 H14 O6	303.0864	10.18	Hesperetin	0.15	$[M+H]^{+1}$	flavonoid
84	C17 H18 O5	303.1228	10.263	Isomucronulatol	1.95	$[M+H]^{+1}$	flavonoid
85	C8 H8 O2	137.0598	10.303	2-Phenylacetic acid	0.8	$[M+H]^{+1}$	organic acid
86	C11 H12 O5	225.0756	10.307	Sinapinic acid	-0.25	$[M+H]^{+1}$	phenol
87	C6 H12 O2	117.0914	10.751	Ethylisobutyrate	3.13	$[M+H]^{+1}$	others
88	C8 H8 O4	169.0496	10.802	Vanillic acid	0.33	$[M+H]^{+1}$	organic acid
89	C20 H20 O4	347.1236	10.811	Glabridin	1.6	$[M+H]^{+1}$	flavonoid
90	C10 H18 O	155.1431	10.881	CITRONELLAL	0.06	$[M+H]^{+1}$	terpenoid
91	C6 H7 N O	110.0604	11.247	5-Hydroxy-2-methylpyridine	3.31	$[M+H]^{+1}$	others
92	C30 H46 O4	471.354	11.28	18-β-Glycyrrhetinic acid	4.24	$[M+H]^{+1}$	terpenoid
93	C9 H10 O5	181.0495	11.365	Ethyl gallate	0.05	$[M+H-H2O]^{+1}$	phenol

94	C10 H18 O	155.1432	11.534	Neoisopulegol	0.95	$[M+H]^{+1}$	phenol
95	C20 H28 O12	461.1649	11.613	Paeonolide	-0.25	$[M+H]^{+1}$	glycoside
96	C17 H34 O2	271.2629	11.812	Heptadecanoic acid	-0.8	$[M+H]^{+1}$	fatty acid
97	C29 H36 O15	625.2119	11.814	verbascoside	-1.31	$[M+H]^{+1}$	glycoside
98	C22 H25 N O6	400.1752	11.817	(±)-Colchicine	-0.58	$[M+H]^{+1}$	alkaloid
99	C17 H20 O10	385.1134	11.859	Eleutheroside B1	1.17	$[M+H]^{+1}$	coumarin
100	C10 H18 O	155.1431	11.901	Dihydrocarveol	0.26	$[M+H]^{+1}$	others
101	C10 H8 O4	193.0497	12.206	Gelseminic acid	0.61	$[M+H]^{+1}$	organic acid
102	C22 H30 O13	485.1654	12.207	Sibirioside B	-0.02	$[M+H-H2O]^{+1}$	phenylpropanoid
103	C15 H24 O8	333.1543	12.232	Ningpogoside A	-0.28	$[M+H]^{+1}$	terpenoid
104	C20 H23 N O4	342.17	12.273	Lauroscholtzine	0.08	$[M+H]^{+1}$	alkaloid
105	C20 H18 O6	355.1177	12.415	Licoisoflavanone	0.25	$[M+H]^{+1}$	flavonoid
106	C29 H36 O15	625.2122	12.443	Isoverbascoside	-0.76	$[M+H]^{+1}$	glycoside
107	C27 H30 O15	595.1653	12.588	Nicotiflorin	-0.68	$[M+H]^{+1}$	flavonoid
108	C23 H28 O11	481.1704	12.591	Paeoniflorin	-0.02	$[M+H]^{+1}$	terpenoid
109	C17 H18 O6	319.1175	12.618	paeoniflorigenone	-0.3	$[M+H]^{+1}$	terpenoid
110	C10 H10 O3	179.0702	12.817	4-Methoxycinnamic acid	-0.62	$[M+H]^{+1}$	coumarin
111	C15 H28 O2	241.2158	12.915	Cyclopentadecanolide	-1.54	$[M+H]^{+1}$	others
112	C18 H32 O2	281.247	12.994	Bovinic acid	-1.67	$[M+H]^{+1}$	organic acid
113	C26 H28 O14	565.1543	13.176	Schaftoside	-1.57	$[M+H]^{+1}$	flavonoid
114	C8 H8 O3	153.0547	13.203	Vanillin	0.54	$[M+H]^{+1}$	phenol
115	C15 H12 O5	273.0756	13.332	(±)-Naringenin	-0.69	$[M+H]^{+1}$	flavonoid
116	C27 H43 N O3	430.3314	13.378	Sipeimine	-0.35	$[M+H]^{+1}$	alkaloid
117	C23 H28 O11	519.1263	13.471	Albiflorin	4.73	$[M+H]^{+1}$	terpenoid
118	C8 H8 O2	137.0597	13.516	4-Hydroxyacetophenone	0.24	$[M+H]^{+1}$	phenol
119	C23 H28 O12	497.1664	13.69	oxypaeoniflorin	2.01	$[M+H]^{+1}$	terpenoid
120	C10 H10 O4	195.0653	13.759	(E)-Ferulic acid	0.32	$[M+H]^{+1}$	organic acid
121	C16 H22 O8	343.1386	13.883	Abietin	-0.39	$[M+H]^{+1}$	phenylpropanoid
122	C9 H8 O3	147.0441	14.074	2-Hydroxycinnamic acid	3.57	$[M+H]^{+1}$	coumarin
123	C16 H12 O5	285.0761	14.839	Calycosin	1.21	$[M+H]^{+1}$	flavonoid
124	C10 H14	135.1169	15.02	P-CYMENE	0.53	$[M+H]^{+1}$	terpenoid
125	C33 H55 N O8	594.4	15.028	Zhebeininosinoside	-0.03	$[M+H]^{+1}$	alkaloid
126	C9 H14 O	121.1014	15.032	2，4-Nonadien^{-1}-al	1.19	$[M+H-H2O]^{+1}$	others
127	C19 H18 O6	384.1441	15.038	Methylophiopogonanone A	0.85	$[M+ACN+H]^{+1}$	flavonoid
128	C17 H20 O9	369.1182	15.082	5-Feruoylquinic acid	0.51	$[M+H]^{+1}$	organic acid
129	C9 H10 O3	167.0703	15.157	Paeonol	0.07	$[M+H]^{+1}$	phenol
130	C8 H14 O2	143.1067	15.323	3E-Hexenyl acetate	0.02	$[M+H]^{+1}$	others
131	C21 H20 O9	417.1179	15.329	Daidzin	-0.29	$[M+H]^{+1}$	flavonoid
132	C16 H14 O4	271.0964	15.339	Imperatorin	-0.3	$[M+H]^{+1}$	coumarin
133	C15 H12 O5	273.0756	15.396	Naringenin	-0.47	$[M+H]^{+1}$	flavonoid
134	C9 H8 O4	163.0389	15.542	Caffeic acid	-0.3	$[M+H-H2O]^{+1}$	organic acid
135	C18 H32 O3	297.2417	15.639	(+)-beta-Dimorphecolic acid	-2.44	$[M+H]^{+1}$	fatty acid
136	C27 H41 N O3	428.3159	15.932	peimisine	-0.08	$[M+H]^{+1}$	alkaloid
137	C10 H10 O4	195.0652	15.993	Ferulic acid	0	$[M+H]^{+1}$	organic acid
138	C7 H6 O2	105.0338	16.08	Benzoic acid	1.78	$[M+H-H2O]^{+1}$	organic acid
139	C16 H12 O5	285.0752	16.153	Prunetin	-1.95	$[M+H]^{+1}$	flavonoid
140	C15 H12 O4	257.0806	16.23	Isoliquiritigenin	-0.93	$[M+H]^{+1}$	flavonoid
141	C18 H32 O16	505.1782	16.236	Maltotriose	3.79	$[M+H]^{+1}$	saccharide
142	C27 H32 O14	581.186	16.262	Naringin	-0.84	$[M+H]^{+1}$	flavonoid

143	C27 H30 O16	611.1583	16.327	Rutin	-3.83	$[M+H]^{+1}$	flavonoid
144	C16 H18 O9	355.1029	16.557	Chlorogenic acid	1.54	$[M+H]^{+1}$	organic acid
145	C13 H22 O2	211.1691	16.705	Geranyl propionate	-0.54	$[M+H]^{+1}$	others
146	C27 H45 N O3	432.3471	16.76	Peimine	-0.26	$[M+H]^{+1}$	alkaloid
147	C27 H41 N O2	412.3229	16.793	Cyclopamine	4.62	$[M+H]^{+1}$	alkaloid
148	C16 H14 O5	287.0912	16.83	(-)-Nissolin	-0.75	$[M+H]^{+1}$	flavonoid
149	C33 H51 N O7	574.3733	16.919	Cycloposine	-0.94	$[M+H]^{+1}$	alkaloid
150	C27 H43 N O2	414.336	16.995	Ziebeimine	-1.66	$[M+H]^{+1}$	alkaloid
151	C8 H10	107.0859	17.045	p-Xylene	3.44	$[M+H]^{+1}$	others
152	C7 H6 O5	169.0135	17.067	Gallic acid	-4.55	$[M-H]^{-1}$	phenol
153	C27 H43 N O2	414.3363	17.13	Delavinone	-0.81	$[M+H]^{+1}$	alkaloid
154	C8 H10 O	123.0807	17.234	Phenylethyl alcohol	2.02	$[M+H]^{+1}$	others
155	C10 H14 O	151.1117	17.527	CARVACROL	-0.32	$[M+H]^{+1}$	phenol
156	C27 H43 N O3	430.3317	17.613	peiminine	0.29	$[M+H]^{+1}$	alkaloid
157	C6 H14 O6	183.0863	17.809	Mannitol	-0.18	$[M+H]^{+1}$	saccharide
158	C18 H32 O2	281.2475	17.886	Linoelaidic Acid	0.02	$[M+H]^{+1}$	fatty acid
159	C33 H55 N O8	594.3996	18.106	Hupehemonoside	-0.75	$[M+H]^{+1}$	alkaloid
160	C14 H16 O6	281.1018	18.145	Zhebeiresinol	-0.63	$[M+H]^{+1}$	others
161	C27 H39 N O2	410.305	18.197	Eratramine	-0.96	$[M+H]^{+1}$	flavonoid
162	C15 H12 O4	257.0809	18.297	Emodinanthrone	0.38	$[M+H]^{+1}$	phenol
163	C20 H30 O2	303.2318	18.588	Communic Acid	-0.35	$[M+H]^{+1}$	organic acid
164	C10 H16	137.1325	18.722	D-Limonene	0.11	$[M+H]^{+1}$	terpenoid
165	C11 H12 O4	209.081	18.729	Ethyl caffeate	0.69	$[M+H]^{+1}$	phenol
166	C32 H48 O5	257.1858	18.758	Glycyrrhetic acid acetate	3.88	[M+2H]+2	terpenoid
167	C10 H12 O	149.0962	18.929	(Z)-Anethole	0.67	$[M+H]^{+1}$	phenylpropanoid
168	C12 H14 O2	191.1066	19.028	Butylphthalide	-0.07	$[M+H]^{+1}$	others
169	C16 H26 O8	347.17	19.098	Rehmapicroside	-0.27	$[M+H]^{+1}$	terpenoid
170	C10 H10 O4	177.0546	19.128	Isoferulic acid	0.19	$[M+H-H2O]^{+1}$	organic acid
171	C10 H18 O	155.1432	19.2	(L)-alpha-Terpineol	0.85	$[M+H]^{+1}$	terpenoid
172	C21 H22 O10	435.1287	19.269	Isoengeletin	0.2	$[M+H]^{+1}$	flavonoid
173	C30 H32 O15	633.1812	19.291	Galloylalbiflorin	-0.39	$[M+H]^{+1}$	phenol
174	C15 H12 O5	273.0757	19.458	Naringeninchalcone	-0.36	$[M+H]^{+1}$	flavonoid
175	C21 H20 O10	433.1131	19.795	Vitexin	0.33	$[M+H]^{+1}$	flavonoid
176	C16 H12 O6	301.0704	19.906	Dinatin	-0.77	$[M+H]^{+1}$	flavonoid
177	C17 H14 O6	315.0867	20.194	Kumatakenin	1.33	$[M+H]^{+1}$	flavonoid
178	C15 H12 O4	257.0808	20.319	Pinocembrin	-0.1	$[M+H]^{+1}$	flavonoid
179	C9 H8 O3	165.0546	20.604	o-Coumaric acid	0.13	$[M+H]^{+1}$	coumarin
180	C14 H14 O4	247.094	20.638	Nodakenetin	4.06	$[M+H]^{+1}$	coumarin
181	C27 H45 N O3	432.3472	20.704	isobaimonidine	0.02	$[M+H]^{+1}$	alkaloid
182	C12 H16 O4	207.1016	20.726	Senkyunolide H	4.45	$[M+H]^{+1}$	phthalide
183	C12 H14 O4	223.0964	20.768	Senkyunolide D	-0.29	$[M+H]^{+1}$	phthalide
184	C17 H18 O7	335.1126	20.8	Byakangelicin	0.06	$[M+H]^{+1}$	coumarin
185	C18 H34 O2	283.263	20.98	Oleic acid	-0.45	$[M+H]^{+1}$	fatty acid
186	C36 H48 O19	802.3119	20.989	Angoroside C	2.65	$[M+H]^{+1}$	phenylpropanoid
187	C16 H18 O8	339.1073	21.064	Gerberinside	-0.43	$[M+H]^{+1}$	coumarin
188	C17 H16 O5	301.1067	21.145	Melilotocarpan A	-1.2	$[M+H]^{+1}$	terpenoid
189	C11 H16 O	165.1274	21.177	O-Methylthymol	-0.26	$[M+H]^{+1}$	phenol
190	C31 H40 O15	653.2453	21.229	Cistanoside D	1.99	$[M+H]^{+1}$	phenylpropanoid
191	C33 H53 N O7	576.3889	21.236	Isorubijervine	-1.09	$[M+H]^{+1}$	alkaloid
192	C15 H16 O6	293.1017	21.469	Angelicain	-0.85	$[M+H]^{+1}$	phthalide
193	C11 H14 O2	179.1067	21.669	N-Butyl benzoate	0.27	$[M+H]^{+1}$	phenol
194	C9 H6 O3	163.039	21.914	Umbelliferone	0.1	$[M+H]^{+1}$	coumarin
195	C12 H12 O2	189.091	22.021	(Z)-3-butylidenephthalide	0.15	$[M+H]^{+1}$	coumarin

196	C16 H12 O4	269.0806	22.062	Formononetin	-0.78	$[M+H]^{+1}$	flavonoid
197	C22 H22 O9	431.1339	22.064	Formononetin glucoside	0.52	$[M+H]^{+1}$	flavonoid
198	C21 H22 O9	419.1335	22.302	Liquiritin	-0.35	$[M+H]^{+1}$	flavonoid
199	C20 H22 O6	341.1384	22.566	(-)-Pinoresinol	4.81	$[M+H]^{+1}$	lignan
200	C20 H18 O4	323.128	22.667	Glabrene	0.54	$[M+H]^{+1}$	flavonoid
201	C17 H34 O2	271.2631	22.712	METHYL PALMITATE	-0.35	$[M+H]^{+1}$	others
202	C15 H20 O2	233.1537	22.749	Costunolide	0.28	$[M+H]^{+1}$	terpenoid
203	C30 H44 O4	469.3308	22.925	3-Oxoglycyrrhetinate	-0.97	$[M+H]^{+1}$	terpenoid
204	C20 H30 O2	303.2333	23.121	Isopimaric acid A	4.9	$[M+H]^{+1}$	terpenoid
205	C15 H10 O4	255.0651	23.232	7，4'-Dihydroxyflavone	-0.36	$[M+H]^{+1}$	flavonoid
206	C28 H47 N O2	430.3685	23.259	Ningpeisne	1.19	$[M+H]^{+1}$	alkaloid
207	C11 H18 O2	183.1381	23.47	Geranyl formate	0.49	$[M+H]^{+1}$	fatty acid
208	C31 H40 O15	653.2421	23.505	martynoside	-2.96	$[M+H]^{+1}$	phenylpropanoid
209	C10 H8 O3	177.0546	23.717	6-methoxycoumarin	0.03	$[M+H]^{+1}$	coumarin
210	C25 H24 O13	533.1293	23.791	Calycosin-7-O-β-D-(6"-O-malonate)-glucoside	0.69	$[M+H]^{+1}$	flavonoid
211	C27 H43 N O3	430.3317	23.807	verticinone	0.22	$[M+H]^{+1}$	alkaloid
212	C15 H12 O4	257.0809	23.869	Liquiritigenin	0.14	$[M+H]^{+1}$	flavonoid
213	C30 H48 O7	521.3471	23.966	Platycodigenin	-0.28	$[M+H]^{+1}$	saponin
214	C12 H14 O3	207.1017	24.34	4-hydroxy3-butylphthalide	0.63	$[M+H]^{+1}$	phenol
215	C10 H16	137.1326	25.116	Terpinolen	0.56	$[M+H]^{+1}$	terpenoid
216	C27 H45 N O2	416.3521	25.356	Delarine	-0.39	$[M+H]^{+1}$	alkaloid
217	C30 H32 O13	601.1914	25.373	Mudanpioside C	-0.37	$[M+H]^{+1}$	terpenoid
218	C17 H16 O5	301.1071	25.402	phellopterin	0.12	$[M+H]^{+1}$	coumarin
219	C5 H8 O4	133.0496	25.586	Glutaric acid	0.77	$[M+H]^{+1}$	organic acid
220	C16 H20 O9	357.118	25.596	Dicaffeoylquinic acid	-0.08	$[M+H]^{+1}$	organic acid
221	C16 H12 O5	285.0757	25.597	Wogonin	-0.02	$[M+H]^{+1}$	flavonoid
222	C10 H18 O	155.143	25.809	Alpha-Fenchol	-0.14	$[M+H]^{+1}$	terpenoid
223	C16 H14 O4	271.0965	25.906	Echinatin	0.15	$[M+H]^{+1}$	flavonoid
224	C24 H30 O11	539.1772	25.937	Harpagoside	5.15	$[M-H]^{-1}$	terpenoid
225	C27 H42 O3	415.3209	26.004	Diosgenin	0.61	$[M+H]^{+1}$	steroid
226	C8 H8 O	121.065	26.243	Acetophenone	1.63	$[M+H]^{+1}$	others
227	C8 H8 O2	137.0598	26.355	Methylbenzoate	0.57	$[M+H]^{+1}$	others
228	C25 H32 O13	539.1776	26.814	6″-O-feruloylharpagid	1.11	$[M-H]^{-1}$	flavonoid
229	C39 H62 O12	723.4296	26.96	Prosapogenin B	-2.52	$[M+H]^{+1}$	saponin
230	C57 H92 O28	1225.589	27.29	Platycodin D	3.43	$[M+H]^{+1}$	saponin
231	C15 H26 O	223.2056	28.442	(+)-Cedrol	-0.27	$[M+H]^{+1}$	terpenoid
232	C14 H12 O4	245.0809	28.532	Graveolone	0.39	$[M+H]^{+1}$	flavonoid
233	C23 H26 O10	463.1597	28.549	Lactiflorin	-0.44	$[M+H]^{+1}$	terpenoid
234	C9 H16 O2	157.1224	28.643	8-NONENOIC ACID	0.35	$[M+H]^{+1}$	fatty acid
235	C9 H6 O3	161.0239	28.714	4-Hydroxycoumarin	-3.03	$[M-H]^{-1}$	coumarin
236	C17 H27 N O3	294.2065	28.811	Nonivamide	0.38	$[M+H]^{+1}$	alkaloid
237	C44 H70 O16	855.4765	29.001	Ophiopogonin D	3.32	$[M+H]^{+1}$	saponin
238	C59 H94 O29	1267.592	29.116	Platycodin A	-2.63	$[M+H]^{+1}$	saponin
239	C30 H32 O12	585.1963	29.188	Benzoylpaeoniflorin	-0.62	$[M+H]^{+1}$	terpenoid
240	C16 H16 O5	289.1071	29.287	Angelicone	0.13	$[M+H]^{+1}$	coumarin
241	C8 H10 O3	155.0703	29.389	Hydroxytyrosol	0.17	$[M+H]^{+1}$	phenol
242	C11 H14 O2	179.1066	29.429	Methylisoeugenol	-0.41	$[M+H]^{+1}$	phenol
243	C30 H44 O4	469.3312	29.438	Glabrolide	-0.13	$[M+H]^{+1}$	terpenoid

244	C41 H32 O26	941.1216	29.646	1，2，3，4，6-O-Pentagalloylglucose	-4.06	$[M+H]^{+1}$	phenol
245	C42 H62 O17	839.4053	30.665	LICORICESAPONIN G2	-0.77	$[M+H]^{+1}$	saponin
246	C8 H16 O2	145.1223	31.309	Caprylic Acid	0.06	$[M+H]^{+1}$	organic acid
247	C11 H16 O2	181.1222	31.791	(Z，S)-jasmololone	-0.63	$[M+H]^{+1}$	others
248	C21 H20 O6	369.1326	32.513	Glycycoumarin	-1.81	$[M+H]^{+1}$	coumarin
249	C22 H22 O10	447.129	32.563	Trifolirhizin	0.88	$[M+H]^{+1}$	flavonoid
250	C9 H14 O3	171.1015	32.676	Buergerinin F	-0.23	$[M+H]^{+1}$	terpenoid
251	C12 H18 O2	195.1379	32.921	Sedanolide	-0.17	$[M+H]^{+1}$	phthalide
252	C18 H34 O5	348.2741	32.935	Pinellic acid	3.88	$[M+H]^{+1}$	organic acid
253	C18 H32 O4	295.2266	32.944	(9E)-octadecenedioic acid	4.25	$[M+H]^{+1}$	fatty acid
254	C30 H46 O3	455.3519	33.571	Liquidambaric acid	-0.16	$[M+H]^{+1}$	organic acid
255	C34 H46 O18	743.273	34.018	syringaresinol	-3.61	$[M+H]^{+1}$	lignan
256	C39 H62 O12	723.4282	34.202	Prosapogenin A	-4.49	$[M+H]^{+1}$	saponin
257	C14 H26 O2	227.2003	34.617	Tsuzuic acid	-0.99	$[M+H]^{+1}$	fatty acid
258	C8 H8 O5	185.0443	35.678	Methyl gallate	-0.67	$[M+H]^{+1}$	phenol
259	C13 H24 N2 O	225.1961	36.214	1，3-Dicyclohexylurea	-0.22	$[M+H]^{+1}$	others
260	C16 H14 O4	271.0964	36.777	Alloisoimperatorin	-0.41	$[M+H]^{+1}$	coumarin
261	C18 H36 O	269.2837	38.196	Oleyl Alcohol	-0.75	$[M+H]^{+1}$	others
262	C10 H12 O2	165.091	38.232	Eugenol	-0.14	$[M+H]^{+1}$	phenol
263	C12 H20 O2	197.1537	38.584	Tuberyl acetate	0.56	$[M+H]^{+1}$	terpenoid
264	C42 H62 O16	823.4088	38.954	Glycyrrhizin	-2.81	$[M+H]^{+1}$	terpenoid
265	C12 H16 O2	193.1224	39.5	Senkyunolide A	0.35	$[M+H]^{+1}$	phthalide
266	C10 H16 O	153.1274	40.971	DL-Camphor	0.15	$[M+H]^{+1}$	terpenoid
267	C8 H16 O	129.1277	41.128	Octanal	2.06	$[M+H]^{+1}$	others
268	C14 H20 O2	221.1537	41.371	Glutinosone	0.57	$[M+H]^{+1}$	others
269	C27 H42 O4	431.3157	41.536	Ruscogenin	0.27	$[M+H]^{+1}$	steroid
270	C11 H17 N O	180.1384	41.759	(+)-N-Methylephedrine	0.34	$[M+H]^{+1}$	alkaloid
271	C10 H12 O	149.0962	42.047	Aallylcresol	0.98	$[M+H]^{+1}$	terpenoid
272	C34 H28 O22	789.1119	42.938	Glucogallin	-3.25	$[M+H]^{+1}$	phenol
273	C57 H110 O6	891.8398	43.085	Tristearin	2.54	$[M+H]^{+1}$	others
274	C9 H6 O2	147.0441	43.2	Coumarin	0.08	$[M+H]^{+1}$	coumarin
275	C42 H64 O16	825.4253	43.326	Licoricesaponin J2	-1.71	$[M+H]^{+1}$	saponin
276	C9 H10 N2	147.0918	43.365	N-Ethylbenzimidazole	0.63	$[M+H]^{+1}$	others
277	C43 H48 O22	917.2664	43.697	Isorhamnetin 3-(4''-p-coumarylrobinobioside) -7-rhamnoside	-4.98	$[M+H]^{+1}$	flavonoid
278	C48 H72 O21	985.4673	43.719	LICORICESAPONIN A3	3.45	$[M+H]^{+1}$	saponin
279	C9 H7 N O2	162.055	43.913	3-Carboxyindole	0.04	$[M+H]^{+1}$	organic acid
280	C12 H12 O2	189.0911	44.163	9-butylidenephthalide	0.55	$[M+H]^{+1}$	others
281	C38 H60 O12	709.4174	44.486	Sprengerinin A	2.27	$[M+H]^{+1}$	steroid
282	C41 H64 O13	765.439	44.705	Ophiopogonin A	-3.85	$[M+H]^{+1}$	saponin
283	C18 H30 O2	279.2323	45.293	Alpha-Linolenic acid	1.48	$[M+H]^{+1}$	fatty acid
284	C14 H22 O	207.1744	45.458	4-Octylphenol	0.32	$[M+H]^{+1}$	phenol
285	C6 H9 N O3	144.0656	45.999	trimethadione	0.65	$[M+H]^{+1}$	others
286	C9 H16 O4	187.0968	46.566	Azelaic acid	-3.94	$[M-H]^{-1}$	organic acid

287	C28 H32 O19 S	705.1345	46.766	Isorhamnetin 3-(4"-sulfatorutinoside)	1.98	$[M+H]^{+1}$	flavonoid
288	C31 H62 O11	611.4388	46.964	Palmitone	3.86	$[M+H]^{+1}$	others
289	C12 H12 O3	205.0859	47.137	Senkyunolide B	0.08	$[M+H]^{+1}$	phthalide
290	C16 H22 O4	279.1591	47.148	Dibutyl phthalate	-0.02	$[M+H]^{+1}$	others
291	C51 H98 O6	807.7423	47.224	Tripalmitin	-1.7	$[M+H]^{+1}$	others
292	C6 H10 O2	115.0757	47.405	5-HEXENOIC ACID	2.74	$[M+H]^{+1}$	fatty acid
293	C42 H62 O15	807.4156	47.543	Licoricesaponin C2	-0.69	$[M+H]^{+1}$	saponin
294	C24 H28 O4	381.206	47.766	Angelicide	-0.19	$[M+H]^{+1}$	phthalide
295	C15 H24	205.1951	47.777	Cyperene	0.18	$[M+H]^{+1}$	terpenoid
296	C10 H12 O	149.0961	48.622	(E)-Anethole	0.07	$[M+H]^{+1}$	phenol
297	C7 H14 O7	209.0662	48.629	D-sedoheptulose	-2.08	$[M-H]^{-1}$	others
298	C27 H44 O	385.3471	49.064	Colecalciferol	1.68	$[M+H]^{+1}$	vitamin
299	C8 H16 O	129.1276	49.106	2-OCTEN^{-1}-OL	1.94	$[M+H]^{+1}$	fatty acid
300	C8 H14	111.117	49.156	3-ETHYL^{-1}，4-HEXADIENE	1.96	$[M+H]^{+1}$	others
301	C18 H36 O2	285.2788	49.525	Stearic acid	0.11	$[M+H]^{+1}$	fatty acid
302	C21 H32 O14	509.1886	50.212	6-O-β-glucosylaucubin	4.11	$[M+H]^{+1}$	flavonoid
303	C16 H32 O2	274.2733	50.45	Palmitic Acid	4.71	$[M+H]^{+1}$	fatty acid
304	C19 H38 O2	299.2964	50.457	Nonadecanoic acid	-0.28	$[M+H]^{+1}$	fatty acid
305	C12 H12 O3	205.0862	50.521	Senkyunolide E	1.5	$[M+H]^{+1}$	phthalide
306	C6 H8 O6	175.025	51.467	Ascorbic acid	1.33	$[M-H]^{-1}$	vitamin
307	C6 H6 N2 O	123.0554	52.09	Nicotinamide	1.19	$[M+H]^{+1}$	alkaloid
308	C8 H6 O4	167.0337	55.363	Phthalic acid	-1.26	$[M+H]^{+1}$	organic acid
309	C20 H38 O2	311.2939	55.369	5E-eicosenoic acid	-1.75	$[M+H]^{+1}$	fatty acid
310	C10 H16	137.1325	56.427	Crithmene	0.11	$[M+H]^{+1}$	terpenoid
311	C21 H42 O2	327.3272	56.566	Henicosanoic acid	4.26	$[M+H]^{+1}$	fatty acid
312	C7 H6 O4	153.0197	56.643	Protocatechuic acid	2.68	$[M-H]^{-1}$	phenol
313	C10 H13 N	148.112	56.795	4-methyl-5，6，7，8-tetrahydroquinoline	-0.62	$[M+H]^{+1}$	alkaloid
314	C30 H46 O4	471.3455	56.851	enoxolone	-3.01	$[M+H]^{+1}$	terpenoid
315	C16 H30 O2	255.2317	57.039	Palmitelaidic acid	-0.56	$[M+H]^{+1}$	fatty acid
316	C6 H7 N3 O	138.0661	57.266	Nicotinohydrazide	-0.44	$[M+H]^{+1}$	others
317	C27 H22 O18	635.0898	57.641	Strictinin	2.98	$[M+H]^{+1}$	phenol
318	C12 H14 O2	191.1065	58.144	(Z)-Ligustilide	-0.63	$[M+H]^{+1}$	others
319	C14 H22 O	207.1743	58.642	octylphenol	0	$[M+H]^{+1}$	terpenoid
320	C6 H6 O3	125.0234	59.154	Pyrogallol	-8.33	$[M-H]^{-1}$	phenol
321	C6 H5 N O2	124.0394	59.93	Niacin	0.88	$[M+H]^{+1}$	vitamin
