# Supplementary material for: Baihe Gujin decoction ameliorates sepsis-induced acute lung injury through Nrf2/GPX4-mediated antioxidant defense and PPARα-driven metabolic reprogramming: a multi-omics investigation
Source: Front Immunol. 2026 Jun 29;17:1767881. doi: 10.3389/fimmu.2026.1767881 (PMC13357210; doi:10.3389/fimmu.2026.1767881)
Supplement: Supplementary file 4 [file Table3.pdf]

**Table S3** The list of 508 differential metabolites(BHGJ-H VS model).

| Number | Metabolites | Super Class | Class | Sub Class | level | Formula | VIP | log2FoldChange | FoldChange | Regulation | p-value | q-value |
|---|---|---|---|---|---|---|---|---|---|---|---|---|
| 1 | 15-Dihydroxyeicosatrienoic acid | Lipids and lipid-like molecules | Fatty Acyls | Eicosanoids | Level 2 | C20H34O4 | 1.36243962 | -0.703118827 | 0.614242896 | Down | 1.45696E-05 | 0.014069648 |
| 2 | Prostaglandin A1 | Lipids and lipid-like molecules | Fatty Acyls | Eicosanoids | Level 2 | C20H32O4 | 2.183125316 | -1.819647632 | 0.283290155 | Down | 1.57577E-05 | 0.014069648 |
| 3 | trans-trans-Muconic acid | Lipids and lipid-like molecules | Fatty Acyls | Fatty acids and conjugates | Level 2 | C6H6O4 | 4.323251615 | 6.729728015 | 106.1328912 | Up | 1.77722E-05 | 0.014069648 |
| 4 | 5-F2t-IsoP | Lipids and lipid-like molecules | Fatty Acyls | Eicosanoids | Level 4 | C20H34O5 | 2.423878616 | -2.17903551 | 0.220823328 | Down | 3.08157E-05 | 0.014148524 |
| 5 | 10-hydroxy-8E-decenoic acid | Organic acids and derivatives | Hydroxy acids and derivatives | Medium-chain hydroxy acids and derivatives | Level 2 | C10H18O3 | 2.11673196 | -1.674288259 | 0.313320645 | Down | 3.52561E-05 | 0.014148524 |
| 6 | Cotinine | Organoheterocyclic compounds | Pyridines and derivatives | Pyrrolidinylpyridines | Level 4 | C10H12N2O | 1.892224027 | 1.391317414 | 2.623181103 | Up | 4.81374E-05 | 0.014148524 |
| 7 | S-Cysteinosuccinic acid | Organic acids and derivatives | Carboxylic acids and derivatives | Amino acids, peptides, and analogues | Level 2 | C7H11NO6S | 5.192539446 | -9.712871275 | 0.001191611 | Down | 4.94089E-05 | 0.014148524 |
| 8 | Quercetol A | Phenylpropanoids and polyketides | Flavonoids | Pyranoflavonoids | Level 2 | C21H22O5 | 1.795171965 | 1.242037297 | 2.365323149 | Up | 5.13243E-05 | 0.014148524 |
| 9 | Metenamine | Organoheterocyclic compounds | Triazinanes | 1,3,5-triazinanes | Level 2 | C6H12N4 | 5.137188598 | 10.19746059 | 1174.198487 | Up | 5.36155E-05 | 0.014148524 |
| 10 | Sweroside | Organic oxygen compounds | Organooxygen compounds | Carbohydrates and carbohydrate conjugates | Level 1 | C16H22O9 | 1.049710384 | 0.427597837 | 1.344992228 | Up | 6.372E-05 | 0.015133497 |
| 11 | triamterene | Organoheterocyclic compounds | Pteridines and derivatives | Unclassified | Level 4 | C12H11N7 | 1.160512719 | 0.522015017 | 1.435959458 | Up | 7.40192E-05 | 0.015981429 |
| 12 | 4-Vinylphenol sulfate | Organic acids and derivatives | Organic sulfuric acids and derivatives | Arylsulfates | Level 1 | C8H8O4S | 1.908083677 | 1.472590961 | 2.77519849 | Up | 0.000116026 | 0.022963549 |
| 13 | ent-11-epi-8-E2t-IsoP | Organic acids and derivatives | Hydroxy acids and derivatives | Medium-chain hydroxy acids and derivatives | Level 1 | C20H32O5 | 1.763657858 | -1.191232061 | 0.43792871 | Down | 0.000143937 | 0.025132506 |
| 14 | 2,2'-Methylenebis(4-methyl-6-tert-butylphenol) | Benzenoids | Benzene and substituted derivatives | Diphenylmethanes | Level 2 | C23H32O2 | 1.989136833 | 1.515108952 | 2.858204123 | Up | 0.00014815 | 0.025132506 |
| 15 | Butyrylcarnitine | Lipids and lipid-like molecules | Fatty Acyls | Fatty acid esters | Level 1 | C11H21NO4 | 1.459943175 | 0.874737498 | 1.833674414 | Up | 0.000176396 | 0.026212942 |
| 16 | 7-Methylnonanoylcarnitine | Lipids and lipid-like molecules | Fatty Acyls | Fatty acid esters | Level 3 | C17H33NO4 | 2.076277256 | 1.694424853 | 3.236478349 | Up | 0.000176592 | 0.026212942 |
| 17 | 3, 5-Tetradecadiencarnitine | Lipids and lipid-like molecules | Fatty Acyls | Fatty acid esters | Level 4 | C21H37NO4 | 1.852814069 | 1.380368253 | 2.60334814 | Up | 0.000228981 | 0.031989937 |
| 18 | Homoveratric acid | Benzenoids | Benzene and substituted derivatives | Methoxybenzenes | Level 2 | C10H12O4 | 1.05447532 | 0.454119031 | 1.369946004 | Up | 0.000258762 | 0.034142201 |
| 19 | 5,6beta-Dihydro PGI2 | Lipids and lipid-like molecules | Fatty Acyls | Eicosanoids | Level 1 | C20H34O5 | 1.814959458 | -1.287440564 | 0.409677179 | Down | 0.000328842 | 0.040990073 |
| 20 | Thiomorpholine 3-carboxylate | Organic acids and derivatives | Carboxylic acids and derivatives | Amino acids, peptides, and analogues | Level 2 | C5H9NO2S | 1.74518968 | -1.2121626 | 0.43162113 | Down | 0.000381385 | 0.040990073 |
| 21 | Adenosine monophosphate | Nucleosides, nucleotides, and analogues | Purine nucleotides | Purine ribonucleotides | Level 1 | C10H14N5O7P | 1.503547453 | 0.939114403 | 1.917350913 | Up | 0.000443863 | 0.040990073 |
| 22 | Sciadonic acid | Lipids and lipid-like molecules | Fatty Acyls | Fatty acids and conjugates | Level 2 | C20H34O2 | 1.309880979 | -0.678283518 | 0.624908332 | Down | 0.000481023 | 0.040990073 |
| 23 | 2-Hexenoylcarnitine | Lipids and lipid-like molecules | Fatty Acyls | Fatty acid esters | Level 1 | C13H23NO4 | 1.173518078 | 0.531802376 | 1.445734241 | Up | 0.000482016 | 0.040990073 |
| 24 | 5,7-Pentadecadiene | Hydrocarbons | Unsaturated hydrocarbons | Olefins | Level 4 | C15H28 | 1.832570078 | 1.377041227 | 2.597351427 | Up | 0.000485579 | 0.040990073 |
| 25 | (1S,2S,4R,8S)-p-Menthane-1,2,8,9-tetrol 2-glucoside | Lipids and lipid-like molecules | Prenol lipids | Terpene glycosides | Level 2 | C16H30O9 | 1.754790894 | -1.199668214 | 0.435375396 | Down | 0.000512164 | 0.040990073 |
| 26 | N-Palmitoyl Cysteine | Organic acids and derivatives | Carboxylic acids and derivatives | Amino acids, peptides, and analogues | Level 4 | C19H37NO3S | 1.88364516 | -1.394964505 | 0.380254044 | Down | 0.000537336 | 0.040990073 |
| 27 | Dodecanoylcarnitine | Lipids and lipid-like molecules | Fatty Acyls | Fatty acid esters | Level 1 | C19H37NO4 | 1.960106874 | 1.547374823 | 2.922848036 | Up | 0.000538804 | 0.040990073 |
| 28 | L-Octanoylcarnitine | Lipids and lipid-like molecules | Fatty Acyls | Fatty acid esters | Level 1 | C15H29NO4 | 2.014172196 | 1.650172276 | 3.138711171 | Up | 0.000542221 | 0.040990073 |
| 29 | 4-Hexadecenoylcarnitine | Lipids and lipid-like molecules | Fatty Acyls | Fatty acid esters | Level 2 | C23H43NO4 | 1.817067687 | 1.353991821 | 2.556184235 | Up | 0.000566477 | 0.040990073 |
| 30 | (4E,7E,13E)-Hexadeca-4,7,13-trienoylcarnitine | Lipids and lipid-like molecules | Fatty Acyls | Fatty acid esters | Level 4 | C23H39NO4 | 2.065549596 | 1.753655536 | 3.372119203 | Up | 0.000570196 | 0.040990073 |
| 31 | Xanthine | Organoheterocyclic compounds | Imidazopyrimidines | Purines and purine derivatives | Level 1 | C5H4N4O2 | 1.254837908 | -0.620811325 | 0.650305114 | Down | 0.000588999 | 0.040990073 |
| 32 | 3-hydroxyhexadecanoyl carnitine | Lipids and lipid-like molecules | Fatty Acyls | Fatty acid esters | Level 2 | C23H45NO5 | 1.677304801 | 1.170001479 | 2.250119276 | Up | 0.000619278 | 0.040990073 |
| 33 | 3-(3,4-dimethoxyphenyl)-2-methylpropanoic acid | Phenylpropanoids and polyketides | Phenylpropanoic acids | Unclassified | Level 4 | C12H16O4 | 1.585180133 | -1.040722261 | 0.486084063 | Down | 0.000620954 | 0.040990073 |
| 34 | 2-Ethyl-2-heptenal | Organic oxygen compounds | Organooxygen compounds | Carbonyl compounds | Level 4 | C9H16O | 1.578107315 | 1.034178636 | 2.047947364 | Up | 0.000651266 | 0.040990073 |
| 35 | cis-8,11,14-Eicosatrienoic Acid | Lipids and lipid-like molecules | Fatty Acyls | Fatty acids and conjugates | Level 1 | C20H34O2 | 1.280952673 | -0.655126028 | 0.635020016 | Down | 0.000652701 | 0.040990073 |
| 36 | 5-Methoxy-2-methylthiazole | Organoheterocyclic compounds | Azoles | Thiazoles | Level 4 | C5H7NOS | 4.357509026 | -7.280495074 | 0.006432097 | Down | 0.000671368 | 0.040990073 |
| 37 | Tetradecenoylcarnitine | Lipids and lipid-like molecules | Fatty Acyls | Fatty acids and conjugates | Level 1 | C21H39NO4 | 2.213076296 | 1.974498478 | 3.929916006 | Up | 0.000679787 | 0.040990073 |
| 38 | 12-Hydroxydodecanoylcarnitine | Lipids and lipid-like molecules | Fatty Acyls | Fatty acid esters | Level 2 | C19H37NO5 | 1.91230819 | 1.513816455 | 2.85564463 | Up | 0.000706332 | 0.040990073 |
| 39 | 6-(4-ethenylphenoxy)-3,4,5-trihydroxyoxane-2-carboxylic acid | Organic oxygen compounds | Organooxygen compounds | Carbohydrates and carbohydrate conjugates | Level 3 | C14H16O7 | 2.026964392 | 1.648433367 | 3.134930298 | Up | 0.000716505 | 0.040990073 |
| 40 | (-)-11-hydroxy-9,10-dihydrojasmonic acid | Lipids and lipid-like molecules | Fatty Acyls | Lineolic acids and derivatives | Level 2 | C12H20O4 | 1.443482457 | 0.89315805 | 1.857237158 | Up | 0.000717994 | 0.040990073 |
| 41 | Bilobalide A | Lipids and lipid-like molecules | Prenol lipids | Terpene lactones | Level 4 | C15H18O8 | 2.169119674 | -1.928245855 | 0.262748448 | Down | 0.000738852 | 0.040990073 |
| 42 | L-Histidine | Organic acids and derivatives | Carboxylic acids and derivatives | Amino acids, peptides, and analogues | Level 1 | C6H9N3O2 | 1.213726891 | -0.566475868 | 0.675264272 | Down | 0.00074493 | 0.040990073 |
| 43 | MG(16:0/0:0/0:0)[rac] | Lipids and lipid-like molecules | Glycerolipids | Monoradylglycerols | Level 2 | C19H38O4 | 2.304975137 | -2.162213089 | 0.22341329 | Down | 0.000776842 | 0.040990073 |
| 44 | Dansylglutamyl-glycyl-arginine chloromethyl ketone | Organic acids and derivatives | Carboxylic acids and derivatives | Amino acids, peptides, and analogues | Level 4 | C26H36ClN7O7S | 1.312276359 | -0.688739824 | 0.620395521 | Down | 0.000779759 | 0.040990073 |
| 45 | PA(17:2/19:0) | Lipids and lipid-like molecules | Glycerophospholipids | Glycerophosphates | Level 3 | C39H73O8P | 2.953172402 | -3.458078535 | 0.090994393 | Down | 0.00078913 | 0.040990073 |
| 46 | 2-(2'-trans-pentenyl-5'-hydroxy)-3-methyl-2-cyclopenten-1-one | Lipids and lipid-like molecules | Fatty Acyls | Fatty alcohols | Level 2 | C11H16O2 | 1.641725407 | -1.12881505 | 0.457291164 | Down | 0.000861476 | 0.040990073 |
| 47 | L-Acetylcarnitine | Lipids and lipid-like molecules | Fatty Acyls | Fatty acid esters | Level 1 | C9H18NO4+ | 1.253990864 | 0.667579717 | 1.588406002 | Up | 0.000861566 | 0.040990073 |
| 48 | (10Z)-12-Hydroxyhexadecenoylcarnitine | Lipids and lipid-like molecules | Fatty Acyls | Fatty acid esters | Level 1 | C23H43NO5 | 2.098938158 | 1.829428515 | 3.553962639 | Up | 0.000865353 | 0.040990073 |
| 49 | Brunfelsamidine | Organoheterocyclic compounds | Pyrroles | Substituted pyrroles | Level 1 | C5H7N3 | 1.225066465 | -0.602639997 | 0.658547771 | Down | 0.0008774 | 0.040990073 |
| 50 | 2-(2-Hydroxypropan-2-ylamino)propan-2-ol | Organic nitrogen compounds | Organonitrogen compounds | Amines | Level 2 | C6H15NO2 | 1.854103076 | -1.463800599 | 0.362536812 | Down | 0.000883461 | 0.040990073 |
| 51 | Isobutyryl-L-carnitine | Lipids and lipid-like molecules | Fatty Acyls | Fatty acid esters | Level 1 | C11H22NO4+ | 1.51186148 | 0.988512385 | 1.984138019 | Up | 0.000888491 | 0.040990073 |
| 52 | gamma-Glutamylasparagine | Organic acids and derivatives | Carboxylic acids and derivatives | Amino acids, peptides, and analogues | Level 2 | C9H15N3O6 | 1.376649361 | -0.758592532 | 0.59107269 | Down | 0.000897467 | 0.040990073 |
| 53 | Acetoacetic acid | Organic acids and derivatives | Keto acids and derivatives | Short-chain keto acids and derivatives | Level 2 | C4H6O3 | 1.171331597 | 0.571370818 | 1.485934803 | Up | 0.000961844 | 0.042776517 |
| 54 | LysoPC(O-14:0/0:0) | Lipids and lipid-like molecules | Glycerophospholipids | Glycerophosphocholines | Level 2 | C22H48NO6P | 1.554158312 | 1.033323812 | 2.046734276 | Up | 0.000972603 | 0.042776517 |
| 55 | Adenylosuccinic acid | Nucleosides, nucleotides, and analogues | Purine nucleotides | Purine ribonucleotides | Level 1 | C14H18N5O11P | 1.58575111 | 1.097332915 | 2.139587848 | Up | 0.001016068 | 0.043875671 |
| 56 | omega-3-Arachidonic acid | Lipids and lipid-like molecules | Fatty Acyls | Fatty acids and conjugates | Level 2 | C20H32O2 | 1.501032072 | -0.916843183 | 0.529666738 | Down | 0.001046473 | 0.044381653 |
| 57 | 3-Butynoic acid | Lipids and lipid-like molecules | Fatty Acyls | Fatty acids and conjugates | Level 2 | C4H4O2 | 1.129624647 | 0.553839957 | 1.467987778 | Up | 0.001137319 | 0.047388282 |
| 58 | D-erythro-D-galacto-octitol | Organic oxygen compounds | Organooxygen compounds | Carbohydrates and carbohydrate conjugates | Level 4 | C8H18O8 | 1.32112185 | -0.737354622 | 0.599838229 | Down | 0.001160118 | 0.047504834 |
| 59 | Sepil | Phenylpropanoids and polyketides | Flavonoids | O-methylated flavonoids | Level 4 | C16H14O7 | 2.342412872 | 2.265506428 | 4.808231732 | Up | 0.001182774 | 0.047611663 |
| 60 | 13-Methylhenicosanoylcarnitine | Lipids and lipid-like molecules | Fatty Acyls | Fatty acid esters | Level 4 | C29H57NO4 | 1.394394722 | -0.769788259 | 0.586503548 | Down | 0.001244843 | 0.048501949 |
| 61 | N-Oleoyl Cysteine | Organic acids and derivatives | Carboxylic acids and derivatives | Amino acids, peptides, and analogues | Level 2 | C21H39NO3S | 1.61437333 | -1.067887359 | 0.477017018 | Down | 0.001248045 | 0.048501949 |
| 62 | Gly-Phe-Glu | Organic acids and derivatives | Carboxylic acids and derivatives | Amino acids, peptides, and analogues | Level 2 | C16H21N3O6 | 1.60847477 | -1.059872245 | 0.479674534 | Down | 0.001280964 | 0.048501949 |
| 63 | Oleoylcarnitine | Lipids and lipid-like molecules | Fatty Acyls | Fatty acid esters | Level 1 | C25H48NO4+ | 1.430145997 | 0.885084824 | 1.846873207 | Up | 0.001286578 | 0.048501949 |

| | | | | | | | | | | | | | | |
|---|---|---|---|---|---|---|---|---|---|---|---|---|---|---|
| 64 | Cysteineglutathione disulfide | Organic acids and derivatives | Carboxylic acids and derivatives | Amino acids, peptides, and analogues | Level 1 | C13H22N4O8S2 | 2.803683777 | -3.30834814 | 0.100945735 | Down | 0.00136991 | 0.049041603 |
| 65 | 2'-Deoxyguanosine 5'-monophosphate | Nucleosides, nucleotides, and analogues | Purine nucleotides | Purine deoxyribonucleotides | Level 2 | C10H14N5O7P | 1.391559615 | 0.808740729 | 1.751681802 | Up | 0.00140844 | 0.049041603 |
| 66 | 2-Hydroxyhexan-3-one | Organic oxygen compounds | Organooxygen compounds | Carbohydrates and carbohydrate conjugates | Level 4 | C6H12O2 | 1.67264185 | 1.193156453 | 2.28652462 | Up | 0.001431078 | 0.049041603 |
| 67 | 2-O-(6-Phospho-alpha-mannosyl)-D-glycerate | Organic oxygen compounds | Organooxygen compounds | Carbohydrates and carbohydrate conjugates | Level 2 | C9H17O12P | 1.408649182 | -0.866275989 | 0.548561019 | Down | 0.001436357 | 0.049041603 |
| 68 | Zwiebelane B | Organoheterocyclic compounds | Thiolanes | Unclassified | Level 4 | C6H10OS2 | 2.11762051 | 1.813344929 | 3.514562058 | Up | 0.001439436 | 0.049041603 |
| 69 | Crotonyl isothiocyanate | Organosulfur compounds | Isothiocyanates | Isothiocyanate acids | Level 4 | C5H5NOS | 3.449931459 | -4.790520259 | 0.036133473 | Down | 0.001442697 | 0.049041603 |
| 70 | Tetradecanoylcarnitine | Lipids and lipid-like molecules | Fatty Acyls | Fatty acid esters | Level 1 | C21H41NO4 | 1.554600307 | 1.013023358 | 2.018135942 | Up | 0.001476304 | 0.049041603 |
| 71 | 4-Methylmannitol | Organic oxygen compounds | Organooxygen compounds | Carbohydrates and carbohydrate conjugates | Level 4 | C7H16O6 | 1.461480476 | -0.880288363 | 0.543258835 | Down | 0.001487327 | 0.049041603 |
| 72 | 2-Quinolinylmethanol | Organoheterocyclic compounds | Quinolines and derivatives | Unclassified | Level 4 | C10H9NO | 1.778740463 | 1.322955926 | 2.50178173 | Up | 0.001504045 | 0.049041603 |
| 73 | Arachidonic acid | Lipids and lipid-like molecules | Fatty Acyls | Fatty acids and conjugates | Level 1 | C20H32O2 | 1.208958723 | -0.609490812 | 0.65542799 | Down | 0.001508837 | 0.049041603 |
| 74 | Thromboxane A2 | Lipids and lipid-like molecules | Fatty Acyls | Eicosanoids | Level 2 | C20H32O5 | 1.795716193 | 1.347632824 | 2.544942082 | Up | 0.001532017 | 0.049041603 |
| 75 | 4-(Aminomethyl)-1-methylpiperidin-4-ol | Organoheterocyclic compounds | Piperidines | Unclassified | Level 2 | C7H16N2O | 2.465018237 | 2.487016476 | 5.606173821 | Up | 0.001678641 | 0.052457528 |
| 76 | trans-2-Dodecenoylcarnitine | Lipids and lipid-like molecules | Fatty Acyls | Fatty acid esters | Level 1 | C19H35NO4 | 1.535014608 | 1.057671504 | 2.081569176 | Up | 0.001707497 | 0.052666316 |
| 77 | Glu-Thr-His | Organic acids and derivatives | Carboxylic acids and derivatives | Amino acids, peptides, and analogues | Level 2 | C15H23N5O7 | 1.985046638 | -1.608239174 | 0.327998433 | Down | 0.00182878 | 0.055684011 |
| 78 | 2,15-dihydroxy-pentadecylic acid | Lipids and lipid-like molecules | Fatty Acyls | Fatty acids and conjugates | Level 4 | C15H30O4 | 1.541943613 | 1.037428982 | 2.052566526 | Up | 0.001891853 | 0.056177938 |
| 79 | 11-oxo-undeca-5,9-dienoic acid | Lipids and lipid-like molecules | Fatty Acyls | Fatty acids and conjugates | Level 3 | C11H16O3 | 1.081011079 | 0.529952354 | 1.44388151 | Up | 0.001923408 | 0.056177938 |
| 80 | (2E,4Z)-decadienoylcarnitine | Lipids and lipid-like molecules | Fatty Acyls | Fatty acid esters | Level 2 | C17H29NO4 | 1.695318151 | 1.254288417 | 2.385494595 | Up | 0.001926531 | 0.056177938 |
| 81 | L-Rhamnulose | Organic oxygen compounds | Organooxygen compounds | Carbohydrates and carbohydrate conjugates | Level 2 | C6H12O5 | 1.324622565 | -0.782710783 | 0.58127357 | Down | 0.00195278 | 0.056177938 |
| 82 | Proline betaine | Organic acids and derivatives | Carboxylic acids and derivatives | Amino acids, peptides, and analogues | Level 1 | C7H13NO2 | 1.480756532 | 0.978237435 | 1.970057086 | Up | 0.001975588 | 0.056177938 |
| 83 | Stearic acid | Lipids and lipid-like molecules | Fatty Acyls | Fatty acids and conjugates | Level 1 | C18H36O2 | 0.853545336 | -0.311157159 | 0.805995026 | Down | 0.001996408 | 0.056177938 |
| 84 | 3-methyl-octadecanoic acid | Lipids and lipid-like molecules | Fatty Acyls | Fatty acids and conjugates | Level 4 | C19H38O2 | 1.256298141 | -0.682150714 | 0.623235486 | Down | 0.002019745 | 0.056177938 |
| 85 | 3-Sulfinoalanine | Organic acids and derivatives | Carboxylic acids and derivatives | Amino acids, peptides, and analogues | Level 1 | C3H7NO4S | 2.227534864 | 2.111378836 | 4.321040747 | Up | 0.002043152 | 0.056177938 |
| 86 | Glutathione | Organic acids and derivatives | Carboxylic acids and derivatives | Amino acids, peptides, and analogues | Level 1 | C10H17N3O6S | 1.546452238 | 0.975893256 | 1.966858617 | Up | 0.002057887 | 0.056177938 |
| 87 | Leptinidine | Lipids and lipid-like molecules | Steroids and steroid derivatives | Steroidal alkaloids | Level 2 | C27H43NO2 | 1.987190778 | 1.799864617 | 3.481875498 | Up | 0.002088083 | 0.056354517 |
| 88 | (12E)-10-Hydroxytetradec-12-enoylcarnitine | Lipids and lipid-like molecules | Fatty Acyls | Fatty acid esters | Level 2 | C21H39NO5 | 2.010351163 | 1.7387541 | 3.337468221 | Up | 0.002137828 | 0.057048783 |
| 89 | Muramic acid | Organic oxygen compounds | Organooxygen compounds | Carbohydrates and carbohydrate conjugates | Level 2 | C9H17NO7 | 1.467164181 | -0.913283521 | 0.530975235 | Down | 0.002170295 | 0.057271664 |
| 90 | MG(18:0/0:0) | Lipids and lipid-like molecules | Glycerolipids | Monoradylglycerols | Level 2 | C21H42O4 | 1.359356458 | -0.771171605 | 0.585941442 | Down | 0.002228841 | 0.058170304 |
| 91 | 5S-Hp-18R-HEPE | Lipids and lipid-like molecules | Fatty Acyls | Eicosanoids | Level 2 | C20H30O5 | 3.387129118 | 4.739179652 | 26.70762257 | Up | 0.002302824 | 0.059447913 |
| 92 | hydroxytyrosyl acetate | Organic acids and derivatives | Carboxylic acids and derivatives | Amino acids, peptides, and analogues | Level 2 | C11H13NO5 | 2.261169364 | -2.040817474 | 0.243025992 | Down | 0.002330344 | 0.05951147 |
| 93 | 16-phenyl-tetranor-PGE2 | Lipids and lipid-like molecules | Fatty Acyls | Eicosanoids | Level 1 | C22H28O5 | 1.8010545 | -1.383335536 | 0.383331501 | Down | 0.002358303 | 0.059584774 |
| 94 | Palmitic acid | Lipids and lipid-like molecules | Fatty Acyls | Fatty acids and conjugates | Level 1 | C16H32O2 | 0.930765167 | -0.389822931 | 0.763223273 | Down | 0.002431121 | 0.059845013 |
| 95 | 2,5-Dihydro-1H-pyrrole-2-carboxylic acid | Organic acids and derivatives | Carboxylic acids and derivatives | Amino acids, peptides, and analogues | Level 2 | C5H7NO2 | 3.848536284 | 5.839503983 | 57.26191377 | Up | 0.002446665 | 0.059845013 |
| 96 | 6Z,9Z-Heptadecadiene | Hydrocarbons | Unsaturated hydrocarbons | Olefins | Level 4 | C17H32 | 1.483865429 | 0.977315682 | 1.968798798 | Up | 0.002457697 | 0.059845013 |
| 97 | (5,7)-Decadienoylcarnitine | Lipids and lipid-like molecules | Fatty Acyls | Fatty acid esters | Level 2 | C17H29NO4 | 1.723862636 | 1.264909837 | 2.403121909 | Up | 0.002469394 | 0.059845013 |
| 98 | 8-iso-15-keto-PGE2 | Lipids and lipid-like molecules | Fatty Acyls | Eicosanoids | Level 1 | C20H30O5 | 1.754157161 | -1.322235216 | 0.399914857 | Down | 0.002566701 | 0.0615749 |
| 99 | LysoPE(0:0/18:0) | Lipids and lipid-like molecules | Glycerophospholipids | Glycerophosphoethanolamines | Level 1 | C23H48NO7P | 1.1560746 | -0.584878202 | 0.666705622 | Down | 0.002605018 | 0.061869177 |
| 100 | 3,9,15-Docosatriynoic acid | Lipids and lipid-like molecules | Fatty Acyls | Fatty acids and conjugates | Level 4 | C22H32O2 | 0.935794873 | -0.373025086 | 0.772161707 | Down | 0.002676128 | 0.062928756 |
| 101 | Cicaprost | Lipids and lipid-like molecules | Prenol lipids | Monoterpenoids | Level 2 | C22H30O5 | 1.354513154 | -0.77749625 | 0.583378349 | Down | 0.002716735 | 0.063257301 |
| 102 | PA(P-18:0/14:1) | Lipids and lipid-like molecules | Glycerophospholipids | Glycerophosphates | Level 4 | C35H67O7P | 1.179376672 | -0.597142826 | 0.661061854 | Down | 0.002820695 | 0.063924109 |
| 103 | Zooxanthellactone | Organoheterocyclic compounds | Lactones | Gamma butyrolactones | Level 2 | C22H30O2 | 0.977913513 | -0.416194832 | 0.74939859 | Down | 0.002883585 | 0.063924109 |
| 104 | Firibastat | Organic acids and derivatives | Organic sulfonic acids and derivatives | Organosulfonic acids and derivatives | Level 4 | C8H20N2O6S4 | 1.181571786 | 0.652110502 | 1.571465394 | Up | 0.002892427 | 0.063924109 |
| 105 | Trimethylethylammonium | Organic nitrogen compounds | Organonitrogen compounds | Quaternary ammonium salts | Level 4 | C5H14N+ | 3.283484802 | 4.432160036 | 21.58803514 | Up | 0.002905972 | 0.063924109 |
| 106 | Ustusoic acid A | Lipids and lipid-like molecules | Fatty Acyls | Fatty acid esters | Level 2 | C23H32O6 | 2.900318623 | -3.468470821 | 0.090341281 | Down | 0.00291973 | 0.063924109 |
| 107 | Hexanoylcarnitine | Lipids and lipid-like molecules | Fatty Acyls | Fatty acid esters | Level 1 | C13H26NO4+ | 1.790948699 | 1.363159671 | 2.572479661 | Up | 0.002935766 | 0.063924109 |
| 108 | (2E)-Pentadec-2-enoylcarnitine | Lipids and lipid-like molecules | Fatty Acyls | Fatty acid esters | Level 4 | C22H41NO4 | 1.3616784 | 0.812965388 | 1.756818788 | Up | 0.002955357 | 0.063924109 |
| 109 | Cer(d16:2/18:0) | Lipids and lipid-like molecules | Sphingolipids | Ceramides | Level 3 | C34H65NO3 | 1.405037491 | -0.844805728 | 0.55678578 | Down | 0.002964789 | 0.063924109 |
| 110 | Pentadecanoylcarnitine | Lipids and lipid-like molecules | Fatty Acyls | Fatty acid esters | Level 2 | C22H43NO4 | 1.146738738 | -0.567504451 | 0.674783008 | Down | 0.002987611 | 0.063924109 |
| 111 | N-Acetyllactosamine | Organic oxygen compounds | Organooxygen compounds | Carbohydrates and carbohydrate conjugates | Level 2 | C14H25NO11 | 1.548457271 | -1.109500853 | 0.46345435 | Down | 0.003071585 | 0.065134052 |
| 112 | Actinidic acid | Lipids and lipid-like molecules | Prenol lipids | Triterpenoids | Level 2 | C30H46O5 | 2.19471549 | 2.128812239 | 4.373572589 | Up | 0.003240919 | 0.068116651 |
| 113 | Tyrosylglycine | Organic acids and derivatives | Carboxylic acids and derivatives | Amino acids, peptides, and analogues | Level 2 | C11H14N2O4 | 1.695763144 | -1.230650341 | 0.426125313 | Down | 0.003397444 | 0.070780077 |
| 114 | 5-Hydroxydec-8-enoylcarnitine | Lipids and lipid-like molecules | Fatty Acyls | Fatty acid esters | Level 1 | C17H31NO5 | 1.211904322 | 0.661378174 | 1.581592763 | Up | 0.003434195 | 0.070923595 |
| 115 | hexadecanedioic acid mono-L-carnitine ester | Lipids and lipid-like molecules | Fatty Acyls | Fatty acid esters | Level 4 | C23H43NO6 | 1.423038135 | 0.900285626 | 1.866435465 | Up | 0.003502268 | 0.0712561 |
| 116 | Sphingofungin E | Lipids and lipid-like molecules | Fatty Acyls | Fatty acids and conjugates | Level 3 | C21H39NO7 | 4.446625391 | 8.247024358 | 303.8097495 | Up | 0.0035103 | 0.0712561 |
| 117 | Pro-Leu-Gln | Organic acids and derivatives | Carboxylic acids and derivatives | Amino acids, peptides, and analogues | Level 2 | C16H28N4O5 | 1.309409857 | 0.7403052 | 1.670529199 | Up | 0.003549408 | 0.071439356 |
| 118 | Alpha-linolenyl carnitine | Lipids and lipid-like molecules | Fatty Acyls | Fatty acid esters | Level 2 | C25H43NO4 | 1.203646096 | 0.665993895 | 1.586660972 | Up | 0.003627985 | 0.072210966 |
| 119 | 2-Hydroxyestradiol 17-sulfate | Lipids and lipid-like molecules | Steroids and steroid derivatives | Sulfated steroids | Level 4 | C18H24O6S | 4.160109781 | 7.171534979 | 144.1607867 | Up | 0.003665004 | 0.072210966 |
| 120 | 5-Hydroxytryptoline | Organoheterocyclic compounds | Indoles and derivatives | Pyridoindoles | Level 4 | C11H12N2O | 4.323831995 | 7.774621448 | 218.9748618 | Up | 0.003679163 | 0.072210966 |
| 121 | Acetaminophen mercapturate | Organic acids and derivatives | Carboxylic acids and derivatives | Amino acids, peptides, and analogues | Level 2 | C13H16N2O5S | 3.419812862 | -4.749985616 | 0.037163093 | Down | 0.003730267 | 0.072210966 |
| 122 | Dihydrobiopterin | Organoheterocyclic compounds | Pteridines and derivatives | Pterins and derivatives | Level 2 | C9H13N5O3 | 1.894922328 | -1.547656444 | 0.342065274 | Down | 0.003739768 | 0.072210966 |
| 123 | 10-Hydroxydodecanoylcarnitine | Lipids and lipid-like molecules | Fatty Acyls | Fatty acid esters | Level 4 | C19H37NO5 | 1.653881091 | 1.18355532 | 2.271358324 | Up | 0.003857943 | 0.073892055 |
| 124 | Tyrosyl-Glutamate | Organic acids and derivatives | Carboxylic acids and derivatives | Amino acids, peptides, and analogues | Level 4 | C14H18N2O6 | 1.595075468 | -1.067750564 | 0.47706225 | Down | 0.003924511 | 0.074565713 |
| 125 | Lidoflazine | Benzenoids | Benzene and substituted derivatives | Diphenylmethanes | Level 3 | C30H35F2N3O | 4.691077583 | 9.174562808 | 577.8546365 | Up | 0.003963984 | 0.074717944 |
| 126 | 8-oxo-Resolvin D1 | Lipids and lipid-like molecules | Fatty Acyls | Fatty acids and conjugates | Level 2 | C22H30O5 | 2.014458194 | 1.764623387 | 3.397852878 | Up | 0.004009151 | 0.074974289 |
| 127 | Dinorchenodeoxycholic acid | Lipids and lipid-like molecules | Steroids and steroid derivatives | Bile acids, alcohols and derivatives | Level 2 | C22H36O4 | 1.597382429 | 1.113188224 | 2.163231739 | Up | 0.004059944 | 0.075267457 |
| 128 | PKHdiA-PE | Lipids and lipid-like molecules | Glycerophospholipids | Glycerophosphoethanolamines | Level 4 | C28H50NO11P | 1.249989884 | 0.714821518 | 1.641280155 | Up | 0.004113919 | 0.075267457 |
| 129 | Lignoceroylcarnitine | Lipids and lipid-like molecules | Fatty Acyls | Fatty acid esters | Level 4 | C31H62NO4+ | 1.062027721 | -0.50415539 | 0.705073034 | Down | 0.004142706 | 0.075267457 |
| 130 | 3,4-Dehydrocilostazol | Organoheterocyclic compounds | Quinolines and derivatives | Quinolones and derivatives | Level 4 | C20H25N5O2 | 2.301587093 | -2.284077636 | 0.205316626 | Down | 0.004203266 | 0.075267457 |
| 131 | 14,15-LTC4 | Organic acids and derivatives | Carboxylic acids and derivatives | Amino acids, peptides, and analogues | Level 1 | C30H47N3O9S | 1.683137445 | -1.23699285 | 0.424256053 | Down | 0.004211903 | 0.075267457 |
| 132 | 11,12,15-TriHETRE | Lipids and lipid-like molecules | Fatty Acyls | Eicosanoids | Level 2 | C20H34O5 | 2.276915145 | 2.245987372 | 4.743616477 | Up | 0.004214978 | 0.075267457 |

| | | | | | | | | | | | | | |
|---|---|---|---|---|---|---|---|---|---|---|---|---|---|
| 133 | L-Arginine | Organic acids and derivatives | Carboxylic acids and derivatives | Amino acids, peptides, and analogues | Level 1 | C6H14N4O2 | 1.31926614 | -0.718535961 | 0.607713834 | Down | 0.004362864 | 0.077326883 |
| 134 | 2,4,7,9-Tetramethyl-5-decyne-4,7-diol | Organic oxygen compounds | Organooxygen compounds | Carbonyl compounds | Level 4 | C14H26O2 | 2.427226959 | 2.536951755 | 5.803614749 | Up | 0.004488236 | 0.078109185 |
| 135 | 16-Methylheptadecyl isobutyrate | Lipids and lipid-like molecules | Fatty Acyls | Fatty alcohol esters | Level 4 | C22H44O2 | 1.235001887 | -0.705648306 | 0.613166887 | Down | 0.004519518 | 0.078109185 |
| 136 | LysoPE(P-16:0/0:0) | Lipids and lipid-like molecules | Glycerophospholipids | Glycerophosphoethanolamines | Level 1 | C21H44NO6P | 0.891773436 | -0.351691519 | 0.783664734 | Down | 0.004526259 | 0.078109185 |
| 137 | NS00117762 | Organoheterocyclic compounds | Indoles and derivatives | Pyrroloindoles | Level 3 | C12H12N2O2 | 4.8170971 | 9.793628438 | 887.5154587 | Up | 0.004538555 | 0.078109185 |
| 138 | Lipoxin B4 | Lipids and lipid-like molecules | Fatty Acyls | Eicosanoids | Level 1 | C20H32O5 | 1.443158808 | -0.904236508 | 0.534315395 | Down | 0.004630089 | 0.078878537 |
| 139 | (1-Hydroxy-3-oxopropan-2-yl) dihydrogen phosphate | Organic acids and derivatives | Organic phosphoric acids and derivatives | Phosphate esters | Level 1 | C3H7O6P | 1.180547188 | -0.624620246 | 0.64859048 | Down | 0.004649682 | 0.078878537 |
| 140 | Serylvaline | Organic acids and derivatives | Carboxylic acids and derivatives | Amino acids, peptides, and analogues | Level 2 | C8H16N2O4 | 1.129123655 | 0.585585366 | 1.500647746 | Up | 0.004764728 | 0.079343549 |
| 141 | 3-Amino-5-(3-thiophenyl)-2-thiophenecarboxamide | Organoheterocyclic compounds | Bi- and oligothiophenes | Unclassified | Level 3 | C9H8N2OS2 | 1.15888904 | -0.611101357 | 0.654696714 | Down | 0.004802581 | 0.079343549 |
| 142 | PC(18:1/2:0) | Lipids and lipid-like molecules | Glycerophospholipids | Glycerophosphocholines | Level 4 | C28H54NO8P | 1.61802022 | -1.138796559 | 0.454138244 | Down | 0.004803834 | 0.079343549 |
| 143 | Cholestane-3b,5a,6b,(25R)26-tetrol | Lipids and lipid-like molecules | Steroids and steroid derivatives | Bile acids, alcohols and derivatives | Level 2 | C27H48O4 | 1.036636459 | 0.512968813 | 1.426983658 | Up | 0.004843947 | 0.079343549 |
| 144 | Maltol | Organoheterocyclic compounds | Pyrans | Pyranones and derivatives | Level 2 | C6H6O3 | 1.213514739 | -0.645032556 | 0.63947836 | Down | 0.004844132 | 0.079343549 |
| 145 | Idalopirdine | Organoheterocyclic compounds | Indoles and derivatives | Tryptamines and derivatives | Level 4 | C20H19F5N2O | 3.689240307 | 5.882038637 | 58.97528731 | Up | 0.004964285 | 0.079901778 |
| 146 | Mannosamine | Organic oxygen compounds | Organooxygen compounds | Carbohydrates and carbohydrate conjugates | Level 2 | C6H13NO5 | 4.44090031 | 8.407548181 | 339.5659951 | Up | 0.004976074 | 0.079901778 |
| 147 | Voglibose | Organic oxygen compounds | Organooxygen compounds | Alcohols and polyols | Level 2 | C10H21NO7 | 1.353244407 | -0.826126683 | 0.564041538 | Down | 0.004986543 | 0.079901778 |
| 148 | AKOS009363372 | Organic acids and derivatives | Carboxylic acids and derivatives | Amino acids, peptides, and analogues | Level 4 | C11H20N2O4 | 1.241350468 | -0.683705591 | 0.622564151 | Down | 0.005012785 | 0.079901778 |
| 149 | 3-Phosphoglyceric acid | Organic oxygen compounds | Organooxygen compounds | Carbohydrates and carbohydrate conjugates | Level 1 | C3H7O7P | 1.048601728 | -0.50766231 | 0.703361216 | Down | 0.005191218 | 0.081746048 |
| 150 | 4-Thiazolidinone | Organoheterocyclic compounds | Azolidines | Thiazolidines | Level 4 | C3H5NOS | 2.504230439 | 2.666082147 | 6.347032137 | Up | 0.005205391 | 0.081746048 |
| 151 | Spermic acid 1 | Organic acids and derivatives | Carboxylic acids and derivatives | Amino acids, peptides, and analogues | Level 1 | C10H23N3O2 | 1.532585298 | 1.083532056 | 2.119218079 | Up | 0.005231747 | 0.081746048 |
| 152 | Gln-Phe-Thr | Organic acids and derivatives | Carboxylic acids and derivatives | Amino acids, peptides, and analogues | Level 2 | C18H26N4O6 | 1.649755954 | -1.209951186 | 0.432283242 | Down | 0.005316328 | 0.082272359 |
| 153 | 15-(3-Methyl-5-pentylfuran-2-yl)pentadecanoylcarnitine | Lipids and lipid-like molecules | Fatty Acyls | Fatty acid esters | Level 1 | C32H57NO5 | 0.988216397 | -0.43373156 | 0.740344391 | Down | 0.005334713 | 0.082272359 |
| 154 | 4-epi-11-D4c-NeuroP | Lipids and lipid-like molecules | Fatty Acyls | Fatty alcohols | Level 4 | C22H32O5 | 2.647800286 | -3.369681973 | 0.096744136 | Down | 0.005387884 | 0.082556283 |
| 155 | SCHEMBL5032124 | Organic acids and derivatives | Carboxylic acids and derivatives | Amino acids, peptides, and analogues | Level 3 | C10H18N2O3 | 2.337336836 | -2.532167933 | 0.172878704 | Down | 0.005423293 | 0.082566164 |
| 156 | SCHEMBL21550299 | Organic acids and derivatives | Carboxylic acids and derivatives | Amino acids, peptides, and analogues | Level 4 | C13H25NO4 | 1.032205226 | 0.520016232 | 1.433971382 | Up | 0.005603862 | 0.084771798 |
| 157 | 4-Trimethylammoniobutanoic acid | Lipids and lipid-like molecules | Fatty Acyls | Fatty acids and conjugates | Level 2 | C7H15NO2 | 0.859749958 | 0.353531641 | 1.277684506 | Up | 0.005641436 | 0.084800065 |
| 158 | 13S,14S-epoxy-Maresin | Lipids and lipid-like molecules | Fatty Acyls | Fatty acids and conjugates | Level 2 | C22H30O3 | 1.518585458 | -1.027795272 | 0.490459096 | Down | 0.005860082 | 0.085912542 |
| 159 | D-Tyrosine | Organic acids and derivatives | Carboxylic acids and derivatives | Amino acids, peptides, and analogues | Level 1 | C9H11NO3 | 1.060945542 | -0.497574478 | 0.7082966 | Down | 0.005867094 | 0.085912542 |
| 160 | O-hexanoyl-adenosine monophosphate | Nucleosides, nucleotides, and analogues | Purine nucleotides | Purine ribonucleotides | Level 3 | C16H24N5O8P | 1.566615101 | 1.157988723 | 2.231461202 | Up | 0.005889611 | 0.085912542 |
| 161 | TG(10:0/8:0/i-16:0) | Lipids and lipid-like molecules | Glycerolipids | Triradylglycerols | Level 3 | C37H70O6 | 1.368632284 | -0.850586197 | 0.554559361 | Down | 0.005905372 | 0.085912542 |
| 162 | xi-Linalool 3-[rhamnosyl-(1->6)-glucoside] | Lipids and lipid-like molecules | Fatty Acyls | Fatty acyl glycosides | Level 2 | C22H38O10 | 4.064946315 | 7.127464053 | 139.8235979 | Up | 0.005923217 | 0.085912542 |
| 163 | (2E,4E,7E)-Decatrienoylcarnitine | Lipids and lipid-like molecules | Fatty Acyls | Fatty acid esters | Level 4 | C17H27NO4 | 1.051757223 | 0.512260034 | 1.42628277 | Up | 0.005969841 | 0.085912542 |
| 164 | Glycyl-Cysteine | Organic acids and derivatives | Carboxylic acids and derivatives | Amino acids, peptides, and analogues | Level 4 | C5H10N2O3S | 1.530984294 | 1.04127285 | 2.058042606 | Up | 0.005980632 | 0.085912542 |
| 165 | Berkeleylactone M | Phenylpropanoids and polyketides | Macrolides and analogues | Unclassified | Level 2 | C20H34O7 | 1.736802477 | -1.434560259 | 0.369959626 | Down | 0.006091682 | 0.085912542 |
| 166 | L-Proline | Organic acids and derivatives | Carboxylic acids and derivatives | Amino acids, peptides, and analogues | Level 1 | C5H9NO2 | 1.174594987 | -0.607888849 | 0.656156178 | Down | 0.006142818 | 0.085912542 |
| 167 | Spermine | Organic nitrogen compounds | Organonitrogen compounds | Amines | Level 1 | C10H26N4 | 2.656591511 | 3.165402447 | 8.971831013 | Up | 0.006173654 | 0.085912542 |
| 168 | Symmetric dimethylarginine | Organic acids and derivatives | Carboxylic acids and derivatives | Amino acids, peptides, and analogues | Level 2 | C8H18N4O2 | 0.790773478 | -0.288644412 | 0.818670938 | Down | 0.006194992 | 0.085912542 |
| 169 | 1-Adamantanecarbonyl-RF-NH2 | Organic acids and derivatives | Carboxylic acids and derivatives | Amino acids, peptides, and analogues | Level 4 | C26H38N6O3 | 3.649409522 | 5.862207678 | 58.17017255 | Up | 0.006215714 | 0.085912542 |
| 170 | 7(14)-Farnesene-9,12-diol | Lipids and lipid-like molecules | Prenol lipids | Sesquiterpenoids | Level 2 | C15H30O2 | 1.408838409 | 0.895650971 | 1.860449166 | Up | 0.006230806 | 0.085912542 |
| 171 | (Z)-Stellettic acid B | Lipids and lipid-like molecules | Fatty Acyls | Fatty acids and conjugates | Level 2 | C20H32O3 | 1.366083501 | -0.828894547 | 0.562960441 | Down | 0.006272368 | 0.085912542 |
| 172 | DG(16:0/20:5-3OH/0:0) | Lipids and lipid-like molecules | Fatty Acyls | Eicosanoids | Level 2 | C39H66O8 | 1.26919851 | -0.71776803 | 0.6080374 | Down | 0.006302317 | 0.085912542 |
| 173 | MG(20:3/0:0/0:0) | Lipids and lipid-like molecules | Glycerolipids | Monoradylglycerols | Level 2 | C23H40O4 | 1.188468038 | 0.671577575 | 1.592813745 | Up | 0.006374387 | 0.085912542 |
| 174 | SCHEMBL11973504 | Lipids and lipid-like molecules | Fatty Acyls | Fatty acid esters | Level 4 | C21H41NO5 | 1.785019719 | 1.361435388 | 2.569406918 | Up | 0.006385004 | 0.085912542 |
| 175 | Lanthionine ketimine | Organic acids and derivatives | Carboxylic acids and derivatives | Amino acids, peptides, and analogues | Level 2 | C6H7NO4S | 3.423114338 | -5.007542903 | 0.031087041 | Down | 0.006395954 | 0.085912542 |
| 176 | Cinncassiol D4 | Lipids and lipid-like molecules | Prenol lipids | Sesquiterpenoids | Level 1 | C20H32O5 | 1.340379425 | 0.838372809 | 1.788032313 | Up | 0.006407419 | 0.085912542 |
| 177 | Propyl 1-(propylsulfinyl)propyl disulfide | Organosulfur compounds | Sulfoxides | Unclassified | Level 2 | C9H20OS3 | 1.17438977 | 0.611393836 | 1.527734491 | Up | 0.006448015 | 0.085912542 |
| 178 | 3-Acetoxystearic acid | Lipids and lipid-like molecules | Fatty Acyls | Fatty acids and conjugates | Level 2 | C20H38O4 | 0.90168407 | -0.362103887 | 0.778029149 | Down | 0.006506062 | 0.085912542 |
| 179 | Verrucosanene | Lipids and lipid-like molecules | Prenol lipids | Diterpenoids | Level 2 | C20H32 | 1.416484621 | -0.893526183 | 0.538296823 | Down | 0.006511266 | 0.085912542 |
| 180 | 3-Methyl-3-butenyl hexadecanoate | Lipids and lipid-like molecules | Fatty Acyls | Fatty acid esters | Level 2 | C21H40O2 | 1.402830882 | 0.91539981 | 1.886091687 | Up | 0.006620147 | 0.086866574 |
| 181 | (12Z,15Z)-10-Hydroxyoctadeca-12,15-dienoylcarnitine | Lipids and lipid-like molecules | Fatty Acyls | Fatty acid esters | Level 1 | C25H45NO5 | 1.105184787 | 0.609872118 | 1.526123926 | Up | 0.00671148 | 0.086998358 |
| 182 | Mizolastine | Organoheterocyclic compounds | Benzimidazoles | Unclassified | Level 4 | C24H25FN6O | 1.073399126 | 0.529578187 | 1.443507083 | Up | 0.006751452 | 0.086998358 |
| 183 | PC(O-18:0/14:1) | Lipids and lipid-like molecules | Glycerophospholipids | Glycerophosphocholines | Level 1 | C40H80NO7P | 2.642700365 | -3.040706282 | 0.121522362 | Down | 0.006776557 | 0.086998358 |
| 184 | Argininosuccinic acid | Organic acids and derivatives | Carboxylic acids and derivatives | Amino acids, peptides, and analogues | Level 2 | C10H18N4O6 | 1.356794203 | -0.842526528 | 0.557666098 | Down | 0.006792841 | 0.086998358 |
| 185 | Uridine 5'-monophosphate | Nucleosides, nucleotides, and analogues | Pyrimidine nucleotides | Pyrimidine ribonucleotides | Level 1 | C9H13N2O9P | 1.140159763 | 0.63426358 | 1.552145259 | Up | 0.006843652 | 0.086998358 |
| 186 | Andre | Lipids and lipid-like molecules | Steroids and steroid derivatives | Steroid esters | Level 2 | C21H28O3 | 1.589654448 | 1.142057725 | 2.206955783 | Up | 0.006849976 | 0.086998358 |
| 187 | 4-[(2,4-Dihydroxy-3,3-dimethylbutanoyl)amino]butanoic acid | Organic acids and derivatives | Carboxylic acids and derivatives | Amino acids, peptides, and analogues | Level 2 | C10H19NO5 | 1.572519372 | -1.187971716 | 0.438919504 | Down | 0.00689479 | 0.087101729 |
| 188 | delta10-6-IsoF | Lipids and lipid-like molecules | Fatty Acyls | Fatty alcohols | Level 1 | C20H34O6 | 1.284934122 | 0.779106459 | 1.716067688 | Up | 0.00705837 | 0.088238646 |
| 189 | 3H-Dopamine | Organic oxygen compounds | Organooxygen compounds | Alcohols and polyols | Level 2 | C8H13NO2 | 1.414154856 | -0.880794961 | 0.543068105 | Down | 0.007059092 | 0.088238646 |
| 190 | 12-Hydroxyheptadeca-5,8,10-trienoic acid | Lipids and lipid-like molecules | Fatty Acyls | Fatty acids and conjugates | Level 4 | C17H28O3 | 1.462931348 | 0.958803178 | 1.943696786 | Up | 0.007149662 | 0.088902862 |
| 191 | L-Methionine | Organic acids and derivatives | Carboxylic acids and derivatives | Amino acids, peptides, and analogues | Level 1 | C5H11NO2S | 1.113340617 | -0.548581107 | 0.68369221 | Down | 0.007237362 | 0.089273133 |
| 192 | 5,6-Dihydroxyindole-2-carboxylic acid | Organoheterocyclic compounds | Indoles and derivatives | Indolecarboxylic acids and derivatives | Level 2 | C9H7NO4 | 0.987552949 | -0.447485594 | 0.733319804 | Down | 0.007254617 | 0.089273133 |
| 193 | 2-(L-Menthoxy)ethanol | Lipids and lipid-like molecules | Prenol lipids | Monoterpenoids | Level 4 | C12H24O2 | 2.521862717 | 2.922699007 | 7.582633573 | Up | 0.0074483 | 0.091184085 |
| 194 | Dihydroactinidiolide | Organoheterocyclic compounds | Benzofurans | Unclassified | Level 1 | C11H16O2 | 1.103534418 | 0.606089506 | 1.522127812 | Up | 0.007575601 | 0.091691308 |
| 195 | Valylserine | Organic acids and derivatives | Carboxylic acids and derivatives | Amino acids, peptides, and analogues | Level 2 | C8H16N2O4 | 0.995339092 | 0.493740061 | 1.4080905 | Up | 0.007657563 | 0.091691308 |
| 196 | Thioxanthine monophosphate | Organoheterocyclic compounds | Imidazopyrimidines | Purines and purine derivatives | Level 2 | C5H5N4O5PS | 1.361923689 | -0.835011108 | 0.560578723 | Down | 0.007665661 | 0.091691308 |
| 197 | 6-Phosphonoglucono-D-lactone | Organic oxygen compounds | Organooxygen compounds | Carbohydrates and carbohydrate conjugates | Level 2 | C6H11O9P | 1.057995579 | -0.561535132 | 0.677580785 | Down | 0.007723764 | 0.091691308 |
| 198 | 8,15-diHETE(n-3) | Lipids and lipid-like molecules | Fatty Acyls | Eicosanoids | Level 2 | C20H32O4 | 2.067147404 | -2.100583915 | 0.233163858 | Down | 0.007727662 | 0.091691308 |

| | | | | | | | | | | | | |
|---|---|---|---|---|---|---|---|---|---|---|---|---|
| 199 | Ethylene glycol distearate | Lipids and lipid-like molecules | Fatty Acyls | Fatty acid esters | Level 4 | C38H74O4 | 1.082125001 | -0.538441365 | 0.688514353 | Down | 0.007744738 | 0.091691308 |
| 200 | Neoraucarpan | Phenylpropanoids and polyketides | Isoflavonoids | Furanoisoflavonoids | Level 4 | C23H24O6 | 1.017407327 | 0.480230855 | 1.394966866 | Up | 0.00775998 | 0.091691308 |
| 201 | N,N,N-Trimethylethenaminium | Organic nitrogen compounds | Organonitrogen compounds | Quaternary ammonium salts | Level 4 | C5H12N+ | 1.185954622 | -0.626745248 | 0.647635849 | Down | 0.007800993 | 0.091719601 |
| 202 | L-Fucose | Organic oxygen compounds | Organooxygen compounds | Carbohydrates and carbohydrate conjugates | Level 2 | C6H12O5 | 1.22730586 | -0.768667028 | 0.586959543 | Down | 0.008013198 | 0.093750466 |
| 203 | S-Methylthiocitrulline | Organic acids and derivatives | Carboxylic acids and derivatives | Amino acids, peptides, and analogues | Level 4 | C7H15N3O2S | 1.194529422 | -0.649714299 | 0.637406529 | Down | 0.008179108 | 0.095222453 |
| 204 | 13,14-dihydro-15-keto Prostaglandin J2 | Lipids and lipid-like molecules | Fatty Acyls | Eicosanoids | Level 2 | C20H30O4 | 1.279744081 | -0.74335466 | 0.597348737 | Down | 0.008432377 | 0.097692168 |
| 205 | 3-Methyltetradecanoylcarnitine | Lipids and lipid-like molecules | Fatty Acyls | Fatty acid esters | Level 4 | C22H43NO4 | 1.058015268 | 0.554245319 | 1.468400305 | Up | 0.008756438 | 0.100954079 |
| 206 | S-Allylcysteine | Organic acids and derivatives | Carboxylic acids and derivatives | Amino acids, peptides, and analogues | Level 2 | C6H11NO2S | 2.778710054 | -3.5721634 | 0.084075928 | Down | 0.008837057 | 0.101391356 |
| 207 | (2E,7E)-Nona-2,7-dienedioylcarnitine | Lipids and lipid-like molecules | Fatty Acyls | Fatty acid esters | Level 2 | C16H25NO6 | 1.86784003 | 1.642070222 | 3.121133835 | Up | 0.008966081 | 0.101729335 |
| 208 | 6-Octenoylcarnitine | Lipids and lipid-like molecules | Fatty Acyls | Fatty acid esters | Level 1 | C15H27NO4 | 1.273847227 | 0.777173234 | 1.713769681 | Up | 0.009010945 | 0.101729335 |
| 209 | gamma-L-Glutamyl-L-pipecolic acid | Organic acids and derivatives | Carboxylic acids and derivatives | Amino acids, peptides, and analogues | Level 2 | C11H18N2O5 | 1.255100836 | -0.712953302 | 0.610070002 | Down | 0.009019836 | 0.101729335 |
| 210 | (9Z,12Z,15Z)-17-Hydroxyoctadeca-9,12,15-trienoylcarnitine | Lipids and lipid-like molecules | Fatty Acyls | Fatty acid esters | Level 1 | C25H43NO5 | 1.366572114 | 0.905270054 | 1.872895044 | Up | 0.009037848 | 0.101729335 |
| 211 | Octenoyl-L-carnitine | Organic acids and derivatives | Keto acids and derivatives | Medium-chain keto acids and derivatives | Level 1 | C15H27NO4 | 1.249331899 | 0.736044813 | 1.665603278 | Up | 0.009099864 | 0.101944226 |
| 212 | PA(16:0/20:4) | Lipids and lipid-like molecules | Glycerophospholipids | Glycerophosphates | Level 2 | C39H69O8P | 1.216341742 | -0.703082475 | 0.614258374 | Down | 0.009147906 | 0.1020013 |
| 213 | 1-O-(2R-hydroxy-pentadecyl)-sn-glycerol | Lipids and lipid-like molecules | Fatty Acyls | Fatty alcohols | Level 3 | C18H38O4 | 0.919967324 | 0.423825202 | 1.34147968 | Up | 0.009272048 | 0.102424115 |
| 214 | 4-Oxododecanedioic acid | Organic acids and derivatives | Keto acids and derivatives | Medium-chain keto acids and derivatives | Level 2 | C12H20O5 | 1.074987718 | 0.553707559 | 1.467853065 | Up | 0.009272078 | 0.102424115 |
| 215 | Cysteinylglycine | Organic acids and derivatives | Carboxylic acids and derivatives | Amino acids, peptides, and analogues | Level 2 | C5H10N2O3S | 1.560428617 | 1.119973151 | 2.173429277 | Up | 0.009350879 | 0.102786203 |
| 216 | Decenoylcarnitine | Lipids and lipid-like molecules | Fatty Acyls | Fatty acids and conjugates | Level 1 | C17H31NO4 | 1.591356229 | 1.253557063 | 2.384285608 | Up | 0.009391413 | 0.102786203 |
| 217 | (+/-)-8-Hydroxy-5Z,9E,11Z,14Z,17Z-eicosapentaenoic acid | Lipids and lipid-like molecules | Fatty Acyls | Eicosanoids | Level 1 | C20H30O3 | 1.04373674 | 0.517482968 | 1.431455645 | Up | 0.009466335 | 0.103130939 |
| 218 | 3S-methyl-2-oxo-pentanoic acid | Organic acids and derivatives | Keto acids and derivatives | Short-chain keto acids and derivatives | Level 2 | C6H10O3 | 1.305792146 | 0.834080401 | 1.782720339 | Up | 0.009588722 | 0.103275165 |
| 219 | Panaquinquecol 4 | Organic oxygen compounds | Organooxygen compounds | Carbonyl compounds | Level 4 | C17H22O3 | 1.008152499 | 0.522142908 | 1.436086757 | Up | 0.009635713 | 0.103275165 |
| 220 | 1,4-Dihydroxynonane | Lipids and lipid-like molecules | Fatty Acyls | Fatty alcohols | Level 4 | C9H20O2 | 2.526743917 | 3.17586202 | 9.037113361 | Up | 0.009683076 | 0.103275165 |
| 221 | beta-Farnesene | Lipids and lipid-like molecules | Prenol lipids | Sesquiterpenoids | Level 2 | C15H24 | 1.050118002 | 0.534754117 | 1.448695228 | Up | 0.009692842 | 0.103275165 |
| 222 | 8,11,14-octadecatriynoic acid | Lipids and lipid-like molecules | Fatty Acyls | Fatty acids and conjugates | Level 2 | C18H24O2 | 1.145292791 | 0.642923533 | 1.561490219 | Up | 0.009712948 | 0.103275165 |
| 223 | Dihydroceramide | Organic acids and derivatives | Carboxylic acids and derivatives | Carboxylic acid derivatives | Level 3 | C19H39NO3 | 1.956391716 | 1.760352481 | 3.387808861 | Up | 0.009740479 | 0.103275165 |
| 224 | LysoPG(18:3/0:0) | Lipids and lipid-like molecules | Glycerophospholipids | Glycerophosphoglycerols | Level 2 | C24H43O9P | 1.019104135 | 0.506074218 | 1.420180417 | Up | 0.009965267 | 0.105188925 |
| 225 | Coumarinic acid | Phenylpropanoids and polyketides | Cinnamic acids and derivatives | Hydroxycinnamic acids and derivatives | Level 3 | C9H8O3 | 1.101597371 | -0.545649695 | 0.685082817 | Down | 0.010113603 | 0.105500407 |
| 226 | Amaronol A | Phenylpropanoids and polyketides | Aurone flavonoids | Auronols | Level 2 | C15H12O8 | 2.440578664 | 2.649058589 | 6.272578364 | Up | 0.010139208 | 0.105500407 |
| 227 | Norvaline | Organic acids and derivatives | Carboxylic acids and derivatives | Amino acids, peptides, and analogues | Level 1 | C5H11NO2 | 1.047483177 | -0.493171329 | 0.710461645 | Down | 0.010144308 | 0.105500407 |
| 228 | (8Z,11Z,14Z,17Z)-Icosa-8,11,14,17-tetraenoylcarnitine | Lipids and lipid-like molecules | Fatty Acyls | Fatty acid esters | Level 4 | C27H45NO4 | 1.057880032 | 0.554831185 | 1.46899673 | Up | 0.01017246 | 0.105500407 |
| 229 | Royal Jelly acid | Organic acids and derivatives | Hydroxy acids and derivatives | Medium-chain hydroxy acids and derivatives | Level 2 | C10H18O3 | 1.623043112 | 1.281222188 | 2.430447866 | Up | 0.010566087 | 0.109106331 |
| 230 | a-L-threo-4-Hex-4-enopyranuronosyl-D-galacturonic acid | Organic oxygen compounds | Organooxygen compounds | Carbohydrates and carbohydrate conjugates | Level 2 | C12H16O12 | 1.188035496 | -0.65966692 | 0.633024429 | Down | 0.01083881 | 0.110718411 |
| 231 | Homo-L-arginine | Organic acids and derivatives | Carboxylic acids and derivatives | Amino acids, peptides, and analogues | Level 2 | C7H16N4O2 | 1.311291107 | -0.813471738 | 0.569010927 | Down | 0.010844685 | 0.110718411 |
| 232 | 2S-hydroxy-3-(10Z-tetradecenoyloxy)-propanoic acid | Organic oxygen compounds | Organooxygen compounds | Carbohydrates and carbohydrate conjugates | Level 2 | C17H30O5 | 2.048646399 | 2.039849582 | 4.112026557 | Up | 0.010889816 | 0.110718411 |
| 233 | o6-Benzyl-8-oxoguanine | Organoheterocyclic compounds | Imidazopyrimidines | Purines and purine derivatives | Level 4 | C12H11N5O2 | 3.029644315 | 4.24997753 | 19.02701749 | Up | 0.010908677 | 0.110718411 |
| 234 | D-Erythrose 4-phosphate | Organic oxygen compounds | Organooxygen compounds | Carbohydrates and carbohydrate conjugates | Level 2 | C4H9O7P | 2.617613389 | 3.127312579 | 8.738057378 | Up | 0.010964152 | 0.110807917 |
| 235 | PE(O-18:1/0:0) | Lipids and lipid-like molecules | Glycerophospholipids | Glycerophosphoethanolamines | Level 1 | C23H48NO6P | 1.045454436 | -0.547799129 | 0.68406289 | Down | 0.011058704 | 0.111289919 |
| 236 | Avenanthramide K | Phenylpropanoids and polyketides | Cinnamic acids and derivatives | Hydroxycinnamic acids and derivatives | Level 4 | C16H13NO6 | 1.396246962 | -0.851504619 | 0.55420644 | Down | 0.01114642 | 0.111699354 |
| 237 | Tridec-11-enedioylcarnitine | Lipids and lipid-like molecules | Fatty Acyls | Fatty acid esters | Level 4 | C20H35NO6 | 1.030801477 | 0.542372378 | 1.456365407 | Up | 0.011225564 | 0.111989959 |
| 238 | ent-7-D4t-NeuroP | Lipids and lipid-like molecules | Fatty Acyls | Fatty acids and conjugates | Level 1 | C22H32O5 | 1.734201089 | 1.460936464 | 2.752869964 | Up | 0.011269726 | 0.111989959 |
| 239 | PA(20:3/15:0) | Lipids and lipid-like molecules | Glycerophospholipids | Glycerophosphates | Level 4 | C38H69O8P | 3.614787389 | -5.819869146 | 0.017702916 | Down | 0.01139339 | 0.112358731 |
| 240 | Medrogestone | Lipids and lipid-like molecules | Steroids and steroid derivatives | Pregnane steroids | Level 4 | C23H32O2 | 1.134495119 | 0.603909357 | 1.519829364 | Up | 0.011465021 | 0.112358731 |
| 241 | 4-(2-Hydroxyethyl)-1-piperazineethanesulfonic acid | Organoheterocyclic compounds | Diazinanes | Piperazines | Level 2 | C8H18N2O4S | 1.752549242 | -1.424791019 | 0.372473316 | Down | 0.011481565 | 0.112358731 |
| 242 | Geranylgeraniol | Lipids and lipid-like molecules | Prenol lipids | Diterpenoids | Level 1 | C20H34O | 1.294625473 | 0.823586621 | 1.769800351 | Up | 0.011565028 | 0.112358731 |
| 243 | Thiomorpholine | Organoheterocyclic compounds | Thiazinanes | Thiomorpholines | Level 4 | C4H9NS | 1.125392521 | -0.579970654 | 0.668977385 | Down | 0.011573661 | 0.112358731 |
| 244 | Thioproline | Organic acids and derivatives | Carboxylic acids and derivatives | Amino acids, peptides, and analogues | Level 2 | C4H7NO2S | 1.842058136 | 1.555390481 | 2.939132668 | Up | 0.01159069 | 0.112358731 |
| 245 | 17-octadecenoic acid | Lipids and lipid-like molecules | Fatty Acyls | Fatty acids and conjugates | Level 2 | C18H34O2 | 1.444042165 | -1.019098665 | 0.493424527 | Down | 0.011755427 | 0.11246906 |
| 246 | Uridine 2'-phosphate | Organic oxygen compounds | Organooxygen compounds | Carbohydrates and carbohydrate conjugates | Level 2 | C9H13N2O9P | 4.069813036 | 7.498255962 | 180.8006384 | Up | 0.011823007 | 0.11246906 |
| 247 | gamma-Glutamylmethionine | Organic acids and derivatives | Carboxylic acids and derivatives | Amino acids, peptides, and analogues | Level 2 | C10H18N2O5S | 1.429076303 | -0.93356153 | 0.523564241 | Down | 0.011859116 | 0.11246906 |
| 248 | 1,3-Bis(hydroxymethyl)-3-methylbicyclo[2.2.1]heptan-2-one | Lipids and lipid-like molecules | Prenol lipids | Monoterpenoids | Level 4 | C10H16O3 | 1.455848662 | 1.027905853 | 2.039062296 | Up | 0.011859498 | 0.11246906 |
| 249 | N,N-Diethylglycine | Organic acids and derivatives | Carboxylic acids and derivatives | Amino acids, peptides, and analogues | Level 1 | C6H13NO2 | 1.218867467 | -0.691003674 | 0.619422772 | Down | 0.011895197 | 0.11246906 |
| 250 | FAHFA(12:1/3-O-8:0) | Lipids and lipid-like molecules | Fatty Acyls | Fatty acids and conjugates | Level 4 | C20H36O4 | 0.779214905 | 0.313318325 | 1.242562418 | Up | 0.011988061 | 0.11246906 |
| 251 | Malonylcarnitine | Lipids and lipid-like molecules | Fatty Acyls | Fatty acid esters | Level 2 | C10H17NO6 | 0.922288078 | 0.417356088 | 1.335477887 | Up | 0.012021507 | 0.11246906 |
| 252 | Cyclic AMP | Nucleosides, nucleotides, and analogues | Purine nucleotides | Cyclic purine nucleotides | Level 2 | C10H12N5O6P | 1.112069601 | 0.634267544 | 1.552149524 | Up | 0.01202827 | 0.11246906 |
| 253 | Dimethyl pimelimidate | Organic acids and derivatives | Carboximidic acids and derivatives | Imidoesters | Level 3 | C9H18N2O2 | 2.645223472 | -3.458110038 | 0.090992406 | Down | 0.012106229 | 0.11252598 |
| 254 | LysoPE(17:0/0:0) | Lipids and lipid-like molecules | Glycerophospholipids | Glycerophosphoethanolamines | Level 1 | C22H46NO7P | 1.04532842 | -0.532893409 | 0.691167167 | Down | 0.012129116 | 0.11252598 |
| 255 | 1,4,7,10,13,16-Hexaoxacyclooctadecane | Organic oxygen compounds | Organooxygen compounds | Ethers | Level 2 | C12H24O6 | 1.37252903 | 0.900860688 | 1.86717958 | Up | 0.012290137 | 0.113576173 |
| 256 | Sedoheptulose 1,7-bisphosphate | Organic oxygen compounds | Organooxygen compounds | Carbohydrates and carbohydrate conjugates | Level 2 | C7H16O13P2 | 1.615358701 | -1.275696464 | 0.413025722 | Down | 0.012392926 | 0.113933668 |
| 257 | 10,14-octadecadiynoic acid | Lipids and lipid-like molecules | Fatty Acyls | Fatty acids and conjugates | Level 1 | C18H28O2 | 1.276191398 | 0.843703979 | 1.794651826 | Up | 0.012424766 | 0.113933668 |
| 258 | Prenylcysteine | Organic acids and derivatives | Carboxylic acids and derivatives | Amino acids, peptides, and analogues | Level 4 | C8H15NO2S | 3.248509436 | -4.857845925 | 0.034485987 | Down | 0.012555856 | 0.114692912 |
| 259 | Cinncassiol D3 | Lipids and lipid-like molecules | Prenol lipids | Sesquiterpenoids | Level 4 | C20H32O6 | 1.402330336 | 0.953950451 | 1.937169846 | Up | 0.012654898 | 0.115154725 |
| 260 | 5S,6S-DiHETE | Lipids and lipid-like molecules | Fatty Acyls | Eicosanoids | Level 4 | C20H32O4 | 1.770883581 | 1.539482371 | 2.906901871 | Up | 0.012750365 | 0.115580601 |
| 261 | 5S-Hp-18S-HEPE | Lipids and lipid-like molecules | Fatty Acyls | Eicosanoids | Level 3 | C20H30O5 | 1.219887285 | 0.734045587 | 1.663296754 | Up | 0.01296658 | 0.116244139 |
| 262 | N-Acetyl-L-alanine | Organic acids and derivatives | Carboxylic acids and derivatives | Amino acids, peptides, and analogues | Level 2 | C5H9NO3 | 1.088023078 | -0.587777279 | 0.665367231 | Down | 0.012970358 | 0.116244139 |
| 263 | Pregnanetriolone | Lipids and lipid-like molecules | Steroids and steroid derivatives | Pregnane steroids | Level 2 | C21H34O4 | 1.153791049 | 0.706064881 | 1.63134835 | Up | 0.013041293 | 0.116244139 |
| 264 | N-Acetyl-D-Glucosamine 6-Phosphate | Organic oxygen compounds | Organooxygen compounds | Carbohydrates and carbohydrate conjugates | Level 1 | C8H16NO9P | 1.176921066 | -0.756296935 | 0.592013945 | Down | 0.013051887 | 0.116244139 |

| | | | | | | | | | | | | |
|---|---|---|---|---|---|---|---|---|---|---|---|---|
| 265 | Leucylproline | Organic acids and derivatives | Carboxylic acids and derivatives | Amino acids, peptides, and analogues | Level 1 | C11H20N2O3 | 1.158833864 | -0.645668068 | 0.63919673 | Down | 0.013068288 | 0.116244139 |
| 266 | Muridienin 3 | Lipids and lipid-like molecules | Fatty Acyls | Fatty alcohols | Level 4 | C37H66O2 | 0.943751904 | -0.451289586 | 0.731388787 | Down | 0.013263887 | 0.117543777 |
| 267 | Agelin | Lipids and lipid-like molecules | Steroids and steroid derivatives | Pregnane steroids | Level 3 | C24H29ClO4 | 1.348715518 | 0.873968736 | 1.832697573 | Up | 0.01341778 | 0.118411402 |
| 268 | Allysine | Organic acids and derivatives | Carboxylic acids and derivatives | Amino acids, peptides, and analogues | Level 2 | C6H11NO3 | 0.958603705 | 0.450733293 | 1.366734764 | Up | 0.013463262 | 0.118411402 |
| 269 | Glycylserylprolylmethionylphenylalanylvalinamide | Organic acids and derivatives | Carboxylic acids and derivatives | Amino acids, peptides, and analogues | Level 3 | C29H45N7O7S | 2.948460518 | -3.899295118 | 0.067018578 | Down | 0.013534927 | 0.118411402 |
| 270 | 3-Hydroxydodeca-5,7-dienoylcarnitine | Lipids and lipid-like molecules | Fatty Acyls | Fatty acid esters | Level 2 | C19H33NO5 | 1.506075992 | 1.087445631 | 2.124974651 | Up | 0.013561222 | 0.118411402 |
| 271 | 6-[5]-ladderane-1-hexanol | Lipids and lipid-like molecules | Fatty Acyls | Fatty alcohols | Level 2 | C18H28O | 1.475227047 | 1.043617229 | 2.061389644 | Up | 0.013615091 | 0.118446303 |
| 272 | Phoxim | Benzenoids | Benzene and substituted derivatives | Unclassified | Level 4 | C12H15N2O3PS | 1.23603776 | 0.73044817 | 1.659154424 | Up | 0.013734313 | 0.118939456 |
| 273 | 4-Hydroxynonenal glutathione | Organic acids and derivatives | Carboxylic acids and derivatives | Amino acids, peptides, and analogues | Level 1 | C19H33N3O8S | 1.530803038 | 1.113728915 | 2.164042624 | Up | 0.013865411 | 0.118998853 |
| 274 | Cucurbic acid | Lipids and lipid-like molecules | Fatty Acyls | Lineolic acids and derivatives | Level 2 | C12H20O3 | 1.62748251 | 1.28466772 | 2.436259345 | Up | 0.013879024 | 0.118998853 |
| 275 | 2',3'-Dideoxycytidine-5'-monophosphate | Organoheterocyclic compounds | Diazines | Pyrimidines and pyrimidine derivatives | Level 4 | C9H14N3O6P | 1.243437635 | 0.725194993 | 1.653124056 | Up | 0.013990618 | 0.119315525 |
| 276 | Mesterolone | Lipids and lipid-like molecules | Steroids and steroid derivatives | Androstane steroids | Level 4 | C20H32O2 | 1.000181335 | 0.518977068 | 1.432938873 | Up | 0.014016434 | 0.119315525 |
| 277 | (3S)-3-hydroxycyclocitral | Organic oxygen compounds | Organooxygen compounds | Alcohols and polyols | Level 3 | C10H16O2 | 0.906102535 | 0.428159945 | 1.345516371 | Up | 0.014221977 | 0.120632843 |
| 278 | 6-Butyryl-5-hydroxy-4-phenylseselin | Phenylpropanoids and polyketides | Neoflavonoids | Prenylated neoflavonoids | Level 4 | C24H22O5 | 1.109435654 | 0.607310883 | 1.523416981 | Up | 0.014303423 | 0.120891919 |
| 279 | 1-(2-methoxy-docosanyl)-sn-glycero-3-phosphoethanolamine | Lipids and lipid-like molecules | Glycerophospholipids | Glycerophosphoethanolamines | Level 4 | C28H60NO7P | 0.994298375 | -0.460576723 | 0.726695701 | Down | 0.014356055 | 0.120906494 |
| 280 | 15S-hydroperoxy-PGE2 | Lipids and lipid-like molecules | Fatty Acyls | Eicosanoids | Level 1 | C20H32O6 | 1.10316387 | 0.634919714 | 1.552851331 | Up | 0.014422955 | 0.121040703 |
| 281 | N,N-diethyl-3-hydroxybut-2-enamide | Lipids and lipid-like molecules | Fatty Acyls | Fatty amides | Level 2 | C8H15NO2 | 0.775293109 | 0.311876374 | 1.241321117 | Up | 0.014485532 | 0.121137811 |
| 282 | Mucic acid | Organic oxygen compounds | Organooxygen compounds | Carbohydrates and carbohydrate conjugates | Level 2 | C6H10O8 | 0.923905402 | 0.45504729 | 1.370827739 | Up | 0.014688496 | 0.122404135 |
| 283 | 2-Keto-3-deoxy-D-gluconic acid | Organic acids and derivatives | Keto acids and derivatives | Medium-chain keto acids and derivatives | Level 2 | C6H10O6 | 0.89417484 | 0.417001413 | 1.335149611 | Up | 0.014815654 | 0.123032091 |
| 284 | Furagin | Organoheterocyclic compounds | Azolidines | Imidazolidines | Level 3 | C10H8N4O5 | 1.074078004 | -0.545802651 | 0.685010187 | Down | 0.014883881 | 0.123168002 |
| 285 | (2E)-2-(methoxycarbonylmethyl)but-2-enedioic acid | Organic acids and derivatives | Carboxylic acids and derivatives | Tricarboxylic acids and derivatives | Level 3 | C7H8O6 | 0.927233007 | 0.442108531 | 1.358588485 | Up | 0.015223029 | 0.125356086 |
| 286 | Conferone | Phenylpropanoids and polyketides | Coumarins and derivatives | Unclassified | Level 2 | C24H28O4 | 1.232504279 | 0.769867915 | 1.705113666 | Up | 0.015304128 | 0.125356086 |
| 287 | Talaromycin A | Organic oxygen compounds | Organooxygen compounds | Ethers | Level 1 | C12H22O4 | 1.037205761 | 0.575862201 | 1.490568008 | Up | 0.015306638 | 0.125356086 |
| 288 | Homocitric acid | Organic acids and derivatives | Carboxylic acids and derivatives | Tricarboxylic acids and derivatives | Level 3 | C7H10O7 | 0.823941743 | 0.344421036 | 1.269641364 | Up | 0.015496834 | 0.126477597 |
| 289 | Acar(19:3) | Lipids and lipid-like molecules | Fatty Acyls | Fatty acid esters | Level 2 | C26H46NO4+ | 0.989241322 | 0.51508736 | 1.429080672 | Up | 0.015571862 | 0.126654701 |
| 290 | 17alpha-Estradiol | Lipids and lipid-like molecules | Steroids and steroid derivatives | Estrane steroids | Level 2 | C18H24O2 | 1.138626038 | 0.685484348 | 1.608241817 | Up | 0.015650034 | 0.126856079 |
| 291 | (8Z,11Z)-Icosa-8,11-dienoylcarnitine | Lipids and lipid-like molecules | Fatty Acyls | Fatty acid esters | Level 4 | C27H49NO4 | 0.901223379 | 0.446188257 | 1.362435807 | Up | 0.015782003 | 0.127490671 |
| 292 | 2-keto-n-caproic acid | Organic acids and derivatives | Keto acids and derivatives | Medium-chain keto acids and derivatives | Level 2 | C6H10O3 | 1.36969973 | 0.957866432 | 1.942435147 | Up | 0.015857853 | 0.127669154 |
| 293 | S-3-oxodecanoyl cysteamine | Lipids and lipid-like molecules | Fatty Acyls | Fatty acyl thioesters | Level 4 | C12H23NO2S | 3.117114845 | -4.66246972 | 0.039487238 | Down | 0.015940153 | 0.127898191 |
| 294 | 9R-HODE | Lipids and lipid-like molecules | Fatty Acyls | Lineolic acids and derivatives | Level 2 | C18H32O3 | 1.220610496 | 0.743989099 | 1.67480032 | Up | 0.016149149 | 0.129138815 |
| 295 | QUINUCLIDINE | Organoheterocyclic compounds | Quinuclidines | Unclassified | Level 4 | C7H13N | 1.646740614 | 1.341570524 | 2.534270504 | Up | 0.016221892 | 0.129285215 |
| 296 | Methyl 5-hydroxyoxindole-3-acetate | Organoheterocyclic compounds | Indoles and derivatives | Indolyl carboxylic acids and derivatives | Level 2 | C11H11NO4 | 2.678936307 | 3.367247406 | 10.31911546 | Up | 0.016285275 | 0.129356285 |
| 297 | Heptadecanoic acid | Lipids and lipid-like molecules | Fatty Acyls | Fatty acids and conjugates | Level 1 | C17H34O2 | 1.097732227 | -0.630481003 | 0.645961012 | Down | 0.016708744 | 0.131881728 |
| 298 | lycorine | Alkaloids and derivatives | Amaryllidaceae alkaloids | Lycorine-type amaryllidaceae alkaloids | Level 3 | C16H17NO4 | 2.039583263 | 2.059095307 | 4.167249004 | Up | 0.016765744 | 0.131881728 |
| 299 | 15d-PGA1 | Lipids and lipid-like molecules | Fatty Acyls | Eicosanoids | Level 1 | C20H30O3 | 1.033978119 | 0.539107749 | 1.45307357 | Up | 0.016769803 | 0.131881728 |
| 300 | Octadec-6-enoylcarnitine | Lipids and lipid-like molecules | Fatty Acyls | Fatty acid esters | Level 3 | C25H47NO4 | 1.84785382 | 1.842375112 | 3.585999064 | Up | 0.016839044 | 0.131989204 |
| 301 | Gravolenic acid | Phenylpropanoids and polyketides | Cinnamic acids and derivatives | Hydroxycinnamic acids and derivatives | Level 4 | C14H16O6 | 1.708983189 | 1.408376952 | 2.654383736 | Up | 0.016930363 | 0.13226846 |
| 302 | [(3R,4S)-1,1-Difluoro-3-(hexadecanoylamino)-4-hydroxy-4-phenylbutyl]phosphonic acid | Lipids and lipid-like molecules | Fatty Acyls | Fatty amides | Level 4 | C26H44F2NO5P | 1.362090173 | -0.979496756 | 0.507156616 | Down | 0.017301332 | 0.134194816 |
| 303 | Adenine | Organoheterocyclic compounds | Imidazopyrimidines | Purines and purine derivatives | Level 1 | C5H5N5 | 1.045821874 | 0.55923451 | 1.473487181 | Up | 0.017353481 | 0.134194816 |
| 304 | 14,15-Dehydrocrepenynic acid | Lipids and lipid-like molecules | Fatty Acyls | Fatty acids and conjugates | Level 2 | C18H28O2 | 1.151774476 | 0.712095237 | 1.638181534 | Up | 0.017398937 | 0.134194816 |
| 305 | Eicosapentaenoic acid | Lipids and lipid-like molecules | Fatty Acyls | Fatty acids and conjugates | Level 1 | C20H30O2 | 1.178994479 | -0.693705252 | 0.618263931 | Down | 0.017402949 | 0.134194816 |
| 306 | 3-Butenylcarnitine | Lipids and lipid-like molecules | Fatty Acyls | Fatty acid esters | Level 4 | C11H19NO4 | 1.004544035 | 0.540554144 | 1.4545311 | Up | 0.017667297 | 0.135792331 |
| 307 | 5-L-Glutamyl-taurine | Organic acids and derivatives | Carboxylic acids and derivatives | Amino acids, peptides, and analogues | Level 2 | C7H14N2O6S | 1.648149249 | -1.383570941 | 0.383268958 | Down | 0.017880952 | 0.136991162 |
| 308 | Allopurinol | Organoheterocyclic compounds | Pyrazolopyrimidines | Pyrazolo[3,4-d]pyrimidines | Level 4 | C5H4N4O | 0.741198989 | -0.294127437 | 0.815565449 | Down | 0.018183622 | 0.137959799 |
| 309 | MG(22:5/0:0) | Lipids and lipid-like molecules | Glycerolipids | Monoradylglycerols | Level 2 | C25H40O4 | 0.885938629 | 0.438371255 | 1.35507364 | Up | 0.018205346 | 0.137959799 |
| 310 | DG(18:0/22:6) | Lipids and lipid-like molecules | Glycerolipids | Diradylglycerols | Level 3 | C43H72O5 | 1.152403206 | -0.655526645 | 0.634843704 | Down | 0.018297826 | 0.137959799 |
| 311 | Siderol | Lipids and lipid-like molecules | Prenol lipids | Diterpenoids | Level 2 | C22H34O3 | 1.065728777 | 0.559537577 | 1.47379675 | Up | 0.018399937 | 0.138290668 |
| 312 | 2-(3,7-Dimethyl-2,6-octadienyl)-4-hydroxy-6-methoxyacetophenone | Organic oxygen compounds | Organooxygen compounds | Carbonyl compounds | Level 2 | C19H26O3 | 1.31758908 | 0.868697169 | 1.826013168 | Up | 0.018573643 | 0.138700353 |
| 313 | Crithmumdiol | Lipids and lipid-like molecules | Fatty Acyls | Fatty alcohols | Level 1 | C17H26O2 | 1.284134555 | 0.827966856 | 1.775181892 | Up | 0.01862671 | 0.138700353 |
| 314 | 10,11-dihydro-20-dihydroxy-LTB4 | Lipids and lipid-like molecules | Fatty Acyls | Eicosanoids | Level 1 | C20H34O6 | 0.792417909 | -0.312847475 | 0.805051245 | Down | 0.018647097 | 0.138700353 |
| 315 | Docosahexaenoic acid | Lipids and lipid-like molecules | Fatty Acyls | Fatty acids and conjugates | Level 1 | C22H32O2 | 0.908526181 | -0.421498179 | 0.746648858 | Down | 0.018714357 | 0.138700353 |
| 316 | 9-oxo-12Z-Octadecenoic acid | Lipids and lipid-like molecules | Fatty Acyls | Lineolic acids and derivatives | Level 1 | C18H32O3 | 1.050831817 | 0.570433509 | 1.484969716 | Up | 0.018746448 | 0.138700353 |
| 317 | Aprobarbital | Organoheterocyclic compounds | Diazines | Pyrimidines and pyrimidine derivatives | Level 3 | C10H14N2O3 | 2.040105481 | 1.935863356 | 3.826070231 | Up | 0.018866947 | 0.139158379 |
| 318 | Antcin K | Lipids and lipid-like molecules | Steroids and steroid derivatives | Bile acids, alcohols and derivatives | Level 2 | C29H44O6 | 1.082861397 | -0.610247744 | 0.655084199 | Down | 0.019202748 | 0.141196674 |
| 319 | 1-Heptadecen-3-one | Organic oxygen compounds | Organooxygen compounds | Carbonyl compounds | Level 1 | C17H34O | 1.037700877 | -0.519733562 | 0.697500636 | Down | 0.019438208 | 0.141638645 |
| 320 | 5-((8Z,11Z)-heptadeca-8,11-dien-1-yl)resorcinol | Benzenoids | Phenols | Benzenediols | Level 4 | C23H36O2 | 1.818650397 | 1.748020469 | 3.358973629 | Up | 0.019495471 | 0.141638645 |
| 321 | N2-Acetylornithine | Organic acids and derivatives | Carboxylic acids and derivatives | Amino acids, peptides, and analogues | Level 2 | C7H14N2O3 | 0.91964153 | 0.442778624 | 1.359219659 | Up | 0.019528145 | 0.141638645 |
| 322 | 4,8,12,15,19,21-tetracosahexaenoic acid | Lipids and lipid-like molecules | Fatty Acyls | Fatty acids and conjugates | Level 1 | C24H36O2 | 0.868773989 | -0.36957738 | 0.7740092 | Down | 0.019541897 | 0.141638645 |
| 323 | Octylguanidine | Organic nitrogen compounds | Organonitrogen compounds | Guanidines | Level 4 | C9H21N3 | 3.46239037 | 6.091833606 | 68.20632406 | Up | 0.019561042 | 0.141638645 |
| 324 | Alloepipregnanolone | Lipids and lipid-like molecules | Steroids and steroid derivatives | Pregnane steroids | Level 2 | C21H34O2 | 1.544823791 | 1.17618037 | 2.259776935 | Up | 0.019789341 | 0.142578815 |
| 325 | 19-Hydroxyprostaglandin E2 | Lipids and lipid-like molecules | Fatty Acyls | Eicosanoids | Level 1 | C20H32O6 | 1.255580017 | -0.825069714 | 0.564454926 | Down | 0.019851144 | 0.142578815 |
| 326 | 4-methylnonadecanoylcarnitine | Lipids and lipid-like molecules | Fatty Acyls | Fatty acid esters | Level 4 | C27H53NO4 | 0.793317609 | -0.311656509 | 0.805716101 | Down | 0.019870984 | 0.142578815 |
| 327 | Mannose 6-phosphate | Organic oxygen compounds | Organooxygen compounds | Carbohydrates and carbohydrate conjugates | Level 1 | C6H13O9P | 1.199946074 | -0.746863651 | 0.595897603 | Down | 0.019931125 | 0.142579581 |
| 328 | (2,2-Dimethyl-3-sulfooxy-3H-1-benzofuran-7-yl) N-methylcarbamate | Organoheterocyclic compounds | Coumarans | Unclassified | Level 4 | C12H15NO7S | 2.729050523 | 3.402306092 | 10.57295024 | Up | 0.020100362 | 0.142658094 |
| 329 | (-)-12-hydroxy-9,10-dihydrojasmonic acid | Lipids and lipid-like molecules | Fatty Acyls | Lineolic acids and derivatives | Level 1 | C12H20O4 | 1.006860694 | 0.561440741 | 1.475742225 | Up | 0.020117893 | 0.142658094 |
| 330 | FAHFA(2:0/2-O-24:0) | Lipids and lipid-like molecules | Fatty Acyls | Fatty acids and conjugates | Level 2 | C25H48O4 | 0.977380051 | -0.513052151 | 0.700738393 | Down | 0.0201223 | 0.142658094 |

| | | | | | | | | | | | | |
|---|---|---|---|---|---|---|---|---|---|---|---|---|
| 331 | 5'-Cytidylic acid | Nucleosides, nucleotides, and analogues | Pyrimidine nucleotides | Pyrimidine ribonucleotides | Level 1 | C9H14N3O8P | 0.972588713 | 0.509150842 | 1.423212258 | Up | 0.02026351 | 0.143081563 |
| 332 | 12S-HHT | Lipids and lipid-like molecules | Fatty Acyls | Fatty acids and conjugates | Level 1 | C17H28O3 | 1.268512007 | 0.804791441 | 1.746893239 | Up | 0.020349278 | 0.143081563 |
| 333 | Methyl 3,4-dicaffeoylquinate | Organic oxygen compounds | Organooxygen compounds | Alcohols and polyols | Level 2 | C27H28O12 | 1.060400805 | 0.584799511 | 1.499830545 | Up | 0.020563087 | 0.143081563 |
| 334 | 10-(Phosphonooxy)decyl methacrylate | Organic acids and derivatives | Organic phosphoric acids and derivatives | Phosphate esters | Level 2 | C14H27O6P | 1.044982661 | 0.610245086 | 1.526518513 | Up | 0.020568022 | 0.143081563 |
| 335 | 4-Hydroxydecanoylcarnitine | Lipids and lipid-like molecules | Fatty Acyls | Fatty acid esters | Level 2 | C17H33NO5 | 1.209321756 | 0.755504406 | 1.688221738 | Up | 0.020657716 | 0.143081563 |
| 336 | 5,6-Dihydroxyprostaglandin F1a | Lipids and lipid-like molecules | Fatty Acyls | Eicosanoids | Level 2 | C20H36O7 | 1.469687438 | -1.161122087 | 0.447164608 | Down | 0.02066399 | 0.143081563 |
| 337 | [3-[2,3-Dihydroxypropoxy(hydroxy)phosphoryl]oxy-2-hydroxypropyl] hexadecanoate | Lipids and lipid-like molecules | Glycerophospholipids | Glycerophosphoglycerols | Level 2 | C22H45O9P | 0.812652476 | 0.340191156 | 1.265924317 | Up | 0.020748262 | 0.143247447 |
| 338 | N-methyl-4,6,7-trihydroxy-1,2,3,4-tetrahydroisoquinoline | Organoheterocyclic compounds | Tetrahydroisoquinolines | Unclassified | Level 3 | C10H13NO3 | 1.138101485 | -0.655568111 | 0.634825457 | Down | 0.020921125 | 0.144022236 |
| 339 | Eicosadienoic acid | Lipids and lipid-like molecules | Fatty Acyls | Fatty acids and conjugates | Level 1 | C20H36O2 | 0.808409657 | -0.33733923 | 0.791499733 | Down | 0.021027338 | 0.144335054 |
| 340 | PA(15:0/0:0) | Lipids and lipid-like molecules | Glycerophospholipids | Glycerophosphates | Level 4 | C18H37O7P | 0.825292042 | -0.35399406 | 0.782415003 | Down | 0.021492096 | 0.147100078 |
| 341 | MGTS(18:0/0:0) | Organic acids and derivatives | Carboxylic acids and derivatives | Amino acids, peptides, and analogues | Level 1 | C28H55NO6 | 0.966221041 | 0.49293977 | 1.407309622 | Up | 0.021608989 | 0.147370355 |
| 342 | Dalterpenoid A | Lipids and lipid-like molecules | Prenol lipids | Diterpenoids | Level 3 | C20H28O3 | 1.213466752 | 0.813888139 | 1.757942813 | Up | 0.021713794 | 0.147370355 |
| 343 | p-Coumaric acid sulfate | Phenylpropanoids and polyketides | Cinnamic acids and derivatives | Cinnamic acids | Level 2 | C9H8O6S | 2.463268881 | 3.039409046 | 8.22154223 | Up | 0.021785743 | 0.147370355 |
| 344 | ETrE(5Z, 8Z, 11Z) | Lipids and lipid-like molecules | Fatty Acyls | Fatty acids and conjugates | Level 1 | C20H34O2 | 0.745659789 | -0.285637801 | 0.820378847 | Down | 0.021833393 | 0.147370355 |
| 345 | 11b,17a,21-Trihydroxypreg-nenolone | Lipids and lipid-like molecules | Steroids and steroid derivatives | Hydroxysteroids | Level 2 | C21H32O5 | 1.599391381 | 1.366811142 | 2.578998872 | Up | 0.021878388 | 0.147370355 |
| 346 | Phosphocreatinine | Organic acids and derivatives | Carboxylic acids and derivatives | Amino acids, peptides, and analogues | Level 4 | C4H8N3O4P | 0.958023888 | -0.452910736 | 0.73056739 | Down | 0.021903889 | 0.147370355 |
| 347 | 11(S)-HEDE | Lipids and lipid-like molecules | Fatty Acyls | Eicosanoids | Level 2 | C20H36O3 | 1.78148192 | 1.52539994 | 2.878665065 | Up | 0.022122502 | 0.148420738 |
| 348 | 6-(2-Hydroxyethoxy)-6-oxohexanoylcarnitine | Lipids and lipid-like molecules | Fatty Acyls | Fatty acid esters | Level 2 | C15H27NO7 | 1.505094615 | -1.117877081 | 0.46077135 | Down | 0.022444405 | 0.150156231 |
| 349 | mucic acid dimethyl ester | Organic oxygen compounds | Organooxygen compounds | Carbohydrates and carbohydrate conjugates | Level 3 | C8H14O8 | 0.824276775 | 0.375304552 | 1.297113346 | Up | 0.022532776 | 0.150323996 |
| 350 | Propiovanillone | Organic oxygen compounds | Organooxygen compounds | Carbonyl compounds | Level 4 | C10H12O3 | 0.761496746 | 0.323075962 | 1.250994947 | Up | 0.022741551 | 0.151291828 |
| 351 | Phosphopantothenic acid | Organic acids and derivatives | Carboxylic acids and derivatives | Amino acids, peptides, and analogues | Level 2 | C9H18NO8P | 1.80765791 | -1.592952829 | 0.331492279 | Down | 0.023161624 | 0.152719071 |
| 352 | Fructose 1,6-bisphosphate | Organic oxygen compounds | Organooxygen compounds | Carbohydrates and carbohydrate conjugates | Level 1 | C6H14O12P2 | 1.24957273 | -0.786421065 | 0.579780588 | Down | 0.023253239 | 0.152719071 |
| 353 | PC(16:0/2:0) | Lipids and lipid-like molecules | Glycerophospholipids | Glycerophosphocholines | Level 2 | C26H52NO8P | 1.490092679 | -1.070634446 | 0.476109577 | Down | 0.023277602 | 0.152719071 |
| 354 | Glycerylphosphorylcholine | Lipids and lipid-like molecules | Glycerophospholipids | Glycerophosphocholines | Level 3 | C8H20NO6P | 1.046103157 | 0.60152591 | 1.517320558 | Up | 0.023615039 | 0.153925158 |
| 355 | 10Z-Nonadecenoic acid | Lipids and lipid-like molecules | Fatty Acyls | Fatty acids and conjugates | Level 2 | C19H36O2 | 0.851813346 | -0.397054989 | 0.759406899 | Down | 0.023653694 | 0.153925158 |
| 356 | Isoleucyl-Phenylalanine | Organic acids and derivatives | Carboxylic acids and derivatives | Amino acids, peptides, and analogues | Level 2 | C15H22N2O3 | 1.146336821 | 0.692267274 | 1.615820875 | Up | 0.0237789 | 0.153925158 |
| 357 | Succinic acid | Organic acids and derivatives | Carboxylic acids and derivatives | Dicarboxylic acids and derivatives | Level 2 | C4H6O4 | 1.47427038 | 1.170412245 | 2.250760024 | Up | 0.023783188 | 0.153925158 |
| 358 | (-)-Stolondiol | Lipids and lipid-like molecules | Prenol lipids | Diterpenoids | Level 2 | C20H32O4 | 1.486470596 | -1.121673148 | 0.459560546 | Down | 0.023785488 | 0.153925158 |
| 359 | 4-Allylpyrocatechol sulfate | Organic acids and derivatives | Organic sulfuric acids and derivatives | Arylsulfates | Level 2 | C9H10O5S | 2.208079757 | 2.440354014 | 5.427749027 | Up | 0.024206293 | 0.15622268 |
| 360 | 5'-Methylthioadenosine | Nucleosides, nucleotides, and analogues | 5'-deoxyribonucleosides | 5'-deoxy-5'-thionucleosides | Level 1 | C11H15N5O3S | 1.049165401 | 0.615367269 | 1.531947937 | Up | 0.024466879 | 0.157476528 |
| 361 | tetrahydropyridoindole | Organoheterocyclic compounds | Quinolines and derivatives | Pyrroloquinolines | Level 4 | C11H12N2 | 3.081574127 | 5.116483954 | 34.69086609 | Up | 0.024734343 | 0.15876774 |
| 362 | Ile-Asp-Val | Organic acids and derivatives | Peptidomimetics | Hybrid peptides | Level 2 | C15H27N3O6 | 1.878206988 | -1.702505913 | 0.307251953 | Down | 0.024820719 | 0.158775053 |
| 363 | 5-Hydroxytrtradeca-7,9-dienoylcarnitine | Lipids and lipid-like molecules | Fatty Acyls | Fatty acid esters | Level 2 | C21H37NO5 | 1.639197566 | 1.42831401 | 2.691320133 | Up | 0.024869187 | 0.158775053 |
| 364 | Glucose 6-phosphate | Organic oxygen compounds | Organooxygen compounds | Carbohydrates and carbohydrate conjugates | Level 1 | C6H13O9P | 0.736662816 | -0.276042302 | 0.825853449 | Down | 0.025164142 | 0.160227445 |
| 365 | 3-Oxoheptanoylcarnitine | Lipids and lipid-like molecules | Fatty Acyls | Fatty acid esters | Level 4 | C14H25NO5 | 0.972977825 | 0.514536757 | 1.42853537 | Up | 0.025572076 | 0.162180259 |
| 366 | O-Phosphothreonine | Organic acids and derivatives | Carboxylic acids and derivatives | Amino acids, peptides, and analogues | Level 2 | C4H10NO6P | 0.715418233 | -0.278438586 | 0.824482864 | Down | 0.025613996 | 0.162180259 |
| 367 | 5-Methoxyindole-3-acetic acid | Organoheterocyclic compounds | Indoles and derivatives | Indolyl carboxylic acids and derivatives | Level 2 | C11H11NO3 | 0.691222184 | 0.263710301 | 1.200562325 | Up | 0.025786658 | 0.162449106 |
| 368 | 3-Buten-1-amine | Organic nitrogen compounds | Organonitrogen compounds | Amines | Level 1 | C4H9N | 0.897590027 | -0.39720189 | 0.759329577 | Down | 0.026217854 | 0.164685976 |
| 369 | Isopongaflavone | Phenylpropanoids and polyketides | Flavonoids | Pyranoflavonoids | Level 4 | C21H18O4 | 1.238428488 | 0.810667726 | 1.754023073 | Up | 0.026280415 | 0.164685976 |
| 370 | 4Z,7Z,9E,11E,13Z,16Z,19Z-docosaheptaenoic acid | Lipids and lipid-like molecules | Fatty Acyls | Fatty acids and conjugates | Level 1 | C22H30O2 | 0.896898827 | -0.404309295 | 0.755597957 | Down | 0.026401638 | 0.164896857 |
| 371 | N,N-Dimethylformamide dimethyl acetal | Organic acids and derivatives | Orthocarboxylic acid derivatives | Carboxylic acid amide acetals | Level 4 | C5H13NO2 | 1.846421865 | -1.852379784 | 0.276935175 | Down | 0.026452927 | 0.164896857 |
| 372 | PC(18:1/16:0) | Lipids and lipid-like molecules | Glycerophospholipids | Glycerophosphocholines | Level 3 | C42H82NO8P | 4.652335405 | -10.86319285 | 0.00053685 | Down | 0.026799954 | 0.166622752 |
| 373 | (17beta)-17-Hydroxyandrostan-3-one | Lipids and lipid-like molecules | Steroids and steroid derivatives | Androstane steroids | Level 3 | C19H30O2 | 1.308494729 | 0.906760906 | 1.874831456 | Up | 0.026941934 | 0.166779125 |
| 374 | 2-(2,2-Diphenylacetamido)-5-guanidino-N-(4-hydroxybenzyl)pentanamide | Benzenoids | Benzene and substituted derivatives | Diphenylmethanes | Level 2 | C27H31N5O3 | 2.432528032 | -2.935018085 | 0.130758978 | Down | 0.026965551 | 0.166779125 |
| 375 | Glyceryl lactooleate | Lipids and lipid-like molecules | Fatty Acyls | Fatty acid esters | Level 2 | C24H44O6 | 0.70165641 | 0.300382559 | 1.231470918 | Up | 0.027211828 | 0.167865175 |
| 376 | 17-octadecynoic acid | Lipids and lipid-like molecules | Fatty Acyls | Fatty acids and conjugates | Level 1 | C18H32O2 | 1.04358978 | -0.578916474 | 0.669466387 | Down | 0.027483217 | 0.169100107 |
| 377 | N-Acetylglutamine | Organic acids and derivatives | Carboxylic acids and derivatives | Amino acids, peptides, and analogues | Level 2 | C7H12N2O4 | 1.304361061 | -0.900182099 | 0.535819095 | Down | 0.028285725 | 0.172824886 |
| 378 | Polyporusterone E | Lipids and lipid-like molecules | Steroids and steroid derivatives | Cholestane steroids | Level 2 | C28H44O5 | 0.948550528 | 0.507829205 | 1.421909066 | Up | 0.028300872 | 0.172824886 |
| 379 | Eicosa-5,8,11,14,17-pentaenoic acid | Lipids and lipid-like molecules | Fatty Acyls | Fatty acids and conjugates | Level 2 | C20H30O2 | 1.12269612 | -0.65976777 | 0.632980179 | Down | 0.0283217 | 0.172824886 |
| 380 | Cyclopropyl-methoxycarbonyl metomidate | Organoheterocyclic compounds | Azoles | Imidazoles | Level 1 | C17H18N2O4 | 1.013065734 | 0.593545748 | 1.508950781 | Up | 0.028379665 | 0.172824886 |
| 381 | Nifekalant | Benzenoids | Benzene and substituted derivatives | Phenylpropylamines | Level 2 | C19H27N5O5 | 1.412797643 | 1.022397365 | 2.031291608 | Up | 0.028755485 | 0.174665669 |
| 382 | (4-Ethylnaphthalen-1-yl)[1-(5-fluoropentyl)-1H-indol-3-yl]methanone | Organoheterocyclic compounds | Indoles and derivatives | Naphthoylindoles | Level 3 | C26H26FNO | 1.089852322 | -0.645128156 | 0.639435987 | Down | 0.028866605 | 0.174893335 |
| 383 | MG(20:4/0:0/0:0) | Lipids and lipid-like molecules | Glycerolipids | Monoradylglycerols | Level 1 | C23H38O4 | 1.040032333 | 0.612392369 | 1.528792249 | Up | 0.0290669 | 0.175212911 |
| 384 | Hordenine | Benzenoids | Benzene and substituted derivatives | Phenethylamines | Level 2 | C10H15NO | 1.478631642 | -1.133295129 | 0.455873316 | Down | 0.029229052 | 0.175310785 |
| 385 | Sphingosine | Organic nitrogen compounds | Organonitrogen compounds | Amines | Level 1 | C18H37NO2 | 0.727727881 | -0.274316532 | 0.826841937 | Down | 0.029243739 | 0.175310785 |
| 386 | carnitine lactate | Lipids and lipid-like molecules | Fatty Acyls | Fatty acid esters | Level 4 | C10H19NO5 | 0.742795735 | 0.305059155 | 1.235469294 | Up | 0.029304582 | 0.175310785 |
| 387 | 1alpha,18,25-trihydroxyvitamin D3 | Lipids and lipid-like molecules | Steroids and steroid derivatives | Vitamin D and derivatives | Level 2 | C27H44O4 | 0.784605271 | 0.350656515 | 1.275140763 | Up | 0.029426063 | 0.175335339 |
| 388 | 2-Methyl-5-(8,11-pentadecadienyl)-1,3-benzenediol | Benzenoids | Phenols | Benzenediols | Level 2 | C22H34O2 | 0.944780797 | -0.45855135 | 0.727716612 | Down | 0.029456337 | 0.175335339 |
| 389 | LysoPC(20:3/0:0) | Lipids and lipid-like molecules | Glycerophospholipids | Glycerophosphocholines | Level 1 | C28H52NO7P | 0.960741576 | -0.468519372 | 0.722705925 | Down | 0.029571173 | 0.175578839 |
| 390 | 5-Fluorouridine | Nucleosides, nucleotides, and analogues | Pyrimidine nucleosides | Unclassified | Level 4 | C9H11FN2O6 | 1.739247039 | 1.55313914 | 2.934549695 | Up | 0.029696648 | 0.175642125 |
| 391 | Phaeophorbide b | Organoheterocyclic compounds | Tetrapyrroles and derivatives | Chlorins | Level 2 | C35H34N4O6 | 1.000163343 | 0.547434866 | 1.461484842 | Up | 0.029729741 | 0.175642125 |
| 392 | Glutamine-glutamate | Organic acids and derivatives | Carboxylic acids and derivatives | Amino acids, peptides, and analogues | Level 2 | C10H17N3O7 | 2.651898051 | 3.608921285 | 12.20094751 | Up | 0.029844957 | 0.175885292 |
| 393 | p-Coumaroyl 3-hydroxytyrosine | Organic acids and derivatives | Carboxylic acids and derivatives | Amino acids, peptides, and analogues | Level 3 | C18H17NO6 | 1.202728358 | 0.744870506 | 1.675823843 | Up | 0.030305995 | 0.17711023 |
| 394 | 11,20-dihydroxypregn-4-en-3-one | Lipids and lipid-like molecules | Steroids and steroid derivatives | Pregnane steroids | Level 3 | C21H32O3 | 1.552720759 | 1.344447404 | 2.539329138 | Up | 0.030346792 | 0.17711023 |

| | | | | | | | | | | | | |
|---|---|---|---|---|---|---|---|---|---|---|---|---|
| 395 | gamma-Linolenic acid | Lipids and lipid-like molecules | Fatty Acyls | Lineolic acids and derivatives | Level 1 | C18H30O2 | 0.779455763 | -0.345122543 | 0.787241105 | Down | 0.030457804 | 0.17711023 |
| 396 | 24-Nor-5beta-chol-22-ene-3alpha,7alpha,12alpha-triol | Lipids and lipid-like molecules | Steroids and steroid derivatives | Pregnane steroids | Level 2 | C23H38O3 | 0.856219557 | 0.390265935 | 1.310634973 | Up | 0.030458978 | 0.17711023 |
| 397 | Cytidine | Nucleosides, nucleotides, and analogues | Pyrimidine nucleosides | Unclassified | Level 1 | C9H13N3O5 | 0.720120357 | 0.305297319 | 1.235673266 | Up | 0.030498401 | 0.17711023 |
| 398 | 5-Phenyl-1,3-oxazinane-2,4-dione | Benzenoids | Benzene and substituted derivatives | Unclassified | Level 2 | C10H9NO3 | 0.797302407 | 0.345056881 | 1.270201062 | Up | 0.030500246 | 0.17711023 |
| 399 | 3-[4-(sulfooxy)phenyl]propanoic acid | Organic acids and derivatives | Organic sulfuric acids and derivatives | Arylsulfates | Level 2 | C9H10O6S | 1.880292165 | 1.757869121 | 3.381982327 | Up | 0.030683002 | 0.177287773 |
| 400 | 26:6(8Z,11Z,14Z,17Z,20Z,23Z) | Lipids and lipid-like molecules | Fatty Acyls | Fatty acids and conjugates | Level 2 | C26H40O2 | 0.913287906 | -0.441768413 | 0.736231604 | Down | 0.030695137 | 0.177287773 |
| 401 | 12-hydroxy-8E,10E-heptadecadienoic acid | Lipids and lipid-like molecules | Fatty Acyls | Fatty acids and conjugates | Level 2 | C17H30O3 | 0.987556558 | 0.526548897 | 1.440479269 | Up | 0.030754763 | 0.177287773 |
| 402 | 13,14-Dihydro-lipoxin A4 | Lipids and lipid-like molecules | Fatty Acyls | Eicosanoids | Level 2 | C20H34O5 | 1.708540992 | -1.646491651 | 0.31941597 | Down | 0.031184686 | 0.179003407 |
| 403 | 6Z,9Z,12Z,15Z,18Z-heneicosapentaenoic acid | Lipids and lipid-like molecules | Fatty Acyls | Fatty acids and conjugates | Level 2 | C21H32O2 | 1.338504867 | -1.014653769 | 0.494947096 | Down | 0.031264229 | 0.179003407 |
| 404 | Nervonyl carnitine | Lipids and lipid-like molecules | Fatty Acyls | Fatty acid esters | Level 4 | C31H60NO4+ | 0.860561048 | -0.381049733 | 0.767878664 | Down | 0.03127849 | 0.179003407 |
| 405 | cis-4-Hydroxycyclohexylacetic acid | Organic oxygen compounds | Organooxygen compounds | Alcohols and polyols | Level 2 | C8H14O3 | 0.861166177 | 0.358533202 | 1.282121693 | Up | 0.031777039 | 0.180110134 |
| 406 | Alanylphenylalanine | Organic acids and derivatives | Carboxylic acids and derivatives | Amino acids, peptides, and analogues | Level 2 | C12H16N2O3 | 1.377329098 | -0.97888247 | 0.507372605 | Down | 0.031817954 | 0.180110134 |
| 407 | Xanthurenate-8-O-beta-D-glucoside | Organic oxygen compounds | Organooxygen compounds | Carbohydrates and carbohydrate conjugates | Level 3 | C16H17NO9 | 3.125214853 | -5.484464611 | 0.022336321 | Down | 0.031819872 | 0.180110134 |
| 408 | 20-Hydroxy-PGF2a | Lipids and lipid-like molecules | Fatty Acyls | Eicosanoids | Level 2 | C20H34O6 | 1.123097777 | 0.716727117 | 1.64344949 | Up | 0.031931979 | 0.180110134 |
| 409 | Norepinephrine | Benzenoids | Phenols | Benzenediols | Level 2 | C8H11NO3 | 1.31188255 | -0.944301509 | 0.519681098 | Down | 0.031990163 | 0.180110134 |
| 410 | 8Z,11Z,14Z-octadecatrienoic acid | Lipids and lipid-like molecules | Fatty Acyls | Lineolic acids and derivatives | Level 2 | C18H30O2 | 0.804517344 | -0.360728431 | 0.778771271 | Down | 0.031997452 | 0.180110134 |
| 411 | Met-Glu-Ile | Organic acids and derivatives | Carboxylic acids and derivatives | Amino acids, peptides, and analogues | Level 2 | C16H29N3O6S1 | 1.185571089 | -0.72868484 | 0.603453771 | Down | 0.032002727 | 0.180110134 |
| 412 | Aztreonam | Organoheterocyclic compounds | Lactams | Beta lactams | Level 2 | C13H17N5O8S2 | 1.186529206 | -0.722894226 | 0.60588075 | Down | 0.032697892 | 0.18302924 |
| 413 | Palmitoleic acid | Lipids and lipid-like molecules | Fatty Acyls | Fatty acids and conjugates | Level 1 | C16H30O2 | 1.070643024 | -0.626190639 | 0.647884865 | Down | 0.032755797 | 0.18302924 |
| 414 | Cefozopran | Organoheterocyclic compounds | Lactams | Beta lactams | Level 4 | C19H17N9O5S2 | 1.004664777 | -0.522293829 | 0.69626392 | Down | 0.032861698 | 0.18302924 |
| 415 | Cer(d18:2/PGJ2) | Lipids and lipid-like molecules | Fatty Acyls | Eicosanoids | Level 4 | C38H63NO5 | 0.690179912 | 0.273554929 | 1.208782709 | Up | 0.032898833 | 0.18302924 |
| 416 | Nervonic acid | Lipids and lipid-like molecules | Fatty Acyls | Fatty acids and conjugates | Level 1 | C24H46O2 | 0.909144525 | -0.475983075 | 0.718976701 | Down | 0.032906731 | 0.18302924 |
| 417 | Rhodamine B | Organoheterocyclic compounds | Benzopyrans | 1-benzopyrans | Level 3 | C28H31N2O3+ | 1.498279829 | -1.229028276 | 0.426604688 | Down | 0.033131201 | 0.183847202 |
| 418 | Docosapentaenoic acid (22n-6) | Lipids and lipid-like molecules | Fatty Acyls | Fatty acids and conjugates | Level 1 | C22H34O2 | 0.953265775 | -0.490480121 | 0.711788179 | Down | 0.033320994 | 0.18446937 |
| 419 | 3,4-Dihydrocoumarin | Phenylpropanoids and polyketides | 3,4-dihydrocoumarins | Unclassified | Level 2 | C9H8O2 | 1.597319151 | 1.303029899 | 2.467465476 | Up | 0.033838555 | 0.186465353 |
| 420 | 9,10-Epoxy-12-octadecynoic acid | Lipids and lipid-like molecules | Fatty Acyls | Fatty acids and conjugates | Level 2 | C18H30O3 | 0.739748092 | 0.320997788 | 1.24919421 | Up | 0.033976286 | 0.186790923 |
| 421 | SCHEMBL1775151 | Lipids and lipid-like molecules | Fatty Acyls | Fatty acids and conjugates | Level 3 | C11H18O4 | 0.827552955 | 0.399408612 | 1.31896713 | Up | 0.034410787 | 0.18832251 |
| 422 | MG(14:1/0:0) | Lipids and lipid-like molecules | Glycerolipids | Monoradylglycerols | Level 1 | C17H32O4 | 1.136761072 | 0.723414473 | 1.65108509 | Up | 0.034413461 | 0.18832251 |
| 423 | Ergosterol peroxide | Lipids and lipid-like molecules | Steroids and steroid derivatives | Ergostane steroids | Level 2 | C28H44O3 | 0.713139942 | 0.305459032 | 1.235811781 | Up | 0.034530952 | 0.188336031 |
| 424 | Petasitin | Lipids and lipid-like molecules | Prenol lipids | Sesquiterpenoids | Level 2 | C20H28O4 | 0.68993303 | -0.269051002 | 0.829865247 | Down | 0.034660113 | 0.188336031 |
| 425 | gamma-Glutamylcysteinylserine | Organic acids and derivatives | Carboxylic acids and derivatives | Amino acids, peptides, and analogues | Level 1 | C11H19N3O7S | 3.556930062 | 6.310994602 | 79.39600998 | Up | 0.034726527 | 0.188336031 |
| 426 | 3-Epiaphidicolin | Lipids and lipid-like molecules | Prenol lipids | Diterpenoids | Level 2 | C20H34O4 | 1.372766509 | 1.077764559 | 2.11076294 | Up | 0.034733129 | 0.188336031 |
| 427 | Piperidine | Organoheterocyclic compounds | Piperidines | Unclassified | Level 1 | C5H11N | 1.09418427 | -0.606806829 | 0.656648479 | Down | 0.034904171 | 0.188832361 |
| 428 | Hepoxilin A3 | Lipids and lipid-like molecules | Fatty Acyls | Eicosanoids | Level 2 | C20H32O4 | 1.816874568 | -1.752169675 | 0.296855001 | Down | 0.035411209 | 0.190913983 |
| 429 | Nitro-D-arginine | Organic acids and derivatives | Carboxylic acids and derivatives | Amino acids, peptides, and analogues | Level 3 | C6H13N5O4 | 0.818458663 | 0.379457378 | 1.300852491 | Up | 0.035533878 | 0.190913983 |
| 430 | (5alpha)-23-Methyl-4-aza-21-norchol-1-ene-3,20-dione | Lipids and lipid-like molecules | Steroids and steroid derivatives | Oxosteroids | Level 4 | C23H35NO2 | 0.964831294 | 0.564159137 | 1.478525512 | Up | 0.035537929 | 0.190913983 |
| 431 | Docosapentaenoic acid (22n-3) | Lipids and lipid-like molecules | Fatty Acyls | Fatty acids and conjugates | Level 1 | C22H34O2 | 0.919983575 | -0.447819659 | 0.733150019 | Down | 0.03571836 | 0.191061048 |
| 432 | beta-Glycerophosphoric acid | Lipids and lipid-like molecules | Glycerophospholipids | Glycerophosphates | Level 2 | C3H9O6P | 0.948600195 | -0.473605523 | 0.720162547 | Down | 0.036036985 | 0.192332221 |
| 433 | Sphinganine 1-phosphate | Lipids and lipid-like molecules | Sphingolipids | Phosphosphingolipids | Level 1 | C18H40NO5P | 1.140097223 | 0.723733459 | 1.651450193 | Up | 0.036215862 | 0.192680749 |
| 434 | D-Arabino-hexos-2-ulose | Organic oxygen compounds | Organooxygen compounds | Carbohydrates and carbohydrate conjugates | Level 4 | C6H10O6 | 0.798994324 | 0.385876795 | 1.306653662 | Up | 0.036264545 | 0.192680749 |
| 435 | (5E)-8-Hydroxytetradec-5-enoylcarnitine | Lipids and lipid-like molecules | Fatty Acyls | Fatty acid esters | Level 2 | C21H39NO5 | 1.191778269 | 0.817620474 | 1.762496599 | Up | 0.036547044 | 0.193316767 |
| 436 | 13Z-hexadecenoic acid | Lipids and lipid-like molecules | Fatty Acyls | Fatty acids and conjugates | Level 2 | C16H30O2 | 1.018175404 | -0.583812921 | 0.667198097 | Down | 0.036860217 | 0.193712679 |
| 437 | Ganolucidic acid B | Lipids and lipid-like molecules | Prenol lipids | Triterpenoids | Level 2 | C30H46O6 | 2.64278171 | -4.049271306 | 0.060401521 | Down | 0.036866581 | 0.193712679 |
| 438 | PC(O-16:0/3:1) | Lipids and lipid-like molecules | Glycerophospholipids | Glycerophosphocholines | Level 1 | C27H54NO7P | 0.745728416 | -0.299319738 | 0.812635481 | Down | 0.037048686 | 0.193811957 |
| 439 | Betamethasone | Lipids and lipid-like molecules | Steroids and steroid derivatives | Hydroxysteroids | Level 2 | C22H29FO5 | 2.661215786 | 3.581820539 | 11.97389434 | Up | 0.037436537 | 0.195410495 |
| 440 | Fosfluconazole | Benzenoids | Benzene and substituted derivatives | Halobenzenes | Level 2 | C13H13F2N6O4P | 2.698924149 | 3.618354401 | 12.28098524 | Up | 0.037589878 | 0.195780614 |
| 441 | Phenylacetylglutamine | Organic acids and derivatives | Carboxylic acids and derivatives | Amino acids, peptides, and analogues | Level 2 | C13H16N2O4 | 1.815591207 | 1.675512465 | 3.194328013 | Up | 0.037787199 | 0.196118273 |
| 442 | 7-(3,4-Dimethyl-5-propylfuran-2-yl)heptanoylcarnitine | Lipids and lipid-like molecules | Fatty Acyls | Fatty acid esters | Level 2 | C23H39NO5 | 1.470862405 | 1.256495202 | 2.389146302 | Up | 0.037819861 | 0.196118273 |
| 443 | Diflucortolone | Lipids and lipid-like molecules | Steroids and steroid derivatives | Hydroxysteroids | Level 2 | C22H28F2O4 | 2.051566116 | -2.593608838 | 0.16567079 | Down | 0.037962954 | 0.196283889 |
| 444 | Thalictroidine | Organic oxygen compounds | Organooxygen compounds | Carbonyl compounds | Level 4 | C14H19NO2 | 0.972937581 | 0.570935764 | 1.485486778 | Up | 0.03801709 | 0.196283889 |
| 445 | 7(8)-EpDPE | Lipids and lipid-like molecules | Fatty Acyls | Fatty acids and conjugates | Level 2 | C22H32O3 | 0.83321106 | 0.415545406 | 1.333802821 | Up | 0.038135821 | 0.196469793 |
| 446 | PC(20:3-OH/P-18:1) | Lipids and lipid-like molecules | Glycerophospholipids | Glycerophosphocholines | Level 1 | C46H84NO8P | 3.770707355 | -7.304615786 | 0.006325452 | Down | 0.038545476 | 0.197577649 |
| 447 | Norepinephrine 3-sulfate | Organic acids and derivatives | Organic sulfuric acids and derivatives | Arylsulfates | Level 3 | C8H11NO6S | 0.897407132 | -0.445312367 | 0.734425284 | Down | 0.03863804 | 0.197577649 |
| 448 | 7-Oxooctanoylcarnitine | Lipids and lipid-like molecules | Fatty Acyls | Fatty acid esters | Level 4 | C15H27NO5 | 0.953331091 | 0.552886011 | 1.467017429 | Up | 0.038651026 | 0.197577649 |
| 449 | Etiocholanolone | Lipids and lipid-like molecules | Steroids and steroid derivatives | Androstane steroids | Level 2 | C19H30O2 | 1.136307705 | 0.813154219 | 1.757048749 | Up | 0.038683624 | 0.197577649 |
| 450 | DG(PGJ2/2:0/0:0) | Lipids and lipid-like molecules | Fatty Acyls | Eicosanoids | Level 2 | C25H38O7 | 1.757892984 | 1.662647989 | 3.165970886 | Up | 0.038958795 | 0.198556093 |
| 451 | Glu-Phe-Thr | Organic acids and derivatives | Carboxylic acids and derivatives | Amino acids, peptides, and analogues | Level 2 | C18H25N3O7 | 2.19083761 | -2.510973798 | 0.175437152 | Down | 0.039082822 | 0.198761677 |
| 452 | tetrazoline | Organoheterocyclic compounds | Azolines | Tetrazolines | Level 4 | CH4N4 | 2.732437278 | 3.895978547 | 14.88697315 | Up | 0.039281827 | 0.199346877 |
| 453 | 8Z,11Z,14Z,17Z-octadecatetraenoic acid | Lipids and lipid-like molecules | Fatty Acyls | Lineolic acids and derivatives | Level 1 | C18H28O2 | 1.057168191 | 0.657895544 | 1.577779441 | Up | 0.039462802 | 0.199498449 |
| 454 | PC(16:0/18:1-2OH) | Lipids and lipid-like molecules | Glycerophospholipids | Glycerophosphocholines | Level 3 | C42H82NO10P | 1.043212065 | 0.64191204 | 1.560395821 | Up | 0.039576427 | 0.199498449 |
| 455 | 14,15-epoxy,16-HETE | Lipids and lipid-like molecules | Fatty Acyls | Fatty acids and conjugates | Level 2 | C20H30O4 | 0.860475825 | 0.443574733 | 1.359969911 | Up | 0.039598126 | 0.199498449 |
| 456 | Sinapic acid | Phenylpropanoids and polyketides | Cinnamic acids and derivatives | Hydroxycinnamic acids and derivatives | Level 2 | C11H12O5 | 1.21331335 | -0.847770223 | 0.555642854 | Down | 0.039739223 | 0.199498449 |
| 457 | D-Maltose | Organic oxygen compounds | Organooxygen compounds | Carbohydrates and carbohydrate conjugates | Level 2 | C12H22O11 | 1.302604976 | 0.976871074 | 1.968192149 | Up | 0.039741789 | 0.199498449 |
| 458 | Dodecylamine | Organic nitrogen compounds | Organonitrogen compounds | Amines | Level 4 | C12H27N | 0.826486086 | -0.411263532 | 0.751964505 | Down | 0.039968696 | 0.199530052 |
| 459 | 1alpha,25-dihydroxy-11beta-phenylvitamin D3 | Lipids and lipid-like molecules | Steroids and steroid derivatives | Vitamin D and derivatives | Level 3 | C33H48O3 | 1.104862634 | -0.645755954 | 0.639157792 | Down | 0.04002498 | 0.199530052 |
| 460 | 2-Methylglutaric acid | Lipids and lipid-like molecules | Fatty Acyls | Fatty acids and conjugates | Level 2 | C6H10O4 | 1.012346395 | -0.515980373 | 0.699317555 | Down | 0.040158048 | 0.199530052 |
| 461 | POB-PC | Lipids and lipid-like molecules | Glycerophospholipids | Glycerophosphocholines | Level 2 | C28H54NO9P | 1.098902216 | 0.680504478 | 1.602700086 | Up | 0.040411718 | 0.200371252 |
| 462 | Santene | Hydrocarbons | Unsaturated hydrocarbons | Branched unsaturated hydrocarbons | Level 4 | C9H14 | 1.171534227 | 0.796240357 | 1.736569749 | Up | 0.040622559 | 0.200895554 |

| | | | | | | | | | | | | | |
|---|---|---|---|---|---|---|---|---|---|---|---|---|---|
| 463 | 3'-AMP | Nucleosides, nucleotides, and analogues | Ribonucleoside 3'-phosphates | Unclassified | Level 2 | C10H14N5O7P | 1.251538692 | 0.822526862 | 1.768500788 | Up | 0.040763936 | 0.200895554 |
| 464 | delta9-12-IsoF | Lipids and lipid-like molecules | Fatty Acyls | Fatty acids and conjugates | Level 2 | C20H34O6 | 1.076035859 | 0.667208886 | 1.587997769 | Up | 0.040771224 | 0.200895554 |
| 465 | Candoxatrilat | Organic acids and derivatives | Carboxylic acids and derivatives | Dicarboxylic acids and derivatives | Level 2 | C20H33NO7 | 1.330561901 | 1.04819797 | 2.067945222 | Up | 0.041711475 | 0.20510301 |
| 466 | N-(6-Aminohexyl)-1-Naphthalenesulfonamide | Benzenoids | Naphthalenes | Naphthalene sulfonic acids and derivatives | Level 4 | C16H22N2O2S | 0.991497485 | -0.496969032 | 0.708593908 | Down | 0.042238323 | 0.206047623 |
| 467 | PC(4:0/18:0) | Lipids and lipid-like molecules | Glycerophospholipids | Glycerophosphocholines | Level 3 | C30H60NO8P | 1.458121233 | -1.164872746 | 0.446003598 | Down | 0.042250607 | 0.206047623 |
| 468 | 5-Acetoxydihydrotheaespirane | Organoheterocyclic compounds | Tetrahydrofurans | Unclassified | Level 2 | C15H26O3 | 2.03949408 | 2.386693402 | 5.2295739 | Up | 0.04247683 | 0.206726377 |
| 469 | N-Acetylneuraminic acid | Organic oxygen compounds | Organooxygen compounds | Carbohydrates and carbohydrate conjugates | Level 1 | C11H19NO9 | 0.685585779 | 0.301960889 | 1.232818904 | Up | 0.042849011 | 0.20806934 |
| 470 | Dinor-alpha-muricholic acid | Lipids and lipid-like molecules | Steroids and steroid derivatives | Bile acids, alcohols and derivatives | Level 3 | C22H36O5 | 0.938307794 | 0.523012125 | 1.436952253 | Up | 0.042992292 | 0.20806934 |
| 471 | Cer(d18:1/18:0) | Lipids and lipid-like molecules | Sphingolipids | Ceramides | Level 1 | C36H71NO3 | 1.885352075 | 1.999660141 | 3.999057821 | Up | 0.043020136 | 0.20806934 |
| 472 | Dopamine | Benzenoids | Phenols | Benzenediols | Level 2 | C8H11NO2 | 1.222349346 | -0.821813353 | 0.565730417 | Down | 0.043103206 | 0.20806934 |
| 473 | N-stearoyl valine | Organic acids and derivatives | Carboxylic acids and derivatives | Amino acids, peptides, and analogues | Level 1 | C23H45NO3 | 1.23232337 | 0.822730322 | 1.768750214 | Up | 0.043369655 | 0.208930892 |
| 474 | N-Acetyl-L-methionine | Organic acids and derivatives | Carboxylic acids and derivatives | Amino acids, peptides, and analogues | Level 1 | C7H13NO3S | 1.042928563 | -0.552800801 | 0.681695422 | Down | 0.043669476 | 0.209626227 |
| 475 | delta9-Tetrahydrocannabinol hemisuccinate | Organoheterocyclic compounds | Benzopyrans | 1-benzopyrans | Level 2 | C25H34O5 | 1.071123361 | 0.67350321 | 1.594941169 | Up | 0.04373548 | 0.209626227 |
| 476 | Melatonin glucuronide | Nucleosides, nucleotides, and analogues | Nucleoside and nucleotide analogues | 1-pyranosylindoles | Level 2 | C19H24N2O8 | 1.076720788 | 0.698781649 | 1.623133483 | Up | 0.04386349 | 0.209626227 |
| 477 | Spermidine | Organic nitrogen compounds | Organonitrogen compounds | Amines | Level 1 | C7H19N3 | 1.148877453 | 0.769993257 | 1.705261813 | Up | 0.043953441 | 0.209626227 |
| 478 | L-Glutamic acid 5-phosphate | Organic acids and derivatives | Carboxylic acids and derivatives | Amino acids, peptides, and analogues | Level 2 | C5H10NO7P | 0.994227316 | -0.564336472 | 0.676266375 | Down | 0.044080728 | 0.209626227 |
| 479 | Dehydroascorbic acid | Organoheterocyclic compounds | Lactones | Gamma butyrolactones | Level 2 | C6H6O6 | 1.301538325 | 0.995168539 | 1.993313375 | Up | 0.044107792 | 0.209626227 |
| 480 | Perulactone | Lipids and lipid-like molecules | Steroids and steroid derivatives | Bile acids, alcohols and derivatives | Level 2 | C30H46O7 | 2.706080365 | -4.658710253 | 0.039590271 | Down | 0.044532916 | 0.209626227 |
| 481 | Propionylcarnitine | Lipids and lipid-like molecules | Fatty Acyls | Fatty acid esters | Level 1 | C10H20NO4+ | 1.122687918 | 0.762779826 | 1.696756836 | Up | 0.044578926 | 0.209626227 |
| 482 | Kifunensine | Organic acids and derivatives | Carboxylic acids and derivatives | Amino acids, peptides, and analogues | Level 1 | C8H12N2O6 | 0.918628638 | -0.466825191 | 0.72355511 | Down | 0.04459646 | 0.209626227 |
| 483 | Nimustine | Organoheterocyclic compounds | Diazines | Pyrimidines and pyrimidine derivatives | Level 3 | C9H13ClN6O2 | 0.802260939 | -0.411650548 | 0.75176281 | Down | 0.044700792 | 0.209626227 |
| 484 | 3-Hydroxy-3-carboxymethyl-adipic acid | Organic acids and derivatives | Carboxylic acids and derivatives | Tricarboxylic acids and derivatives | Level 4 | C8H12O7 | 1.061106694 | 0.743401315 | 1.674118111 | Up | 0.044706137 | 0.209626227 |
| 485 | Hydroxypelenolide | Lipids and lipid-like molecules | Prenol lipids | Terpene lactones | Level 2 | C15H24O3 | 1.194953687 | -0.834379792 | 0.560824083 | Down | 0.044737226 | 0.209626227 |
| 486 | Variabilin | Lipids and lipid-like molecules | Prenol lipids | Diterpenoids | Level 4 | C25H34O4 | 0.767943254 | -0.323052717 | 0.79937662 | Down | 0.04477789 | 0.209626227 |
| 487 | Sedoheptulose | Organic oxygen compounds | Organooxygen compounds | Carbohydrates and carbohydrate conjugates | Level 2 | C7H14O7 | 0.92318449 | 0.511473913 | 1.425505804 | Up | 0.044837947 | 0.209626227 |
| 488 | Tigloidine | Alkaloids and derivatives | Tropane alkaloids | Unclassified | Level 4 | C13H21NO2 | 2.172451895 | 2.440504489 | 5.428315178 | Up | 0.045233713 | 0.211061038 |
| 489 | Adenosine, 8-(butylamino)-N-cyclopentyl- | Nucleosides, nucleotides, and analogues | Purine nucleosides | Unclassified | Level 4 | C19H30N6O4 | 1.239901509 | 0.932049329 | 1.907984339 | Up | 0.045490706 | 0.211843975 |
| 490 | Chenodeoxycholyltaurine | Lipids and lipid-like molecules | Steroids and steroid derivatives | Bile acids, alcohols and derivatives | Level 3 | C26H45NO6S | 2.926317834 | -5.504627828 | 0.022026318 | Down | 0.045768595 | 0.212720967 |
| 491 | Adrenoylcarnitine | Lipids and lipid-like molecules | Fatty Acyls | Fatty acids and conjugates | Level 2 | C29H49NO4 | 1.031126855 | -0.557811187 | 0.679332044 | Down | 0.046030755 | 0.213044937 |
| 492 | Erythrono-1,4-lactone | Organoheterocyclic compounds | Lactones | Gamma butyrolactones | Level 2 | C4H6O4 | 0.677043699 | 0.283501609 | 1.217145473 | Up | 0.046068185 | 0.213044937 |
| 493 | Tetrahydrocortisol | Lipids and lipid-like molecules | Steroids and steroid derivatives | Hydroxysteroids | Level 2 | C21H34O5 | 2.175997508 | 2.598562687 | 6.056829026 | Up | 0.046107409 | 0.213044937 |
| 494 | 5-Methylthioribulose 1-phosphate | Organic oxygen compounds | Organooxygen compounds | Carbonyl compounds | Level 2 | C6H13O7PS | 0.943682219 | 0.535651368 | 1.449596491 | Up | 0.046554448 | 0.21457099 |
| 495 | Pentadecylamine | Organic nitrogen compounds | Organonitrogen compounds | Amines | Level 4 | C15H33N | 1.209159382 | 0.878441903 | 1.838388786 | Up | 0.046618371 | 0.21457099 |
| 496 | 3'-Hydroxy-T2 Toxin | Lipids and lipid-like molecules | Prenol lipids | Sesquiterpenoids | Level 2 | C24H34O10 | 2.666646171 | -4.37905149 | 0.048058936 | Down | 0.046813383 | 0.215051806 |
| 497 | 9K,12,13-diHODE | Lipids and lipid-like molecules | Fatty Acyls | Lineolic acids and derivatives | Level 2 | C18H30O5 | 1.430671736 | -1.27357532 | 0.413633426 | Down | 0.047291054 | 0.216826744 |
| 498 | 9-tetradecynoic acid | Lipids and lipid-like molecules | Fatty Acyls | Fatty acids and conjugates | Level 2 | C14H24O2 | 0.822112761 | 0.409183191 | 1.327933766 | Up | 0.047918686 | 0.218859381 |
| 499 | Octadecylamine | Organic nitrogen compounds | Organonitrogen compounds | Amines | Level 4 | C18H39N | 0.82855572 | 0.378891594 | 1.300342433 | Up | 0.048253119 | 0.219542448 |
| 500 | alpha-artemisic acid | Lipids and lipid-like molecules | Fatty Acyls | Lineolic acids and derivatives | Level 2 | C18H32O3 | 0.761090905 | 0.345034657 | 1.270181495 | Up | 0.048585798 | 0.220633404 |
| 501 | 2,4,6(1H,3H,5H)-Pyrimidinetrione, 5-ethyl-5-(3-hydroxy-1-methylbutyl)- | Organoheterocyclic compounds | Diazines | Pyrimidines and pyrimidine derivatives | Level 4 | C11H18N2O4 | 1.621372489 | 1.410461769 | 2.65822232 | Up | 0.048719923 | 0.220747919 |
| 502 | gamma-Glutamylcysteine | Organic acids and derivatives | Carboxylic acids and derivatives | Amino acids, peptides, and analogues | Level 2 | C8H14N2O5S | 0.794663102 | 0.394736159 | 1.314702304 | Up | 0.048796908 | 0.220747919 |
| 503 | Loracarbef | Organoheterocyclic compounds | Lactams | Beta lactams | Level 4 | C16H16ClN3O4 | 1.873896114 | 1.843002529 | 3.587558928 | Up | 0.049002757 | 0.221257695 |
| 504 | Cyclo(gly-pro) | Organic acids and derivatives | Carboxylic acids and derivatives | Amino acids, peptides, and analogues | Level 2 | C7H10N2O2 | 0.769941443 | -0.316503305 | 0.803013805 | Down | 0.049348242 | 0.222335703 |
| 505 | Trigonelline | Alkaloids and derivatives | Unclassified | Unclassified | Level 2 | C7H7NO2 | 0.904471542 | 0.509059653 | 1.423122304 | Up | 0.049532001 | 0.222335703 |
| 506 | 2,4-dihydroxy-2-heptenedioc acid | Organic acids and derivatives | Hydroxy acids and derivatives | Medium-chain hydroxy acids and derivatives | Level 1 | C7H10O6 | 0.742206862 | 0.341293872 | 1.266892289 | Up | 0.049554901 | 0.222335703 |
| 507 | 5,6-epoxy-2-salicyloylamino-2-cyclohexen-1,4-dione | Benzenoids | Phenols | 1-hydroxy-4-unsubstituted benzenoids | Level 4 | C13H9NO5 | 0.955345743 | -0.472601776 | 0.72066377 | Down | 0.049615967 | 0.222335703 |
| 508 | N-lactoyl-Tryptophan | Organic acids and derivatives | Carboxylic acids and derivatives | Amino acids, peptides, and analogues | Level 2 | C14H16N2O4 | 1.134620634 | -0.770297247 | 0.586296664 | Down | 0.049990331 | 0.223591405 |
